# Safety and efficacy of antioxidant therapy in children and adolescents with attention deficit hyperactivity disorder: A systematic review and network meta-analysis

**DOI:** 10.1371/journal.pone.0296926

**Published:** 2024-03-28

**Authors:** Peike Zhou, Xiaohui Yu, Tao Song, Xiaoli Hou

**Affiliations:** Department of Pediatrics, Affiliated ZhongShan Hospital of Dalian University, Dalian, Liaoning, China; Instituto Dermopatico dell’Immacolata, IDI-IRCCS, ITALY

## Abstract

**Objective:**

To systematically evaluate the safety and efficacy of antioxidant therapy in children and adolescents with attention deficit hyperactivity disorder (ADHD).

**Methods:**

Randomized controlled trials and prospective studies on antioxidant therapy in children and adolescents with ADHD were searched in PubMed, Embase, and Cochrane Library from the inception of databases to November 12, 2022. Two investigators independently screened the literature, extracted data, and evaluated the quality of the included studies. Network meta-analysis (PROSPERO registration number CRD 42023382824) was carried out by using R Studio 4.2.1.

**Results:**

48 studies involving 12 antioxidant drugs (resveratrol, pycnogenol, omega-3, omega-6, quercetin, phosphatidylserine, almond, vitamin D, zinc, folic acid, ginkgo biloba, Acetyl-L-carnitine) were finally included, with 3,650 patients. Network meta-analysis showed that omega-6 (0.18), vitamin D (0.19), and quercetin (0.24) were the top three safest drugs according to SUCRA. The omega-3 (SUCRA 0.35), pycnogenol (SUCRA 0.36), and vitamin D (SUCRA 0.27) were the most effective in improving attention, hyperactivity, and total score of Conners’ parent rating scale (CPRS), respectively. In terms of improving attention, hyperactivity, and total score of Conners’ teacher rating scale (CTRS), pycnogenol (SUCRA 0.32), phosphatidylserine+omega-3 (SUCRA 0.26), and zinc (SUCRA 0.34) were the most effective, respectively. In terms of improving attention, hyperactivity and total score of ADHD Rating Scale-Parent, the optimal agents were phosphatidylserine (SUCRA 0.39), resveratrol+MPH (SUCRA 0.24), and phosphatidylserine (SUCRA 0.34), respectively. In terms of improving attention, hyperactivity and total score of ADHD Rating Scale-Teacher, pycnogenol (SUCRA 0.32), vitamin D (SUCRA 0.31) and vitamin D (SUCRA 0.18) were the optimal agents, respectively. The response rate of omega-3+6 was the highest in CGI (SUCRA 0.95) and CPT (SUCRA 0.42).

**Conclusion:**

The rankings of safety and efficacy of the 12 antioxidants vary. Due to the low methodological quality of the included studies, the probability ranking cannot fully explain the clinical efficacy, and the results need to be interpreted with caution. More high-quality studies are still needed to verify our findings.

## Introduction

Attention deficit hyperactivity disorder (ADHD) is a persistent neurodevelopmental condition that typically occurs in childhood and often persists into adulthood [1]. It is associated with various behavioral challenges during childhood, and the extent of these challenges can serve as an independent predictor of antisocial personality disorder in adulthood [2]. Consequently, effectively managing behavioral issues in children can help alleviate the future burden on both families and society at large [3]. The etiology of ADHD is still unclear and may be attributable to the complex interactions between multiple factors [4–6].

ADHD is influenced by both genetic and environmental factors [7]. Genetic susceptibility is uncontrollable, and a bad social and family environment also increases the risk of behavioral problems [8, 9]. ADHD is related to prenatal smoking and exposure to toxic substances [10, 11]. Harmful environments can significantly increase reactive oxygen species (ROS) levels in patients [12]. Psychological factors can also induce ADHD [13]. Adverse experiences in early childhood or school age can lead to emotional problems such as anxiety and depression, which gradually develop into ADHD in adolescence [14–16]. A study has found obvious changes in levels of reactive oxidation products in patients with anxiety [17]. The total oxidant status (TOS) and oxidative stress index (OSI) were higher in depressed patients [18]. In addition, dopamine, the major neurotransmitter responsible for ADHD [19], has also been reported to be affected by ROS levels [20, 21]. These studies indicate that oxidative stress may be one of the potential biological mechanisms leading to ADHD. Oxidative stress disrupts the structure and function of neurons in the prefrontal lobe of the brain [22]. Structural and functional impairments in the prefrontal cortex have been shown to be highly correlated with behavioral and emotional problems of ADHD [23]. Therefore, the application of antioxidant therapy is gradually accepted in clinical practice.

While first-line drug therapies, such as the central stimulant MPH, can effectively manage certain behavioral problems, they may induce numerous adverse reactions and cause long-term drug dependence [24, 25]. Non-pharmacological interventions, such as cognitive behavioral therapy (CBT), have also demonstrated their effectiveness [13]. However, they are associated with extended treatment durations and an increased financial burden on families. Consequently, safer and more cost-effective antioxidant options are needed.

Currently, a variety of antioxidant drugs are used in the treatment of children and adolescents with ADHD. Nevertheless, these drugs vary in their therapeutic mechanisms and lack direct comparisons. Therefore, this study employs a network meta-analysis approach to compare the safety and effectiveness of commonly utilized antioxidant drugs in clinical practice. The aim is to provide an evidence-based foundation for the selection of superior antioxidant medications in clinical settings.

## Data and method

This systematic review adheres rigorously to the PRISMA Guideline [26] (S1 Checklist) and follows the Cochrane System Review Manual [27]. It is registered with PROSPERO under the registration number CRD 42023382824 (S1 File).

### Inclusion criteria

The inclusion criteria were meticulously defined in accordance with the PICOS principles: (1) Subjects: The study included children and adolescents with ADHD who met the diagnostic criteria of DSM-III/IV/5 [28] or were clinically diagnosed. The age of the participants was 18 years or younger, without regard to gender. (2) Intervention Measures: The experimental group received antioxidant drugs, including but not limited to quercetin, ginkgo, zinc, vitamins, unsaturated fatty acids, and folic acid. The minimum treatment duration was set at 2 weeks. The control group was treated with either a placebo, MPH, or antioxidants alone, or in combination. There were no restrictions on the route of administration, dosing, or treatment regimen. (3) Primary Outcome Indicators: The primary outcomes included safety and efficacy assessments. Safety was determined by monitoring the number of adverse events (comprising all adverse symptoms reported from the beginning to the end of the study). Efficacy was evaluated using Conners’ parent rating scale (CPRS), Conners’ teacher rating scale (CTRS), ADHD rating scale-parent (ADHD RS-Parent), and ADHD rating scale-teacher (ADHD RS-Teacher), which encompassed assessments of attention, hyperactivity, and total scores. Additionally, secondary outcome indicators such as the Clinical Global Impressions scale (CGI) and Continuous Performance Test (CPT) were also employed to gauge effectiveness (S8 Table). (4) Study Type: The study considered randomized controlled trials and prospective studies.

### Exclusion criteria

The exclusion criteria were as follows: (1) Patients suffered from other serious diseases, such as epilepsy or systemic lupus erythematosus; (2) Antioxidant drugs were obtained from daily diet rather than drug supplements; (3) Antioxidant drugs combined with other non-drug treatments were taken as an intervention (e.g. behavioral therapy, mindfulness intervention [29]); (4) Self-control study, review or mechanistic study.

### Search strategy

A computerized search was conducted in the PubMed, Embase, and Cochrane Library databases from the inception of the databases to November 12, 2022. The search terms included (’Attention Deficit Hyperactivity Disorder’ OR ’ADHD’) AND (’children’ OR ’Adolescent’) AND (’Antioxidants’ OR ’Unsaturated Fatty Acids’ OR ’Zinc’ OR ’pycnogenols’ OR ’vitamin’ OR ’Quercetin’ OR ’Ginkgo’). The detailed search strategy is provided in S1 Table. Additionally, manual searches of references in the included studies were conducted. There were no language restrictions.

### Literature screening and data extraction

Two investigators independently screened literature using Endnote 20. They then proceeded to extract, encode, and cross-verify the data using Excel. In the event of any discrepancies or disagreements, a third investigator was consulted to facilitate consensus. The following information was extracted from the literature: title, author(s), publication year, study type, follow-up duration, participant count, treatment details (including medication and daily dosage), and evaluation outcomes. The baseline and endpoint data in trials were extracted. In cases where multiple time points were reported, the average value was extracted for analysis.

### Evaluation of risk of bias

Two investigators meticulously assessed the risk of bias for the included studies, strictly adhering to the Cochrane risk-of-bias tool. This tool comprises seven key items: random allocation, allocation concealment, blinding of intervention for both participants and medical staff, blinding of outcome assessment, integrity of result data, selective reporting of study outcomes, and potential sources of other bias. Each of these items was graded as either low risk, unclear, or high risk.

### Statistical analysis

The netmeta packages [127] in software R studio 4.2.1 was used to generate evidence network and probability ranking to present the direct and indirect comparison between different interventions. Network meta-analysis and heterogeneity test were carried out as part of the analysis. An I^2^ value of 50% was established as the critical threshold for selecting the appropriate effects model [128]. An I^2^ ≤ 50% indicated minimal heterogeneity, and the fixed-effects model was employed; otherwise, the random-effects model was chosen. Consistency between direct and indirect evidence was assessed using the nodal analysis method. Continuous variables were presented as mean differences (MD), and dichotomous variables were expressed as odds ratios (OR) along with 95% credibility intervals (CrI). To rank each intervention, the surface under the cumulative ranking curve (SUCRA) was utilized. The SUCRA value ranges from 0 to 1 [30]. The closer the value is to 0, the lower the probability of an event. Meanwhile, the closer the value is to 1, the higher the probability (S8 Table).

## Results

### Basic information on the included studies

**Fig 1:**
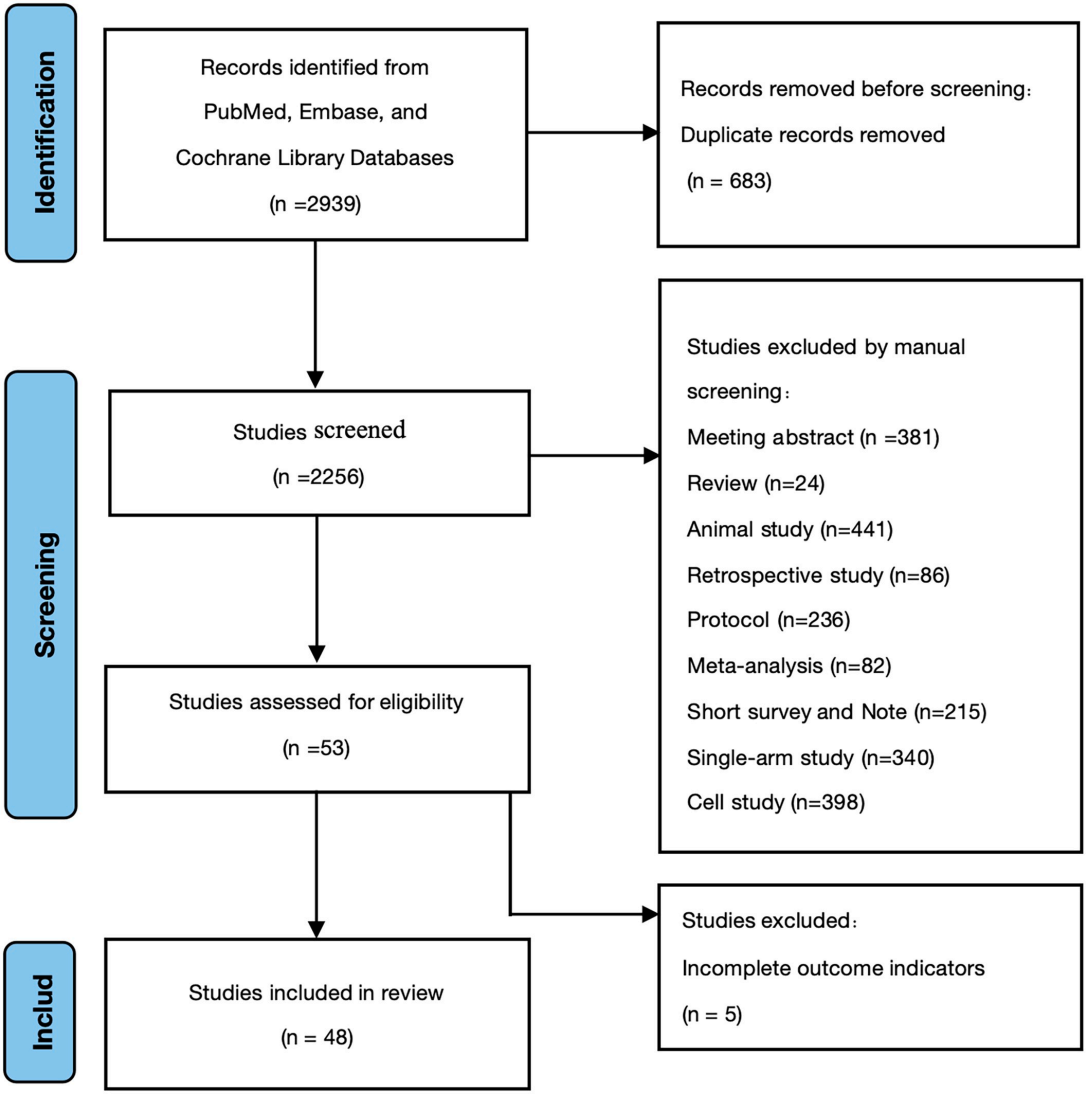
Flow diagram for study selection process.

Following the search strategy, a total of 2,939 studies were initially identified. After eliminating 683 duplicate records across various databases, the titles and abstracts of the remaining 2,203 studies were reviewed to exclude irrelevant studies, such as guidelines, reviews, animal experiments, and case reports. Subsequently, after a detailed examination of the full texts, 5 studies that lacked outcome indicators were excluded. Ultimately, 48 studies were included in the analysis [31–78], encompassing a total of 3,650 children. Among them, 1,930 cases were in the experimental group, and 1,720 cases were in the control group. These studies involved 12 antioxidant drugs, namely resveratrol, pycnogenol, unsaturated fatty acids (omega-3 and omega-6), quercetin, phosphatidylserine, almond, vitamin D, zinc, folic acid, ginkgo, and Acetyl-L-carnitine. The literature screening process is illustrated in Fig 1, and detailed information about the included studies can be found in S2 and S6 Tables.

### Safety

#### Evidence network and consistency test

**Fig 2:**
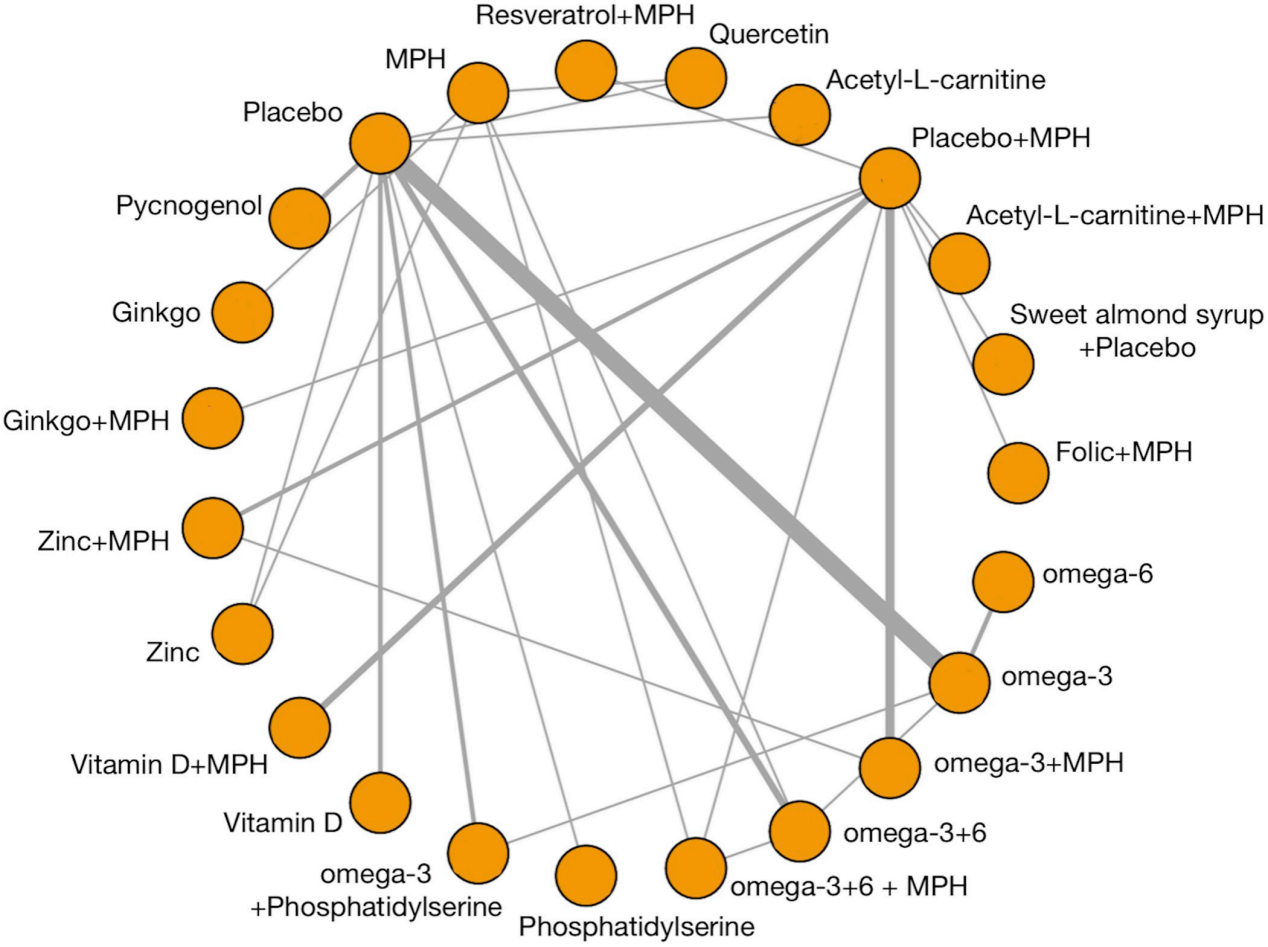
Evidence network for antioxidant therapy.

In the evidence network depicted in Fig 2, each node represents an intervention measure, and the thickness of the lines connecting nodes is proportional to the number of studies that involved those interventions. There were closed loops, necessitating nodal analysis to assess consistency. The evidence network encompassed 12 antioxidant drugs, involving a total of 3,141 patients from 41 studies. The closed loops primarily involved quercetin, zinc, omega-3, omega-6, phosphatidylserine, MPH, and placebo, both alone and in combination, forming ten groups for pairwise comparisons. Nodal analysis confirmed the consistency between the results of indirect comparisons and direct comparisons (S1A Fig). Importantly, these comparisons did not reveal statistically significant differences (P ≥ 0.05), underscoring the reliability of the results from the network meta-analysis.

#### Heterogeneity test and network meta-analysis

Heterogeneity test results showed that there was no significant heterogeneity (I^2^ < 50%) among the studies (S2A Fig). Therefore, the fixed-effects model was selected for analysis. Network meta-analysis (Table 1) on the safety of the included studies showed that omega-6, quercetin, omega-3, Acetyl-L-carnitine, and omega-3+6 were superior to ginkgo and omgea-3+6 + MPH; vitamin D+MPH was superior to Zinc+MPH and resveratrol+MPH; sweet almond syrup+placebo and Acetyl-L-carnitine+MPH were superior to resveratrol+MPH, zinc+MPH, and omega-3+MPH; omega-6 was superior to pycnogenol in terms of the safety of antioxidant drugs in children with ADHD (S3A Table).

**Table 1:** Network meta-analysis for safety of antioxidant therapy.

**Folic+MPH**	**-13.01** **(-42.09,-0.93)**	**-12.97** **(-42.11,-0.9)**	-0.44 (-2.04,1.01)	-12.49 (-45.98,14.53)	-12.75 (-46.22,14.23)	12.1 (0,41.32)	-12.07 (-45.54,14.88)	-12.41 (-45.91,14.59)	-6.43 (-40.91,22.84)	1.67 (-35.56,39.7)	-0.89 (-2.71,0.79)	7.09 (-0.37,25.78)	-11.61 (-45.14,15.3)	-0.72 (-2.35,0.75)	-22.46 (-65.81,13.6)	-12.29 (-45.77,14.7)	-19.84 (-64.82,18.6)	-6.71 (-39.2,19.2)	-12.47 (-45.91,14.51)	4.98 (-0.61,19.76)	-12.55 (-46.05,14.4)	-14 (-47.53,13.1)
**13.01** **(0.93,42.09)**	**sweet almond** **-syrup** **+ Placebo**	0 (-32.23,31.87)	**12.54** **(0.6,41.57)**	1.66 (-35.4,39.95)	1.38 (-35.67,39.67)	**27.64** **(5.17,65.56)**	2.07 (-34.97,40.39)	1.73 (-35.32,40.03)	7.88 (-30.27,47.63)	16.43 (-24.93,62.3)	12.09 (0.14,41.17)	**22.04** **(4.01,54.06)**	2.54 (-34.54,40.8)	**12.25** **(0.32,41.31)**	-7.76 (-54.72,37.3)	1.87 (-35.21,40.2)	-5.23 (-53.55,41.3)	7.3 (-28.45,44.92)	1.68 (-35.35,40)	**19.62** **(3.39,50.33)**	1.57 (-35.48,39.9)	0.21 (-37,38.51)
**12.97** **(0.9,42.11)**	0 (-31.87,32.23)	**Acetyl-L-Carnitine+MPH**	**12.51** **(0.58,41.64)**	1.7 (-35.55,39.85)	1.43 (-35.81,39.62)	**27.63** **(5.27,65.71)**	2.12 (-35.1,40.31)	1.77 (-35.49,39.98)	7.94 (-30.34,47.68)	16.44 (-25.04,62.2)	**12.06** **(0.11,41.23)**	**22.01** **(4.05,54.39)**	2.58 (-34.64,40.8)	**12.22** **(0.29,41.36)**	-7.82 (-54.85,36.8)	1.91 (-35.35,40.1)	-5.26 (-53.51,41.2)	7.3 (-28.91,44.73)	1.74 (-35.51,39.89)	**19.55** **(3.32,50.56)**	1.63 (-35.63,39.8)	0.21 (-37.18,38.4)
0.44 (-1.01,2.04)	**-12.54** **(-41.57,-0.6)**	**-12.51** **(-41.64,-0.58)**	**Placebo** **+ MPH**	-12.01 (-45.49,14.89)	-12.3 (-45.74,14.59)	**12.55** **(0.6,41.73)**	-11.63 (-45.07,15.25)	-11.93 (-45.41,14.92)	-5.99 (-40.36,23.26)	2.13 (-35.1,40.16)	-0.43 (-1.34,0.39)	**7.53** **(0.35,26.16)**	-11.12 (-44.61,15.8)	**-0.27** **(-0.53,-0.09)**	-22 (-65.37,14.1)	-11.82 (-45.28,15.1)	-19.39 (-64.33,19.1)	-6.26 (-38.76,19.56)	-12.02 (-45.45,14.87)	**5.4** **(0.26,20.13)**	-12.08 (-45.55,14.8)	-13.51 (-47.04,13.5)
12.49 (-14.53,45.9)	-1.66 (-39.95,35.4)	-1.7 (-39.85,35.55)	12.01 (-14.89,45.49)	**Acetyl-L** **-Carnitine**	-0.26 (-0.89,0.31)	26.67 (-5.93,68.26)	0.42 (-0.19,0.96)	0.07 (-0.17,0.32)	5.23 (-0.67,18.87)	**13.01** **(1.02,41.88)**	11.58 (-15.35,45.01)	21.12 (-8.34,57.64)	0.88 (0.47,1.3)	11.73 (-15.15,45.2)	-8.37 (-39.42,12.8)	0.18 (-0.65,1.11)	-6.11 (-38.73,19.5)	**4.25** **(0.46,14.5)**	0.03 (-0.59,0.59)	18.75 (-9.88,54.05)	-0.08 (-0.49,0.34)	-1.31 (-4.66,0.66)
12.75 (-14.23,46.2)	-1.38 (-39.67,35.67)	-1.43 (-39.62,35.81)	12.3 (-14.59,45.74)	0.26 (-0.31,0.89)	**Quercetin**	26.95 (-5.62,68.58)	**0.65** **(0.23,1.32)**	0.33 (-0.18,0.92)	5.51 (-0.41,19.11)	**13.27** **(1.28,42.15)**	11.87 (-15.07,45.33)	21.38 (-8.08,57.96)	**1.13** **(0.58,1.8)**	12.01 (-14.87,45.5)	-8.1 (-39.12,13.1)	0.45 (-0.5,1.52)	-5.83 (-38.44,19.8)	**4.51** **(0.75,14.75)**	0.27 (-0.22,0.95)	19.02 (-9.56,54.33)	0.18 (-0.43,0.86)	-1.04 (-4.44,1)
-12.1 (-41.32,0)	**-27.64** **(-65.56,-5.17)**	**-27.63** **(-65.71,-5.27)**	**-12.55** **(-41.73,-0.6)**	-26.67 (-68.26,5.93)	-26.95 (-68.58,5.62)	**Resveratrol** **+ MPH**	-26.26 (-67.92,6.34)	-26.6 (-68.22,5.98)	-20.43 (-62.96,14.02)	-12.2 (-57.08,29.9)	**-13** **(-42.16,-1.01)**	-4.41 (-35.2,18)	-25.78 (-67.41,6.82)	**-12.83** **(-42,-0.88)**	-36.7 (-86.48,4.43)	-26.46 (-68.12,6.13)	-34.18 (-85.2,8.95)	-20.94 (-61.62,10.78)	-26.64 (-68.3,5.98)	-6.38 (-36.46,12.9)	-26.74 (-68.36,5.84)	-28.14 (-69.94,4.57)
12.07 (-14.88,45.5)	-2.07 (-40.39,34.97)	-2.12 (-40.31,35.1)	11.63 (-15.25,45.07)	-0.42 (-0.96,0.19)	**-0.65** **(-1.32,-0.23)**	26.26 (-6.34,67.92)	**MPH**	-0.35 (-0.82,0.22)	4.82 (-1.07,18.47)	**12.58** **(0.6,41.49)**	11.2 (-15.74,44.62)	20.69 (-8.76,57.27)	**0.44** **(0.03,1.1)**	11.35 (-15.54,44.8)	-8.79 (-39.78,12.4)	-0.23 (-1.17,0.82)	-6.53 (-39.15,19.1)	**3.81** **(0.15,14.07)**	**-0.37** **(-0.67,-0.16)**	18.33 (-10.28,53.58)	-0.5 (-1.08,0.16)	-1.72 (-5.1,0.31)
12.41 (-14.59,45.9)	-1.73 (-40.03,35.32)	-1.77 (-39.98,35.49)	11.93 (-14.92,45.41)	-0.07 (-0.32,0.17)	-0.33 (-0.92,0.18)	26.6 (-5.98,68.22)	0.35 (-0.22,0.82)	**Placebo**	5.16 (-0.73,18.79)	**12.93** **(0.95,41.81)**	11.52 (-15.42,44.92)	21.04 (-8.4,57.56)	**0.8** **(0.49,1.14)**	11.66 (-15.21,45.1)	-8.43 (-39.47,12.7)	0.11 (-0.68,1)	-6.18 (-38.79,19.4)	**4.18** **(0.4,14.44)**	-0.04 (-0.61,0.46)	18.69 (-9.93,53.95)	-0.15 (-0.49,0.18)	-1.38 (-4.72,0.57)
6.43 (-22.84,40.9)	-7.88 (-47.63,30.27)	-7.94 (-47.68,30.34)	5.99 (-23.26,40.36)	-5.23 (-18.87,0.67)	-5.51 (-19.11,0.41)	20.43 (-14.02,62.9)	-4.82 (-18.47,1.07)	-5.16 (-18.79,0.73)	**Pycnogenol**	7.19 (-11.26,37.3)	5.52 (-23.72,39.92)	15.01 (-16.71,52.5)	-4.35 (-17.99,1.56)	5.7 (-23.57,40.1)	-14.85 (-47.29,8.3)	-5.06 (-18.67,0.93)	-12.52 (-46.84,14.8)	-0.51 (-15.36,11.79)	-5.21 (-18.85,0.69)	12.64 (-18.16,48.81)	-5.31 (-18.95,0.59)	**-6.8** **(-20.59,-0.2)**
-1.67 (-39.7,35.56)	-16.43 (-62.29,24.93)	-16.44 (-62.15,25.04)	-2.13 (-40.16,35.1)	**-13.01** **(-41.88,-1.02)**	**-13.27** **(-42.15,-1.28)**	12.2 (-29.86,57.0)	**-12.58** **(-41.49,-0.6)**	**-12.93** **(-41.81,-0.95)**	-7.19 (-37.26,11.26)	**Ginkgo**	-2.59 (-40.62,34.68)	6.79 (-33.02,47.1)	**-12.13** **(-41.04,-0.2)**	-2.41 (-40.44,34.8)	-23.56 (-63.12,4.22)	-12.79 (-41.74,-0.8)	-21.08 (-62.57,10.1)	-7.6 (-37.1,9.05)	**-12.98** **(-41.89,-1)**	4.5 (-34.64,43.56)	**-13.09** **(-41.97,-1.09)**	**-14.53** **(-43.51,-2.1)**
0.89 (-0.79,2.71)	**-12.09** **(-41.17,-0.14)**	**-12.06** **(-41.23,-0.11)**	0.43 (-0.39,1.34)	-11.58 (-45.01,15.35)	-11.87 (-45.33,15.07)	**13** **(1.01,42.16)**	-11.2 (-44.62,15.74)	-11.52 (-44.92,15.42)	-5.52 (-39.92,23.72)	2.59 (-34.68,40.6)	**Ginkgo+MPH**	7.97 (0.72,26.62)	-10.71 (-44.14,16.3)	0.15 (-0.7,1.09)	-21.57 (-64.87,14.5)	-11.39 (-44.8,15.57)	-18.93 (-63.79,19.6)	-5.83 (-38.32,20.04)	-11.59 (-44.99,15.37)	**5.85** **(0.58,20.62)**	-11.66 (-45.1,15.28)	-13.08 (-46.6,13.97)
-7.09 (-25.78,0.37)	**-22.04** **(-54.06,-4.01)**	**-22.01** **(-54.39,-4.05)**	**-7.53** **(-26.16,-0.35)**	-21.12 (-57.64,8.34)	-21.38 (-57.96,8.08)	4.41 (-18,35.2)	-20.69 (-57.27,8.76)	-21.04 (-57.56,8.4)	-15.01 (-52.51,16.71)	-6.79 (-47.14,33.1)	**-7.97** **(-26.62,-0.72)**	**Zinc+MPH**	-20.24 (-56.76,9.21)	**-7.81** **(-26.44,-0.6)**	-31.07 (-76.96,7.06)	-20.9 (-57.51,8.54)	-28.46 (-75.82,12.1)	-15.39 (-50.98,12.98)	-21.09 (-57.65,8.4)	-1.66 (-20.72,13.45)	-21.18 (-57.68,8.26)	-22.61 (-59.31,7.04)
11.61 (-15.37,45.14)	-2.54 (-40.88,34.54)	-2.58 (-40.77,34.64)	11.12 (-15.75,44.61)	**-0.88** **(-1.3,-0.47)**	-1.13 (-1.8,-0.58)	25.78 (-6.82,67.41)	**-0.44** **(-1.1,-0.03)**	**-0.8** **(-1.14,-0.49)**	4.35 (-1.56,17.99)	**12.13** **(0.14,41.04)**	10.71 (-16.26,44.14)	20.24 (-9.21,56.76)	**Zinc**	10.84 (-16.05,44.3)	-9.26 (-40.26,11.9)	-0.69 (-1.55,0.25)	-6.99 (-39.58,18.6)	3.37 (-0.41,13.6)	**-0.83** **(-1.49,-0.36)**	17.89 (-10.78,53.14)	**-0.95** **(-1.43,-0.5)**	**-2.18** **(-5.55,-0.21)**
0.72 (-0.75,2.35)	**-12.25** **(-41.31,-0.32)**	**-12.22** **(-41.36,-0.29)**	**0.27** **(0.09,0.53)**	-11.73 (-45.2,15.15)	-12.01 (-45.47,14.87)	**12.83** **(0.88,42)**	-11.35 (-44.75,15.54)	-11.66 (-45.13,15.21)	-5.7 (-40.09,23.57)	2.41 (-34.81,40.4)	-0.15 (-1.09,0.7)	**7.81** **(0.63,26.44)**	-10.84 (-44.31,16.1)	**Vitamin D** **+ MPH**	-21.74 (-65.1,14.36)	-11.54 (-44.99,15.4)	-19.11 (-64.04,19.4)	-5.98 (-38.49,19.87)	-11.73 (-45.13,15.17)	**5.68** **(0.53,20.43)**	-11.81 (-45.27,15.1)	-13.22 (-46.74,13.8)
22.46 (-13.64,65.8)	7.76 (-37.32,54.72)	7.82 (-36.75,54.85)	22 (-14.07,65.37)	8.37 (-12.8,39.42)	8.1 (-13.08,39.12)	36.7 (-4.43,86.48)	8.79 (-12.37,39.78)	8.43 (-12.72,39.47)	14.85 (-8.3,47.29)	23.56 (-4.22,63.12)	21.57 (-14.49,64.87)	31.07 (-7.06,76.96)	9.26 (-11.95,40.3)	21.74 (-14.36,65.1)	**Vitamin D**	8.56 (-12.6,39.61)	2.61 (-36.74,42.1)	14.24 (-8.58,46.47)	8.4 (-12.78,39.4)	28.61 (-8.64,73.52)	8.29 (-12.87,39.3)	6.93 (-14.48,37.9)
12.29 (-14.71,45.7)	-1.87 (-40.2,35.21)	-1.91 (-40.1,35.35)	11.82 (-15.07,45.28)	-0.18 (-1.11,0.65)	-0.45 (-1.52,0.5)	26.46 (-6.13,68.12)	0.23 (-0.82,1.17)	-0.11 (-1,0.68)	5.06 (-0.93,18.67)	12.79 (0.81,41.74)	11.39 (-15.57,44.8)	20.9 (-8.54,57.51)	0.69 (-0.25,1.55)	11.54 (-15.36,44.9)	-8.56 (-39.61,12.6)	**Phosphatidy-lserine** **+ omega-3**	-6.32 (-38.93,19.2)	**4.06** **(0.1,14.27)**	-0.16 (-1.21,0.79)	18.54 (-10.02,53.79)	-0.26 (-1.19,0.6)	-1.52 (-4.95,0.63)
19.84 (-18.64,64.8)	5.23 (-41.28,53.55)	5.26 (-41.15,53.51)	19.39 (-19.11,64.33)	6.11 (-19.5,38.73)	5.83 (-19.78,38.44)	34.18 (-8.95,85.2)	6.53 (-19.08,39.15)	6.18 (-19.43,38.79)	12.2 (-14.76,46.84)	21.08 (-10.04,62.6)	18.93 (-19.59,63.79)	28.46 (-12.03,75.8)	6.99 (-18.61,39.6)	19.11 (-19.41,64.1)	-2.61 (-42.04,36.7)	6.32 (-19.23,38.9)	**Phosphatidy-lserine**	11.96 (-14.73,45.44)	6.13 (-19.48,38.75)	26.09 (-13.56,72.37)	6.04 (-19.61,38.6)	4.64 (-21.1,37.45)
6.71 (-19.2,39.2)	-7.3 (-44.92,28.45)	-7.3 (-44.73,28.91)	6.26 (-19.56,38.76)	**-4.25** **(-14.5,-0.46)**	**-4.51** **(-14.75,-0.75)**	20.94 (-10.78,61.6)	**-3.81** **(-14.07,-0.15)**	**-4.18** **(-14.44,-0.4)**	0.51 (-11.79,15.36)	7.6 (-9.05,37.1)	5.83 (-20.04,38.32)	15.39 (-12.98,50.9)	-3.37 (-13.6,0.41)	5.98 (-19.87,38.5)	-14.24 (-46.47,8.58)	**-4.06** **(-14.27,-0.1)**	-11.96 (-45.44,14.7)	**omega-3+6** **+ MPH**	**-4.19** **(-14.48,-0.53)**	12.98 (-14.52,47.36)	**-4.34** **(-14.6,-0.53)**	**-5.96** **(-16.28,-1)**
12.47 (-14.51,45.9)	-1.68 (-40,35.35)	-1.74 (-39.89,35.51)	12.02 (-14.87,45.45)	-0.03 (-0.59,0.59)	-0.27 (-0.95,0.22)	26.64 (-5.98,68.3)	**0.37** **(0.16,0.67)**	0.04 (-0.46,0.61)	5.21 (-0.69,18.85)	**12.98** **(1,41.89)**	11.59 (-15.37,44.99)	21.09 (-8.4,57.65)	**0.83** **(0.36,1.49)**	11.73 (-15.17,45.2)	-8.4 (-39.4,12.78)	0.16 (-0.79,1.21)	-6.13 (-38.75,19.5)	**4.19** **(0.53,14.48)**	**omega-3+6**	18.73 (-9.91,53.98)	-0.11 (-0.72,0.55)	-1.34 (-4.71,0.7)
-4.98 (-19.76,0.61)	**-19.62** **(-50.33,-3.39)**	**-19.55** **(-50.56,-3.32)**	**-5.4** **(-20.13,-0.26)**	-18.75 (-54.05,9.88)	-19.02 (-54.33,9.56)	6.38 (-12.9,36.46)	-18.33 (-53.58,10.28)	-18.69 (-53.95,9.93)	-12.64 (-48.81,18.16)	-4.5 (-43.56,34.6)	**-5.85** **(-20.62,-0.58)**	1.66 (-13.45,20.7)	-17.89 (-53.14,10.8)	**-5.68** **(-20.43,-0.5)**	-28.61 (-73.52,8.64)	-18.54 (-53.79,10.1)	-26.09 (-72.37,13.6)	-12.98 (-47.36,14.52)	-18.73 (-53.98,9.91)	**omega-3** **+ MPH**	-18.82 (-54.1,9.8)	-20.2 (-55.61,8.54)
12.55 (-14.42,46.0)	-1.57 (-39.91,35.48)	-1.63 (-39.79,35.63)	12.08 (-14.8,45.55)	0.08 (-0.34,0.49)	-0.18 (-0.86,0.43)	26.74 (-5.84,68.36)	0.5 (-0.16,1.08)	0.15 (-0.18,0.49)	5.31 (-0.59,18.95)	**13.09** **(1.09,41.97)**	11.66 (-15.28,45.1)	21.18 (-8.26,57.68)	**0.95** **(0.5,1.43)**	11.81 (-15.05,45.3)	-8.29 (-39.31,12.9)	0.26 (-0.6,1.19)	-6.04 (-38.63,19.6)	**4.34** **(0.53,14.6)**	0.11 (-0.55,0.72)	18.82 (-9.8,54.1)	**omega-3**	-1.22 (-4.57,0.7)
14 (-13.1,47.53)	-0.21 (-38.51,37)	-0.21 (-38.41,37.18)	13.51 (-13.5,47.04)	1.31 (-0.66,4.66)	1.04 (-1,4.44)	28.14 (-4.57,69.94)	1.72 (-0.31,5.1)	1.38 (-0.57,4.72)	**6.8** **(0.19,20.59)**	**14.53** **(2.01,43.51)**	13.08 (-13.97,46.6)	22.61 (-7.04,59.31)	**2.18** **(0.21,5.55)**	13.22 (-13.77,46.7)	-6.93 (-37.98,14.5)	1.52 (-0.63,4.95)	-4.64 (-37.45,21.1)	**5.96** **(1,16.28)**	1.34 (-0.7,4.71)	20.2 (-8.54,55.61)	1.22 (-0.7,4.57)	**omega-6**

Note: significant results are in bold and underscored. OR = Odds ratio, CrI = credibility interval. All results are presented as Lg [OR (95% CrI)].

#### Probability ranking

The ranking of SUCRA values is shown in Table 2. The top three safest antioxidant drugs were omega-6 (0.18), vitamin D (0.19), and quercetin (0.24) (S4A Table).

**Table 2:** Probability ranking for safety of antioxidant therapy.

Intervention	SUCRA	Rank	Intervention	SUCRA	Rank
Folic+MPH	0.70	19	Zinc+MPH	0.88	22
Sweet almond syrup+Placebo	0.31	7	Zinc	0.54	13
Acetyl-L-carnitine+MPH	0.32	6	Vitamin D+MPH	0.62	15
Placebo+MPH	0.68	18	Vitamin D	0.19	2
Acetyl-L-carnitine	0.32	8	omega-3+Phosphatidylserine	0.38	11
Quercetin	0.24	3	Phosphatidylserine	0.25	4
Resveratrol+MPH	0.92	23	omega-3+6 + MPH	0.65	16
MPH	0.46	12	omega-3+6	0.33	9
Placebo	0.36	10	omega-3+MPH	0.85	21
Pycnogenol	0.64	17	omega-3	0.29	5
Ginkgo	0.77	20	omega-6	0.18	1
Ginkgo+MPH	0.61	14			

Note: MPH = Methylphenidate; omega-3 = omega-3 fatty acids; omega-6 = omega-6 fatty acids; omega-3+6 = omega-3 fatty acids+omega-6 fatty acids.

### Conners’ parent rating scale (CPRS)

#### Evidence network and consistency test

The evidence network displayed closed loops within the network relationships (S3B–S3E Fig). To ensure the consistency of the results, nodal analysis was conducted. For the attention score evidence network, there were 5 antioxidant drugs involved in 9 studies, encompassing a total of 761 patients. The hyperactivity score evidence network included 5 antioxidant drugs in 9 studies, with a total of 729 patients. Regarding the total score, there were two evidence networks: Network A, which included 5 antioxidant drugs, 12 studies, and 928 patients, and Network B, comprising 4 antioxidant drugs, 5 studies, and 428 patients. Nodal analysis demonstrated that the results from both indirect and direct comparisons were consistent, and no statistically significant differences were observed (P ≥ 0.05). These findings underscore the high reliability of the results obtained through network meta-analysis (S1B and S1C Fig).

#### Heterogeneity test and network meta-analysis

Heterogeneity testing was performed individually on attention, hyperactivity, and total score, and the results indicated that the heterogeneity among the studies was minimal (I^2^ < 50%) across the studies (S2B–S2E Fig). Consequently, the fixed-effects model was employed for the analysis. The network meta-analysis showed no statistically significant differences in efficacy between various interventions (S3B–S3E Table).

#### Probability ranking

The results showed that the top three antioxidant drugs in the improvement of CPRS score were as follows: (i) omega-3 (0.35), phosphatidylserine+omega-3 (0.38), and Acetyl-L-carnitine (0.47) were the most effective for improving attention; (ii) Pycnogenol (0.36), phosphatidylserine+omega-3 (0.42), and omega-3 (0.44) were the most effective for improving hyperactivity; (iii) vitamin D (0.27), phosphatidylserine+omega-3 (0.39), and omega-3+6 (0.53) were the optimal agents for improving total score (network A); (iv) zinc+MPH (0.43), vitamin D+MPH (0.46), and folic+MPH (0.52) were the most effective for improving total score (network B) (S4B–S4E Table).

### Conners’ teacher rating scale (CTRS)

#### Evidence network

The evidence network of attention score included 4 antioxidant drugs in 5 studies, with 524 patients. The evidence network of hyperactivity score included 5 antioxidant drugs in 6 studies, with 864 patients. The evidence network of total score included 5 antioxidant drugs in 8 studies, with 930 patients (S3F–S3H Fig).

#### Heterogeneity test and network meta-analysis

Heterogeneity test was carried out on attention, hyperactivity and total score, respectively, and the results showed that the heterogeneity among the studies was small (I^2^ < 50%) (S2F and S2G Fig). Therefore, the fixed-effects model was selected for analysis. The network meta-analysis showed no statistical differences in efficacy between interventions (S3F–S3H Table).

#### Probability ranking

The results showed that in terms of CTRS, pycnogenol (0.32), phosphatidylserine+omega-3 (0.47), and omega-3 (0.50) were the top three antioxidant drugs in improving attention; phosphatidylserine+omega-3 (0.26), MPH (0.29), and zinc (0.30) were the most effective in improving hyperactivity; zinc (0.34), MPH (0.36), and omega-3+6 (0.42) were the most effective in improving total score (S4F–S4H Table).

### ADHD Rating Scale-Parent (ADHD RS-Parent)

#### Evidence network

The evidence network of attention score included 11 antioxidant drugs in 21 studies, with 1,342 patients. The evidence network of hyperactivity score included 11 antioxidant drugs in 20 studies, with 1,207 patients. There are two evidence networks of total score: Network A and Network B. Network A included 7 antioxidant drugs in 13 studies, with 887 patients, while network B included 9 antioxidant drugs in 11 studies, with 580 patients (S3I–S3L Fig).

#### Heterogeneity test and network meta-analysis

Heterogeneity test was carried out on attention, hyperactivity and total score, and the results showed no heterogeneity among the studies (I^2^ < 50%) (S2H–S2K Fig). Therefore, the fixed-effects model was selected for analysis. The network meta-analysis showed no statistically significant differences in efficacy between interventions (S3I–S3L Table).

#### Probability ranking

The results showed that in terms of ADHD RS-Parent, the top three antioxidant drugs were phosphatidylserine (0.39), vitamin D+MPH (0.40), and vitamin D (0.43)for improving attention, with resveratrol+MPH (0.24), placebo+MPH (0.30), and ginkgo+MPH (0.31) for improving hyperactivity, phosphatidylserine (0.34), vitamin D (0.35), and omega-3 (0.49) for improving total score (network A), and zinc+MPH (0.35), sweet almond syrup+placebo (0.41), and resveratrol+ MPH (0.46) for improving total score (network B) (S4I–S4L Table).

### ADHD Rating Scale-Teacher (ADHD RS-Teacher)

#### Evidence network and network meta-analysis

The evidence network of attention score included 3 antioxidant drugs in 3 studies, with 167 patients. The evidence network of hyperactivity score included 3 antioxidant drugs in 3 studies, with 167 patients. Three evidence networks existed for the total score: Networks A, B, and C. Network A included 3 antioxidant drugs in 3 studies, with 187 patients; network B included 5 antioxidant drugs in 5 studies, with 263 patients; and the evidence network C included 3 antioxidant drugs in 3 studies, with 136 patients (S3M–S3Q Fig). The network meta-analysis of attention, hyperactivity, and overall scores showed no significant differences in efficacy between interventions (S3M–S3Q Table).

#### Probability ranking

Regarding ADHD RS-Teacher, the top three antioxidant drugs were pycnogenol (0.32), vitamin D (0.39), and omega-3 (0.63) for improving attention; vitamin D (0.31), pycnogenol (0.39), and omega-3 (0.62) for improving hyperactivity; vitamin D (0.18), omega-3+6 (0.43), and omega-3 (0.66) for improving total score (network A); zinc+MPH (0.29), resveratrol+MPH (0.45), and Acetyl-L-carnitine+MPH (0.47) in terms of total score (network B); MPH(0.38), zinc (0.44), quercetin (0.46) for improving total score (network C) (S4M–S4Q Table).

### Clinical global impressions scale (CGI)

#### Evidence network and network meta-analysis

The evidence network of CGI included 4 antioxidant drugs in 4 studies, with 331 patients (S3R Fig). The network meta-analysis showed no statistical differences in efficacy between interventions (S3R Table).

#### Probability ranking

In terms of CGI score, omega-3+6 (0.95) had the highest response rate (S4R Table).

### Continuous performance test (CPT)

#### Evidence network and network meta-analysis

The evidence network included 4 antioxidant drugs in 4 studies, with 230 patients (S3S Fig). The network meta-analysis showed no statistical differences in efficacy between interventions (S3S Table).

#### Probability ranking

In the CPT test, the most effective antioxidant drug for improving attention was omega-3+6 (0.42) (S4S Table).

### Quality evaluation of the included studies

**Fig 3:**
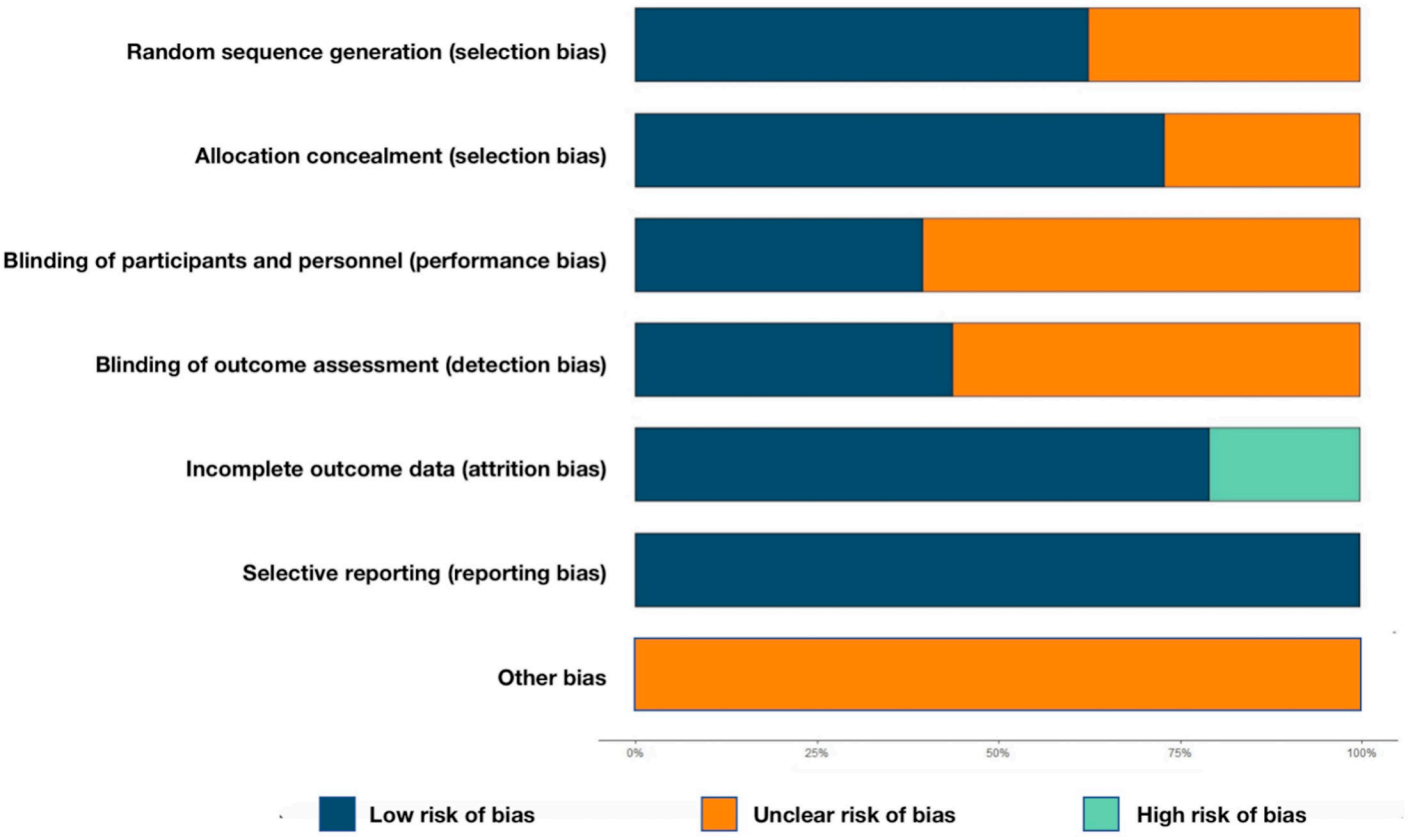
Quality evaluation of included studies.

The results of the risk of bias assessment are presented in Fig 3. Among the 48 included studies, 29 studies provided specific methods for random allocation [31–34, 36–39, 41, 45–63, 78], and were therefore rated as having a low risk of bias in random allocation. Most of these studies employed computer random sequence allocation. The remaining 19 studies only mentioned random allocation without specific method descriptions and were rated as having an unclear risk [35, 40, 42–44, 64–77]. Notably, none of the studies reported allocation concealment. Nineteen studies reported blinding of intervention for both subjects and medical staff, and they were rated as having a low risk of bias [31–33, 37, 39, 41, 50–54, 56–58, 62, 74, 76–78]. Twenty studies indicated blinding of outcome evaluators, and they were also rated as low-risk [31–34, 37–39, 41, 50–53, 55–57, 62, 74, 76–78]. The data in most studies were relatively complete. However, ten studies only reported the loss to follow-up without explaining the exact reasons for the losses [31, 32, 40, 46, 53, 54, 58, 65, 68, 72], and as a result, they were rated as having a high risk of bias in this regard. No selective reporting bias was identified, and all studies were rated as low-risk. The existence of other sources of bias remained unknown, as detailed in S5 Table.

## Discussion

The network meta-analysis of the 48 included studies revealed that seven antioxidant drugs ranked higher in terms of safety when compared to placebo and MPH. Furthermore, the best antioxidant drug in each rating scale demonstrated superior efficacy compared to placebo, and these results are clearly reflected in the probability rankings. There was no similar systematic review before. In pediatric clinical practice, drug treatment should be individualized based on specific symptoms in children with ADHD. The results of this systematic review are of great clinical and social significance, providing a reference for patients suffering from ADHD to select optimal drugs worldwide [79]. Most children with ADHD are found in families with low social status or low income [80]. However, central stimulants and behavioral training are costly, and the economic pressures may result in discontinuation of interventions, leading to rebound behavior problems. Parents may also neglect their child’s physical and mental health, and their children in the long run may develop mood disorders, language disorders, social disorders, resulting in a poor prognosis [81, 82]. Evidence shows that oxidative stress may be a major pathophysiological factor for ADHD [83]. Persistent psychological problems increase ROS levels, enhance oxidative stress and decrease brain antioxidant capacity, contributing largely to neurological damage [84] and a series of functional disorders [85]. Therefore, seeking safer, more economical, and more effective antioxidant drugs is urgently needed. Various antioxidant drugs with inconsistent mechanisms and mutual interaction play an antioxidant role in vivo through different pathways.

### Regulate intercellular signal transduction

Based on the NMA results, unsaturated fatty acid is the best option for the improvement of CPRS, CGI, and CPT. Unsaturated fatty acids interact with membrane phospholipids to form structural modifiers, which increase membrane flow and permeability [86], help regulate NRF1/HMOX1 signaling pathway [87], reduce oxidative stress in vivo, and relieve anxiety symptoms [88]. Similarly, resveratrol easily crosses the blood-brain barrier due to its lipophilic structure, participates in astrocyte proliferation and activation, maintains T-lymphocyte mediated adaptive immunity, and exhibits the anti-inflammation effect, and reduces stimulation of the central nervous system [89]. This may be the reason why it ranks high in improving ADHD RS-Parent’s hyperactivity score, and long-term use may also reduce cognitive impairment [90]. These two drugs modulate the phenotype and function of M1 and M2 macrophages, indirectly enhancing the antioxidant barrier [91]. In addition, pycnogenol and ginkgo biloba both inhibit NF-κB signaling [92, 93], thereby reducing ROS-mediated neuroinflammatory responses and preventing the exacerbation of behavioral problems [94].

### Regulate the expression level of genes or proteins

Our results showed that pycnogenol and zinc were the best options for ADHD patients in terms of CPRS, CTRS, and ADHD RS-Teacher. Aydın et al. found that the level of 8-hydroxy-2’-deoxyguanosine, which is the main marker of damage of purine, decreased in vivo after pycnogenol supplement [95], indicating that it is helpful to repair oxidative damage. Kim et al. also found that pycnogenol can repair hydroxyl radical-induced DNA breaks, reducing cell death and brain dysfunction [96]. Zinc can reduce the activation of NF-κB and its target gene, and increase the gene expression of A20 and PPAR-α [97], delay the oxidation process and maintain the working memory ability [98]. In addition, activated vitamin D can promote the expression of Klotho gene [99], regulate the formation of related cellular signaling systems by antioxidants [100], block the uptake of active oxygen by brain neurons, and reduce neurotoxic reactions [101]. All this may explain the reason why these drugs are effective in improving ADHD assessed by ADHD RS-Teacher and CPRS.

### Regulate the activity of enzymes

Antioxidant drugs can regulate enzyme activity directly or indirectly to maintain oxidation-antioxidant balance. Our results showed that phosphatidylserine was the most effective drug to improve the attention of ADHD RS-Parent. Exogenous phosphatidylserine supplementation can enhance the activity of superoxide dismutase in the brain and directly participate in reducing the reaction of oxidative stress products [102]. Besides, it can also play this role when combined with unsaturated fatty acids [103]. Unsaturated fatty acids are indirectly involved in the metabolism of 5-hydroxytryptamine (5-HT) by regulating enzyme activity [104], inhibiting impulsive or self-destructive behavior [105]. In addition, vitamin D can promote the production of tyrosine hydroxylase and increase the concentration of dopamine in synaptic space [106], reduce the excitability of brain neurons [107], and improve patients’ self-control ability. Almonds can also indirectly maintain acetylcholine concentrations in synaptic spaces by regulating cholinesterase activity [108], improving executive function.

### Regulate mitochondrial function

Mitochondria with stable structure and function play a pivotal role in antioxidant effect [109]. Overproduction of ROS can damage mitochondrial integrity, and activated vitamin D interacts with vitamin D receptors to counter such damage and stabilize metabolism in brain nerve cells [110]. Vanani and Wang showed that quercetin could reverse the oxidative stress process in hypertensive mice, alleviate the ultrastructural damage of mitochondria, and maintain normal learning or working cycles [111, 112]. Resveratrol can increase the number of mitochondria in mammalian cells by triggering Mitochondrial Biogenesis, improve the energy metabolism of the brain, and assist in antioxidation [113].

### Regulate neurotrophic factors

Animal experiments have shown that acetyl-L-carnitine and folic acid can regulate the levels of neurotrophic factors, such as enhancing the expression of BDNF in the prefrontal cortex and hippocampus, promoting protein synthesis and nourishing neurons, and exerting antidepressant effects through trophic nerves [114, 115]. Zinc can also help people with mood disorders in a similar way [116].

In terms of safety, unsaturated fatty acids were found to have the lowest incidence of adverse reactions among the 12 drugs. It is highly accepted, which may be related to parental preference or family diet [117]. We also summarized the adverse reactions reported in various studies (S6 and S7 Tables). It should be noted that we classified some special adverse reactions as Others. For example, some studies used multiple evaluation systems and reported even the side effects unrelated to drugs. There are also some drug-specific adverse reactions. For example, Zn has a bad taste, which is inevitable.

A total of 1,194 adverse reactions were reported in the included studies. Decreased appetite had the highest incidence (18.93%), with 198 cases reported in studies related to MPH use. Only 7 cases were reported during the use of herbal medicines, 6 of which were caused by drugs combined with MPH. The association between MPH and gastrointestinal symptoms has been demonstrated [118], so herbal antioxidants alone may be safer than in combination with central stimulants in terms of appetite loss. Headache was reported in 167 cases (13.99%), but it was not found in patients treated with Vitamin D, Folic, Zinc, Pycnogenol and almond. It is noteworthy that the side effects of anxiety/nervousness (12.06%) were reported in 65 cases on MPH alone, and in 23 cases on MPH+placebo. A clinical study has confirmed that MPH can cause similar adverse reactions [25]. A total of 136 cases (11.39%) of abdominal pain were reported, 73 of which were associated with MPH use. The antioxidant ALC was the most frequently reported. Insomnia was reported in 112 cases (9.38%) and drowsiness in 34 cases. The above results are only based on our simple statistical analysis of the side effects in the included studies and need to be carefully interpreted (S6 and S7 Tables).

According to DSM-5 guidelines, the combination of antioxidant drugs and non-pharmacological intervention is also worthy of attention [28]. Several non-pharmacological studies provide considerable evidence to support the treatment of ADHD with CBT, mindfulness, and neurofeedback [119]. CBT can effectively improve behavioral problems and cognitive abilities in ADHD patients [118] and ADHD children with emotional problems [120]. Mindfulness is more suitable for patients with attention deficit or hyperactivity disorder [121]. These treatments are based on traditional or emerging psychological theories, such as cognitive behavioral theory and the human birth theory [16]. They believe that neurodevelopmental disorders may be related to poor parenting patterns in early growth and development. Discordant parent-child relationships cannot foster positive psychology and family well-being, and have long-term impacts on academic, social, and emotional functioning in children with ADHD [122]. In fact, tense family relationships can seriously damage their mental and physical health, such as growth, metabolism, and oxidative stress [123]. Antioxidant therapy, supplemented by psychotherapy, can reduce oxidative stress levels, improve self-regulation, and alleviate emotional problems. A previous study has pointed out that antioxidant drugs are effective as adjuvant therapy for nervous system diseases [124]. However, there are few clinical studies on antioxidants combined with psychological intervention in the treatment of children and adolescents with ADHD. More clinical evidence is urgently needed to verify this finding.

### Limitations and shortcomings

There are several limitations in this study. First, the implementation of randomization and allocation concealment was not described in some studies. Second, variability in the number of studies included for different interventions may introduce uncertainties into the results, potentially affecting the robustness of the findings. Third, the diversity in drug therapy regimens across different studies, as well as the absence of direct comparisons between certain intervention measures, can make it challenging to draw clear conclusions. Fourth, the substantial variation in the duration of different studies may influence the results. Fifth, the safety analysis of the included studies primarily focused on the incidence of adverse events. Due to these limitations, the ranking results should be interpreted with caution. More high-quality, large-sample, multicenter double-blind randomized controlled trials are required for further verification.

## Future directions and conclusion

It is worth noting that a single pathogenesis theory may limit the treatment of ADHD [13]. The occurrence and manifestations of ADHD are complex and multidimensional, and the ADHD symptoms cannot be explained from a single aspect [125]. This suggests that other etiological theories may provide novel insights into the treatment of ADHD. For example, antioxidants can be added to existing treatment modalities. Antioxidant therapy combined with psychotherapy may also be a useful treatment strategy, and no evidence of psychotherapy causing severe non-response has been found in clinical practice [126]. Hence, it may be a new treatment option for ADHD or other neurological disorders in their early stages, although the relationship between ADHD and oxidative stress in children is still understudied.

In conclusion, through the application of network meta-analysis, this study conducted a pioneering comparison of the safety and efficacy of various antioxidant drugs. The findings revealed distinct rankings for safety and efficacy among the 12 antioxidant drugs examined. Consequently, this study offers valuable high-level, evidence-based medical insights for the selection of antioxidant drugs for children with ADHD. In pediatric clinical practice, these findings can inform individualized treatment decisions. However, due to the low methodological quality of the included studies, the probability ranking cannot fully explain the clinical efficacy, and the results should be interpreted cautiously. More high-quality studies are still needed to verify our findings.

## Supporting information

S1 Checklist PRISMA NMA checklist for the present systematic review. (DOCX)

S1 Table Search terms and history. (DOCX)

S2 Table Baseline characteristics of included studies. (DOCX)

S3 Table Network meta analysis. (DOCX)

S4 Table Probability ranking of SUCRA value. (DOCX)

S5 Table Risk of bias for included studies. (DOCX)

S6 Table Number and severity of side effect event per study. (DOCX)

S7 Table Symptom and number of side effect event per study. (DOCX)

S8 Table Definition and interpretation of outcome indicators and statistical analysis. (DOCX)

S1 Fig Consistency test. (DOCX)

S2 Fig Heterogeneity test. (DOCX)

S3 Fig Network geometry. (DOCX)

S1 File Methods clarifications from the protocol. (DOCX)
