# Supplementary material for: Safety and efficacy of antioxidant therapy in children and adolescents with attention deficit hyperactivity disorder: A systematic review and network meta-analysis
Source: PLoS One. 2024 Mar 28;19(3):e0296926. doi: 10.1371/journal.pone.0296926 (PMC10977718; doi:10.1371/journal.pone.0296926)
Supplement: S1 Table — (DOCX) [file pone.0296926.s002.docx]

Supplementary Material

## S2 Table. Search terms and history.

**Pubmed**

| **Search** | **Query** | **Items found** |
| --- | --- | --- |
| #1 | **Search** Attention Deficit Disorder with Hyperactivity[MeSH Terms] | 33832 |
| #2 | **Search**"ADDH" OR "ADHD" OR "attention deficit" OR "attention deficit and disruptive behavior disorders" OR "attention deficit and disruptive behaviour disorders" OR "Attention Deficit Disorder" OR "Attention Deficit Disorder with Hyperactivity" OR "Attention Deficit Disorders" OR "Attention Deficit Disorders with Hyperactivity" OR "Attention Deficit Hyperactivity Disorder" OR "Attention Deficit Hyperactivity Disorders" OR "Hyperkinetic Syndrome" OR "Minimal Brain Dysfunction" | 50138 |
| #3 | **Search** Child[MeSH Terms] | 2115494 |
| #4 | **Search** children | 3050632 |
| #5 | **Search** Adolescent[MeSH Terms] | 2196548 |
| #6 | **Search** "Adolescence" OR "adolescent" OR "Adolescents" OR "Female Adolescent" OR "Female Adolescents" OR "Male Adolescent" OR "Male Adolescents" OR "Teen" OR "teenager" OR "Teenagers" OR "Teens" OR "Youth" OR "Youths" | 2360380 |
| #7 | **Search** Antioxidants[MeSH Terms] | 161511 |
| #8 | **Search** "Anti Oxidant" OR "Anti Oxidant Effect" OR "Anti Oxidant Effects" OR "Anti Oxidants" OR "antioxidant" OR "Antioxidant Activity" OR "antioxidant agent" OR "Antioxidant Effect" OR "Antioxidant Effects" OR "antioxidant nutrient" OR "antioxidants" OR "antioxidation agent" OR "antioxidation product" OR "antioxidative" OR "antoxidant" OR "Endogenous Antioxidant" OR "Endogenous Antioxidants" OR "phenolic antioxidant" | 689774 |
| #9 | **Search** Fatty Acids, Unsaturated[MeSH Terms] | 239311 |
| #10 | **Search** "alkenyl fatty acid" OR "Polyunsaturated Fatty Acid" OR "UFA" OR "unsaturated fat" OR "unsaturated fatty acid" OR "unsaturated lipid" | 14559 |
| #11 | **Search** Zinc[MeSH Terms] | 66067 |
| #12 | **Search** Zn | 94095 |
| #13 | **Search** pycnogenols [Supplementary Concept] | 401 |
| #14 | **Search** "French maritime pine bark extract" OR "leucocianidol" OR "leucocyanidin" OR "maritime pine bark extract" OR "Pinus pinaster bark extract" OR "Pycnogenol" OR "pygnoforton" | 545 |
| #15 | **Search** vitamin[MeSH Terms] | 42901 |
| #16 | **Search** "vitamins" OR "vitamin" | 456047 |
| #17 | **Search** Quercetin[MeSH Terms] | 11565 |
| #18 | **Search** "Dikvertin" OR "flavin" OR "hippuroflavin" OR "meletin" OR "quercetol" OR "quercitin" OR "quertine" | 16147 |
| #19 | **Search** Ginkgo biloba[MeSH Terms] | 3484 |
| #20 | **Search** "Gingko" OR "Ginkgo" OR "Ginkgo bilobas" OR "Ginkgophyta" OR "Ginko" OR "Maidenhair Tree" | 5717 |
| #21 | **Search** #1 OR #2 | 50139 |
| #22 | **Search** #3 OR #4 OR #5 OR #6 | 4230498 |
| #23 | **Search** #7 OR #8 OR #9 OR #10 OR #11 OR #12 OR #13 OR #14 OR #15 OR #16 OR #17 OR #18 OR #19 OR #20 | 1395846 |
| #24 | **Search** #21 AND #22 AND #23 | 664 |

**Embase**

| **Search** | **Query** | **Items found** |
| --- | --- | --- |
| #1 | **Search** 'attention deficit hyperactivity disorder'/exp OR 'attention deficit hyperactivity disorder' | 78275 |
| #2 | **Search**'addh' OR 'adhd' OR 'attention deficit' OR 'attention deficit and disruptive behavior disorders' OR 'attention deficit and disruptive behaviour disorders' OR 'attention deficit disorder' OR 'attention deficit disorder with hyperactivity' OR 'attention deficit disorders' OR 'attention deficit disorders with hyperactivity' OR 'attention deficit hyperactivity disorder' OR 'attention deficit hyperactivity disorders' OR 'hyperkinetic syndrome' OR 'minimal brain dysfunction' | 82954 |
| #3 | **Search** 'child'/exp | 3268454 |
| #4 | **Search** 'children' | 2295100 |
| #5 | **Search** 'adolescent'/exp | 1869499 |
| #6 | **Search** 'adolescence' OR 'adolescent' OR 'adolescents' OR 'female adolescent' OR 'female adolescents' OR 'male adolescent' OR 'male adolescents' OR 'teen' OR 'teenager' OR 'teenagers' OR 'teens' OR 'youth' OR 'youths' | 2118594 |
| #7 | **Search** 'antioxidant'/exp | 303397 |
| #8 | **Search** 'anti oxidant' OR 'anti oxidant effect' OR 'anti oxidant effects' OR 'anti oxidants' OR 'antioxidant' OR 'antioxidant activity' OR 'antioxidant agent' OR 'antioxidant effect' OR 'antioxidant effects' OR 'antioxidant nutrient' OR 'antioxidants' OR 'antioxidation agent' OR 'antioxidation product' OR 'antioxidative' OR 'antoxidant' OR 'endogenous antioxidant' OR 'endogenous antioxidants' OR 'phenolic antioxidant' | 419140 |
| #9 | **Search** 'unsaturated fatty acid'/exp | 177492 |
| #10 | **Search** 'alkenyl fatty acid' OR 'polyunsaturated fatty acid' OR 'ufa' OR 'unsaturated fat' OR 'unsaturated fatty acid' OR 'unsaturated lipid' | 51716 |
| #11 | **Search** 'zinc'/exp | 136645 |
| #12 | **Search** 'zn' | 106622 |
| #13 | **Search** 'pycnogenol'/exp | 880 |
| #14 | **Search** 'french maritime pine bark extract' OR 'leucocianidol' OR 'leucocyanidin' OR 'maritime pine bark extract' OR 'pinus pinaster bark extract' OR 'pycnogenol' OR 'pygnoforton' | 1045 |
| #15 | **Search** 'vitamin'/exp | 772767 |
| #16 | **Search** 'vitamins' OR 'vitamin' | 424760 |
| #17 | **Search** 'quercetin'/exp | 40170 |
| #18 | **Search** 'dikvertin' OR 'flavin' OR 'hippuroflavin' OR 'meletin' OR 'quercetol' OR 'quercitin' OR 'quertine' | 33057 |
| #19 | **Search** 'ginkgo biloba'/exp | 5294 |
| #20 | **Search** 'gingko' OR 'ginkgo' OR 'ginkgo bilobas' OR 'ginkgophyta' OR 'ginko' OR 'maidenhair tree' | 12546 |
| #21 | **Search** #1 OR #2 | 82954 |
| #22 | **Search** #3 OR #4 OR #5 OR #6 | 5010767 |
| #23 | **Search** #7 OR #8 OR #9 OR #10 OR #11 OR #12 OR #13 OR #14 OR #15 OR #16 OR #17 OR #18 OR #19 OR #20 | 1650923 |
| #24 | **Search** #21 AND #22 AND #23 | 1964 |

**Cochrane Library**

| **Search** | **Query** | **Items found** |
| --- | --- | --- |
| #1 | **Search** MeSH descriptor: [Attention Deficit Disorder with Hyperactivity] | 3089 |
| #2 | **Search**  ('ADDH' OR 'ADHD' OR 'attention deficit' OR 'attention deficit and disruptive behavior disorders' OR 'attention deficit and disruptive behaviour disorders' OR 'Attention Deficit Disorder' OR 'Attention Deficit Disorder with Hyperactivity' OR 'Attention Deficit Disorders' OR 'Attention Deficit Disorders with Hyperactivity' OR 'Attention Deficit Hyperactivity Disorder' OR 'Attention Deficit Hyperactivity Disorders' OR 'Hyperkinetic Syndrome' OR 'Minimal Brain Dysfunction') | 8526 |
| #3 | **Search** MeSH descriptor: [Child] | 62328 |
| #4 | **Search** ('children') | 190770 |
| #5 | **Search** MeSH descriptor: [Adolescent] | 110852 |
| #6 | **Search** ('Adolescence' OR 'adolescent' OR 'Adolescents' OR 'Female Adolescent' OR 'Female Adolescents' OR 'Male Adolescent' OR 'Male Adolescents' OR 'Teen' OR 'teenager' OR 'Teenagers' OR 'Teens' OR 'Youth' OR 'Youths') | 158904 |
| #7 | **Search** MeSH descriptor: [Antioxidants] | 5249 |
| #8 | **Search** ('Anti Oxidant' OR 'Anti Oxidant Effect' OR 'Anti Oxidant Effects' OR 'Anti Oxidants' OR 'antioxidant' OR 'Antioxidant Activity' OR 'antioxidant agent' OR 'Antioxidant Effect' OR 'Antioxidant Effects' OR 'antioxidant nutrient' OR 'antioxidants' OR 'antioxidation agent' OR 'antioxidation product' OR 'antioxidative' OR 'antoxidant' OR 'Endogenous Antioxidant' OR 'Endogenous Antioxidants' OR 'phenolic antioxidant') | 16329 |
| #9 | **Search** MeSH descriptor: [Fatty Acids, Unsaturated] | 13401 |
| #10 | **Search**('alkenyl fatty acid' OR 'Polyunsaturated Fatty Acid' OR 'UFA' OR 'unsaturated fat' OR 'unsaturated fatty acid' OR 'unsaturated lipid') | 4212 |
| #11 | **Search** MeSH descriptor: [Zinc] | 1743 |
| #12 | **Search** ('Zn') | 1548 |
| #13 | **Search** ('French maritime pine bark extract' OR 'leucocianidol' OR 'leucocyanidin' OR 'maritime pine bark extract' OR 'Pinus pinaster bark extract' OR 'Pycnogenol' OR 'pygnoforton') | 167 |
| #14 | **Search** MeSH descriptor: [Vitamins] | 5299 |
| #15 | **Search** ('vitamins' OR 'vitamin') | 36376 |
| #16 | **Search** MeSH descriptor: [Quercetin] | 216 |
| #17 | **Search** ('Dikvertin' OR 'flavin' OR 'hippuroflavin' OR 'meletin' OR 'quercetol' OR 'quercitin' OR 'quertine') | 264 |
| #18 | **Search** MeSH descriptor: [Ginkgo biloba] | 309 |
| #19 | **Search** ('Gingko' OR 'Ginkgo' OR 'Ginkgo bilobas' OR 'Ginkgophyta' OR 'Ginko' OR 'Maidenhair Tree') | 1289 |
| #20 | **Search** (#1 OR #2) | 8526 |
| #21 | **Search** (#3 OR #4 OR #5 OR #6) | 288536 |
| #22 | **Search** (#7 OR #8 OR #9 OR #10 OR #11 OR #12 OR #13 OR #14 OR #15 OR #16 OR #17 OR #18 OR #19) | 66685 |
| #23 | **Search** (#20 AND #21 AND #22) | 311 |
