# Supplementary material for: Safety and efficacy of antioxidant therapy in children and adolescents with attention deficit hyperactivity disorder: A systematic review and network meta-analysis
Source: PLoS One. 2024 Mar 28;19(3):e0296926. doi: 10.1371/journal.pone.0296926 (PMC10977718; doi:10.1371/journal.pone.0296926)
Supplement: S2 Table — (DOCX) [file pone.0296926.s003.docx]

Supplementary Material

## S3 Table. Baseline characteristics of included studies.

| **Study, Year**  **Country**  **Study design** | **Interventions** | **Sample size** | **Details of interventions** | **Outcomes** | **Diagnose** | **Period**  **weeks** |
| --- | --- | --- | --- | --- | --- | --- |
| Rafeiy Torghabeh et al, 2020  Iran  RCT | Resveratrol + Methylphenidate (MPH)  Placebo + Methylphenidate (MPH) | 30  30 | Resveratrol 500mg/day  + MPH 0.3-1.5 mg/kg/day  Starch 500 mg/day  + MPH 0.3-1.5 mg/kg/day | ADHD RS-Parent  ADHD RS-Teacher  Adverse events | DSM-5 | 8w |
| Motaharifard et al, 2019  Iran  RCT | Sweet almond syrup + Placebo  Methylphenidate (MPH) + Placebo | 25  25 | Sweet almond syrup 15 cc/day  + Placebo 1 mg/kg/day  MPH 1 mg/kg/day + Placebo 15 cc/day | ADHD RS-Parent  ADHD RS-Teacher  Adverse events | DSM-5 | 8w |
| Akhondzadeh et al, 2005  Iran  RCT | Passion flower  Pethylphenidate (MPH) | 17  17 | Passion flower 0.08 mg/kg/day  MPH 2mg/kg/day | ADHD RS-Parent  ADHD RS-Teacher  Adverse events | DSM-IV | 8w |
| Weber et al, 2008  America  RCT | Hypericum perforatum  Placebo | 27  27 | Hypericum perforatum 900mg/day  placebo 900mg/day | ADHD RS-Parent  Adverse events  Clinical Global Impression | DSM-IV | 8w |
| Ghanizadeh et al,  2013  Iran  RCT | Methylphenidate (MPH) + Folic  Methylphenidate (MPH) +Placebo | 23  26 | MPH <24kg 10 mg/day; >24kg 20mg/day  + Folic 5mg/day  MPH <24kg 10 mg/day; >24kg 20mg/day  + Placebo | ADHD RS-Parent  Adverse events | DSM-IV | 8w |
| Riahi et al, 2018  Iran  RCT | Methylphenidate (MPH) + Folic  Methylphenidate (MPH) + placebo | 31  29 | MPH 1mg/kg/day + Folic acid 5mg/day  MPH 1mg/kg/day + placebo 5mg/day | Conners’ Parent RS | DSM-IV | 8w |
| Salehi et al, 2009  Iran  RCT | Ginkgo  Methylphenidate (MPH) | 25  25 | Ginkgo <30kg 80mg/day; >30kg 120mg/day  MPH <30kg 20mg/day; >30kg 30 mg/day | ADHD RS-Parent  ADHD RS-Teacher  Adverse events | DSM-IV | 6w |
| Shakibaei et al,  2015  Iran  RCT | Ginkgo + Methylphenidate (MPH)  Placebo + Methylphenidate (MPH) | 31  29 | Ginkgo 80-120mg/day + MPH 20-30mg/day  Starch and lactose 80-120mg/day  + MPH 20-30mg/day | ADHD RS-Parent  ADHD RS-Teacher  Adverse events | DSM-IV | 6w |
| Abbasi et al, 2013  Iran  RCT | Acetyl-L-carnitine (ALC)  + Methylphenidate (MPH)  Placebo + Methylphenidate (MPH) | 20  20 | ALC 13.5-30 kg 1g/day; 30-50 kg 2g/day ; >50kg 3g/day + MPH  MPH <30kg 20mg/day; >30kg 30mg/day  + Placebo | ADHD RS-Parent  ADHD RS-Teacher  Adverse events | DSM-IV | 6w |
| Arnold et al, 2007  American  RCT | Placebo  Acetyl-L-carnitine (ALC) | 59  53 | Placebo  ALC 13.5-30 kg 1g/day; 30-50 kg 2g /day; >50kg 3g/day | Conners’ Parent RS  Conners’ Teacher RS  Adverse events  Clinical Global Impression | DSM-IV | 16w |
| Akhondzadeh et  al, 2004  Iran  RCT | Methylphenidate (MPH) + Zinc  Methylphenidate (MPH) + placebo | 22  22 | MPH 2mg/kg/day + Zinc 55 mg/day  MPH 2mg/kg/day + Sucrose 55 mg/day | ADHD RS-Parent  ADHD RS-Teacher  Adverse events | DSM-IV | 6w |
| Arnold et al, 2011  America  RCT | Zinc  Placebo | 28  24 | Zinc 15-30mg/day  Placebo | ADHD RS-Parent  ADHD RS-Teacher  Conners’ Parent RS  Conners’ Teacher RS | DSM-IV | 8w |
| Bilici et al, 2004  Turkey  RCT | Zinc sulfate  Placebo | 202  198 | Zinc sulfate 150mg/day  Placebo | Conners Teacher RS  Adverse events | DSM-IV | 12w |
| Noorazar et al,  2020  Iran  RCT | Methylphenidate (MPH) + Zinc  Methylphenidate (MPH) + Placebo | 30  30 | MPH 0.5–1mg/kg/day  + Zinc sulfate syrup 10 cc/day  MPH 0.5–1mg/kg/day + Placebo | Conners’ Parent RS | Clinician | 6w |
| Hsu et al, 2021  China  RCT | Pycnogenol  Placebo | 7  13 | Pycnogenol ≤50kg 25mg/day; >50 kg 50mg/day  Placebo | ADHD RS-Parent  ADHD RS-Teacher  Adverse events  Continuous Performance Test | DSM-IV | 4w |
| Trebaticka et al,  2006  Slovakia Republic  RCT | Pycnogenol  Placebo | 44  17 | Pycnogenol 1mg/kg  Lactose 58mg/day and cellulose 65mg/day | Conners’ Parent RS  Conners’ Teacher RS  Adverse events | ICD-10 | 4w |
| Manor et al, 2012  Israel  RCT | omega-3 + Phosphatidylserine (PS)  Placebo | 100  47 | PS 300mg/day + EPA 80mg + DHA 40mg  Encapsulated cellulose | Conners’ Parent RS  Conners’ Teacher RS  Adverse events | DSM-IV | 15w |
| Hirayama et al,  2014  Japan  RCT | Phosphatidylserine (PS)  Placebo | 19  17 | PS ( cocoa-flavored chews contained 200mg/day )  cocoa-flavored chews | ADHD RS-Parent  Adverse events | Clinician | 8w |
| Vaisman et al,  2008  Israel  RCT | omega-3 +Phosphatidylserine (PS)  omega-3  Placebo | 18  21  21 | omega-3 ( EPA 156mg +DHA 95mg )  + PS 300mg/day  Fish oil ( EPA 153mg + DHA 96mg )  Placebo | Conners’ Parent RS  Adverse events | _ | 12w |
| Dehbokri et al,  2018  Iran  RCT | Methylphenidate (MPH) + VitaminD  Methylphenidate (MPH) + Placebo | 51  45 | MPH + Vitamin D 50000ul/day  MPH + Placebo | Conners’ Parent RS  Adverse events | Clinician | 6w |
| Elshorbagy et al,  2018  Egypt  Prospective study | Methylphenidate (MPH)+ Vitamin D  Methylphenidate (MPH) + Placebo | 20  30 | MPH 0.9-3mg/kg/d + Vitamin D 3000ul/day  MPH 0.9-3mg/kg/d + Placebo | Adverse events | DSM-IV | 12w |
| Mohammadpour et al, 2016  Iran  RCT | Methylphenidate (MPH) + VitaminD  Methylphenidate (MPH) + Placebo | 31  31 | MPH 0.9-3mg/kg/d + Vitamin D 2000 IU/day  MPH 0.9-3mg/kg/d + Starch | Conners’ Parent RS  ADHD RS-Parent  Adverse events | DSM-IV | 8w |
| Naeini et al, 2019  Iran  RCT | vitamin D  Placebo | 36  35 | vitamin D3 tablet 1000 IU/day  Placebo | Conners’ Parent RS  Continuous Performance Test | _ | 12w |
| Rahmani et al,  2022  Iran  RCT | Vitamin D  Placebo | 26  26 | Vitamin D3 pearl 50 000 IU/week  Placebo | ADHD RS-Parent  ADHD RS-Teacher  Adverse events | DSM-5 | 12w |
| Hemamy et al,  2020  Iran  RCT | Vitamin D  Placebo | 33  33 | Vitamin D 50,000 IU/week  Placebo ( edible paraffin oil + microcrystalline cellulose and stearic acid ) | Conners’ Parent RS | DSM-IV | 8w |
| Assareh et al,  2012  Iran  RCT | Methylphenidate (MPH) + omega-3+6  Methylphenidate (MPH) + Placebo | 20  20 | MPH 0.3mg/kg/day increased to 1mg/kg/day  + omega-6 capsules ( DHA 241mg + EPA33mg +omega-6 180mg )/day  MPH 0.3mg/kg/day increased to 1mg/kg/day  + Placebo | ADHD RS-Parent  Adverse events | DSM-IV | 10w |
| Barragán et al,  2014  Mexico  Prospective study | Methylphenidate (MPH)  omega-3+6  Methylphenidate (MPH) + omega-3+6. | 30  30  30 | MPH 0.3mg/kg/day increased to 1mg/kg/day  omega-3+6 ( EPA 558mg +DHA 174mg +GLA 60mg )/day  MPH 0.3mg/kg/day increased to 1mg/kg/day  + omega-3+6  ( EPA 558mg +DHA 174mg+GLA 60mg )/day | ADHD RS-Parent  Adverse events  Clinical Global Impression | DSM-IV | 24w |
| Carucci et al,  2021  Italy  RCT | omega-3+6  Placebo | 67  68 | omega-3+6  ( EPA 558mg+DHA 174mg+GLA 60mg )/day  Placebo | ADHD RS-Parent  Conners’ Parent RS  Conners’ Teacher RS  Adverse events | DSM-IV | 24w |
| Döpfner et al,  2019  Germany  RCT | omega-3+6  Placebo | 20  20 | omega-3+6  ( EPA 744mg +DHA 232mg +GLA 80mg )/day  Placebo | ADHD RS-Parent | DSM-IV  ICD-10 | 16w |
| Johnson et al,  2012  Sweden  RCT | omega-3+6  Placebo | 37  38 | omega-3+6  ( EPA 558mg+DHA 174mg+ GLA 60mg  +Vitamin E10.8mg )/day  Placebo ( olive oil ) | ADHD RS-Parent  Adverse events  Clinical Global Impression | DSM-IV | 12w |
| Stevens et al,  2003  Indiana  RCT | omega-3+6  Placebo | 25  22 | omega-3+6  ( DHA 480mg+EPA 80mg+AA 40mg  +GLA 96mg +Vitamin E 24mg )/day  Placebo ( 6.4g olive oil/day ) | Continuous Performance Test | DSM-IV | 16w |
| Matsudaira et al,  2015  United Kingdom  RCT | omega-3+6  Placebo | 38  38 | omega-3+6  ( EPA 558mg+DHA 174mg+GLA 60mg  +Vitamin E 9.6 mg )/day  Placebo | Adverse events | DSM-IV | 12w |
| Cornu et al, 2017  France  RCT | omega-3  Placebo | 71  77 | omega-3  ( 6–8 years, EPA 336mg+DHA 84mg;  9–11 years, EPA 504mg + DHA 126mg;  12–15 years,EPA 672mg+DHA 168mg )/day  Placebo capsules ( olive oil ) | ADHD RS-Parent  Conners’ Parent RS  Adverse events | DSM-IV | 12w |
| Behdani et al,  2013  Iran  RCT | Methylphenidate (MPH) + omega-3  Methylphenidate (MPH) + Placebo | 36  33 | MPH 1mg/kg/day + omega-3  ( DHA 480mg+EPA 720 mg )/day  MPH 1mg/kg/day+Placebo | ADHD RS-Parent  ADHD RS-Teacher | DSM-IV | 8w |
| Bélanger, 2009  Canada  RCT | omega-3  omega-6 | 13  13 | omega-3  ( 16-25 kg EPA500mg ;26-35kg EPA 750mg  ;>36kg EPA 1000mg )/day  omega-6  ( 500mg sunflower oil: 70%linoleic acid + 20% oleic acid + 5% palmitic + 5% stearic acid + vitamin E ) | Conners’ Parent RS  Adverse events | DSM-IV | 8w |
| Chang et al, 2019  China  RCT | omega-3  placebo | 48  44 | omega-3 ( EPA 1.2g/day )  Placebo ( Soybean oil 1.2g/day ) | Adverse events  Continuous Performance Test | DSM-5 | 12w |
| Crippa et al, 2018  Italy  RCT | omega-3  Placebo | 25  25 | omega-3 ( DHA 500m/day )  Placebo  ( wheat germ oil 500mg+a low concentration of Vitamin E ) | ADHD RS-Parent  Conners’ Parent RS  Adverse events | DSM-IV | 24w |
| Kean et al, 2016  Australia  RCT | omega-3  Placebo | 54  58 | PCSO-524® capsule  ≤45 kg three capsules; >45 kg four capsules  ( EPA 7.3mg+DHA 5.5mg+natural mono-unsaturated olive oil 100mg+VE )/capsule  Placebo capsule  ≤45 kg three capsules; >45kg four capsules ( 35.5mg olive oil+112mg lecithin+12mg coconut oil+0.5mg beta-carotene )/capsule | Conners’ Parent RS | DSM-IV | 14w |
| Dubnov-Raz et al, 2014 Israel  RCT | omega-3  Placebo | 9  8 | omega-3  ( 50-54%ALA+20-23% oleic acid+16-18% linoleic acid+6% palmitic acid+2-3% stearic acid )  Placebo 2g/day | Conners’ Parent RS  Conners’ Teacher RS | DSM-IV | 8w |
| Gustafsson et al,  2010 Sweden  RCT | omega-3  Placebo | 46  46 | omega-3  ( EPA 500mg+DHA 2.7mg+vitamin E10mg )/day  Placebo  ( rape seed oil+medium-chain triglycerides ) | Conners’ Parent RS  Conners’ Teacher RS  Adverse events | DSM-IV | 15w |
| Hariri M et al,  2012  Iran  RCT | omega-3  Placebo | 53  50 | omega-3  ( EPA 635mg +DHA 195mg+ other omega-3 100mg )  Placebo  ( olive oil 900mg/day ) | Conners’ Parent RS | DSM-IV | 8w |
| Milte et al, 2011  Australia  RCT | omega-3  omega-6 | 58  29 | omega-3  ( EPA 1109mg+DHA 108mg or  EPA 264mg+DHA 1032mg )/day  omega-6 ( Linoleic Acid 1467mg/d ) | Adverse events | _ | 16w |
| Moghaddam et al,  2017 Iran  RCT | Methylphenidate (MPH) + omega-3  Methylphenidate (MPH) + Placebo | 20  20 | MPH 0.3mg/kg/day increased to 1mg/kg/day  +omega-3 ( EPA 180mg+DHA 120mg )/day  MPH 0.3mg/kg/day increased to 1mg/kg/day +Placebo | ADHD RS-Parent  Adverse events | Clinician | 8w |
| Mohammadzadeh et al, 2019  Iran  RCT | Methylphenidate (MPH) + omega-3  Methylphenidate (MPH) + Placebo | 33  33 | MPH  ( 10mg/day in the first week, 20mg/day from the second week )  +omega-3  ( EPA 18 mg+DHA 120mg)/day in the first week，EPA 360mg+ DHA240mg)/day from the second week )  MPH+Placebo ( olive oil )  similarity as the treatment group | ADHD RS-Parent  Adverse events | DSM-IV | 8w |
| Raz et al , 2009  Israel  RCT | omega-3  Placebo | 32  31 | Omega-3  ( linoleic acid 240mg + linolenic acid 60mg+ 95mg mineral oil )/day  Placebo ( 100mg/day ) | ADHD RS-Parent  Conners’ Teacher RS  Adverse events | DSM-IV | 7w |
| Rodríguez et al, 2019  Spain  RCT | omega-3  Placebo | 32  34 | Omega-3  ( DHA 1000mg+EPA 90mg+DPA 150mg )/sachet  ≤32kg 1 sache/dayt; >32kg 2 sachets/day  Placebo ( similar taste and smell ) | ADHD RS-Parent  Connes’ Parent RS  Adverse events | DSM-5 | 24w |
| Salehi et al, 2015  Iran  RCT | Methylphenidate (MPH)+omega-3  Methylphenidate (MPH)+Zinc sulfate  Methylphenidate (MPH)+Placebo | 50  50  50 | MPH + omega-3  ( <25 kg 100mg/day, 26-35kg 200mg/day, >35kg 400mg/day )  MPH+Zinc sulfate 22mg/day  MPH ( <20kg 10mg/day; >20kg 20mg/day )  + Placebo capsule ( sugar ) | Conners’ Parent RS  Conners’ Teacher RS  Adverse events | DSM-IV | 8w |
| Widenhorn-Müller et al,2014  Germany  RCT | omega-3  Placebo | 46  49 | omega-3  ( EPA 600mg+DHA 120mg )/day  Placebo ( olive oil ) | ADHS RS-Parent  Adverse events | DSM-IV | 16w |

Note: omega-3=omega-3 fatty acids, omega-6=omega-6 fatty acids, omega-3+6=omega-3 fatty acids plus omega-6 fatty acids, DHA=Docosahexaenoic acid, EPA=Eicosapentaenoic acid, DPA=Docosapentaenoic acid, LA=Linolenic acid, GLA=γ-linolenic acid, ALA=Alpha-linolenic acid, AA=Arachidonic acid, ADHD RS-Parent: ADHD Rating Scale-Parent, ADHD RS-Teacher=ADHD Rating Scale-Teacher, Conners’ RS Parent=Conners’ parent rating scale ( CPRS ), Conners’ RS Teacher=Conners teacher rating scale ( CTRS ), CGI=Clinical Global Impressions scale, CPT=Continuous Performance Test.
