# Supplementary material for: Safety and efficacy of antioxidant therapy in children and adolescents with attention deficit hyperactivity disorder: A systematic review and network meta-analysis
Source: PLoS One. 2024 Mar 28;19(3):e0296926. doi: 10.1371/journal.pone.0296926 (PMC10977718; doi:10.1371/journal.pone.0296926)
Supplement: S3 Table — (DOCX) [file pone.0296926.s004.docx]

Supplementary Material

## S4 Table. Network meta-analyses.

**A.Network meta-analysis for safety of antioxidant therapy**

| **Folic+MPH** | **-13.01**  **(-42.09,-0.93)** | **-12.97**  **(-42.11,-0.9)** | -0.44  (-2.04,1.01) | -12.49  (-45.98,14.53) | -12.75  (-46.22,14.23) | 12.1  (0,41.32) | -12.07  (-45.54,14.88) | -12.41  (-45.91,14.59) | -6.43  (-40.91,22.84) | 1.67  (-35.56,39.7) | -0.89  (-2.71,0.79) | 7.09  (-0.37,25.78) | -11.61  (-45.14,15.3) | -0.72  (-2.35,0.75) | -22.46  (-65.81,13.6) | -12.29  (-45.77,14.7) | -19.84  (-64.82,18.6) | -6.71  (-39.2,19.2) | -12.47  (-45.91,14.51) | 4.98  (-0.61,19.76) | -12.55  (-46.05,14.4) | -14  (-47.53,13.1) |
| --- | --- | --- | --- | --- | --- | --- | --- | --- | --- | --- | --- | --- | --- | --- | --- | --- | --- | --- | --- | --- | --- | --- |
| **13.01**  **(0.93,42.09)** | **sweet almond**  **-syrup**  **+ Placebo** | 0  (-32.23,31.87) | **12.54**  **(0.6,41.57)** | 1.66  (-35.4,39.95) | 1.38  (-35.67,39.67) | **27.64**  **(5.17,65.56)** | 2.07  (-34.97,40.39) | 1.73  (-35.32,40.03) | 7.88  (-30.27,47.63) | 16.43  (-24.93,62.3) | 12.09  (0.14,41.17) | **22.04**  **(4.01,54.06)** | 2.54  (-34.54,40.8) | **12.25**  **(0.32,41.31)** | -7.76  (-54.72,37.3) | 1.87  (-35.21,40.2) | -5.23  (-53.55,41.3) | 7.3  (-28.45,44.92) | 1.68  (-35.35,40) | **19.62**  **(3.39,50.33)** | 1.57  (-35.48,39.9) | 0.21  (-37,38.51) |
| **12.97**  **(0.9,42.11)** | 0  (-31.87,32.23) | **Acetyl-L-Carnitine+MPH** | **12.51**  **(0.58,41.64)** | 1.7  (-35.55,39.85) | 1.43  (-35.81,39.62) | **27.63**  **(5.27,65.71)** | 2.12  (-35.1,40.31) | 1.77  (-35.49,39.98) | 7.94  (-30.34,47.68) | 16.44  (-25.04,62.2) | **12.06**  **(0.11,41.23)** | **22.01**  **(4.05,54.39)** | 2.58  (-34.64,40.8) | **12.22**  **(0.29,41.36)** | -7.82  (-54.85,36.8) | 1.91  (-35.35,40.1) | -5.26  (-53.51,41.2) | 7.3  (-28.91,44.73) | 1.74  (-35.51,39.89) | **19.55**  **(3.32,50.56)** | 1.63  (-35.63,39.8) | 0.21  (-37.18,38.4) |
| 0.44  (-1.01,2.04) | **-12.54**  **(-41.57,-0.6)** | **-12.51**  **(-41.64,-0.58)** | **Placebo**  **+ MPH** | -12.01  (-45.49,14.89) | -12.3  (-45.74,14.59) | **12.55**  **(0.6,41.73)** | -11.63  (-45.07,15.25) | -11.93  (-45.41,14.92) | -5.99  (-40.36,23.26) | 2.13  (-35.1,40.16) | -0.43  (-1.34,0.39) | **7.53**  **(0.35,26.16)** | -11.12  (-44.61,15.8) | **-0.27**  **(-0.53,-0.09)** | -22  (-65.37,14.1) | -11.82  (-45.28,15.1) | -19.39  (-64.33,19.1) | -6.26  (-38.76,19.56) | -12.02  (-45.45,14.87) | **5.4**  **(0.26,20.13)** | -12.08  (-45.55,14.8) | -13.51  (-47.04,13.5) |
| 12.49  (-14.53,45.9) | -1.66  (-39.95,35.4) | -1.7  (-39.85,35.55) | 12.01  (-14.89,45.49) | **Acetyl-L**  **-Carnitine** | -0.26  (-0.89,0.31) | 26.67  (-5.93,68.26) | 0.42  (-0.19,0.96) | 0.07  (-0.17,0.32) | 5.23  (-0.67,18.87) | **13.01**  **(1.02,41.88)** | 11.58  (-15.35,45.01) | 21.12  (-8.34,57.64) | 0.88  (0.47,1.3) | 11.73  (-15.15,45.2) | -8.37  (-39.42,12.8) | 0.18  (-0.65,1.11) | -6.11  (-38.73,19.5) | **4.25**  **(0.46,14.5)** | 0.03  (-0.59,0.59) | 18.75  (-9.88,54.05) | -0.08  (-0.49,0.34) | -1.31  (-4.66,0.66) |
| 12.75  (-14.23,46.2) | -1.38  (-39.67,35.67) | -1.43  (-39.62,35.81) | 12.3  (-14.59,45.74) | 0.26  (-0.31,0.89) | **Quercetin** | 26.95  (-5.62,68.58) | **0.65**  **(0.23,1.32)** | 0.33  (-0.18,0.92) | 5.51  (-0.41,19.11) | **13.27**  **(1.28,42.15)** | 11.87  (-15.07,45.33) | 21.38  (-8.08,57.96) | **1.13**  **(0.58,1.8)** | 12.01  (-14.87,45.5) | -8.1  (-39.12,13.1) | 0.45  (-0.5,1.52) | -5.83  (-38.44,19.8) | **4.51**  **(0.75,14.75)** | 0.27  (-0.22,0.95) | 19.02  (-9.56,54.33) | 0.18  (-0.43,0.86) | -1.04  (-4.44,1) |
| -12.1  (-41.32,0) | **-27.64**  **(-65.56,-5.17)** | **-27.63**  **(-65.71,-5.27)** | **-12.55**  **(-41.73,-0.6)** | -26.67  (-68.26,5.93) | -26.95  (-68.58,5.62) | **Resveratrol**  **+ MPH** | -26.26  (-67.92,6.34) | -26.6  (-68.22,5.98) | -20.43  (-62.96,14.02) | -12.2  (-57.08,29.9) | **-13**  **(-42.16,-1.01)** | -4.41  (-35.2,18) | -25.78  (-67.41,6.82) | **-12.83**  **(-42,-0.88)** | -36.7  (-86.48,4.43) | -26.46  (-68.12,6.13) | -34.18  (-85.2,8.95) | -20.94  (-61.62,10.78) | -26.64  (-68.3,5.98) | -6.38  (-36.46,12.9) | -26.74  (-68.36,5.84) | -28.14  (-69.94,4.57) |
| 12.07  (-14.88,45.5) | -2.07  (-40.39,34.97) | -2.12  (-40.31,35.1) | 11.63  (-15.25,45.07) | -0.42  (-0.96,0.19) | **-0.65**  **(-1.32,-0.23)** | 26.26  (-6.34,67.92) | **MPH** | -0.35  (-0.82,0.22) | 4.82  (-1.07,18.47) | **12.58**  **(0.6,41.49)** | 11.2  (-15.74,44.62) | 20.69  (-8.76,57.27) | **0.44**  **(0.03,1.1)** | 11.35  (-15.54,44.8) | -8.79  (-39.78,12.4) | -0.23  (-1.17,0.82) | -6.53  (-39.15,19.1) | **3.81**  **(0.15,14.07)** | **-0.37**  **(-0.67,-0.16)** | 18.33  (-10.28,53.58) | -0.5  (-1.08,0.16) | -1.72  (-5.1,0.31) |
| 12.41  (-14.59,45.9) | -1.73  (-40.03,35.32) | -1.77  (-39.98,35.49) | 11.93  (-14.92,45.41) | -0.07  (-0.32,0.17) | -0.33  (-0.92,0.18) | 26.6  (-5.98,68.22) | 0.35  (-0.22,0.82) | **Placebo** | 5.16  (-0.73,18.79) | **12.93**  **(0.95,41.81)** | 11.52  (-15.42,44.92) | 21.04  (-8.4,57.56) | **0.8**  **(0.49,1.14)** | 11.66  (-15.21,45.1) | -8.43  (-39.47,12.7) | 0.11  (-0.68,1) | -6.18  (-38.79,19.4) | **4.18**  **(0.4,14.44)** | -0.04(  -0.61,0.46) | 18.69  (-9.93,53.95) | -0.15  (-0.49,0.18) | -1.38  (-4.72,0.57) |
| 6.43  (-22.84,40.9) | -7.88  (-47.63,30.27) | -7.94  (-47.68,30.34) | 5.99  (-23.26,40.36) | -5.23  (-18.87,0.67) | -5.51  (-19.11,0.41) | 20.43  (-14.02,62.9) | -4.82  (-18.47,1.07) | -5.16  (-18.79,0.73) | **Pycnogenol** | 7.19  (-11.26,37.3) | 5.52  (-23.72,39.92) | 15.01  (-16.71,52.5) | -4.35  (-17.99,1.56) | 5.7  (-23.57,40.1) | -14.85  (-47.29,8.3) | -5.06(  -18.67,0.93) | -12.52  (-46.84,14.8) | -0.51  (-15.36,11.79) | -5.21  (-18.85,0.69) | 12.64  (-18.16,48.81) | -5.31  (-18.95,0.59) | **-6.8**  **(-20.59,-0.2)** |
| -1.67  (-39.7,35.56) | -16.43  (-62.29,24.93) | -16.44  (-62.15,25.04) | -2.13  (-40.16,35.1) | **-13.01**  **(-41.88,-1.02)** | **-13.27**  **(-42.15,-1.28)** | 12.2  (-29.86,57.0) | **-12.58**  **(-41.49,-0.6)** | **-12.93**  **(-41.81,-0.95)** | -7.19  (-37.26,11.26) | **Ginkgo** | -2.59  (-40.62,34.68) | 6.79  (-33.02,47.1) | **-12.13**  **(-41.04,-0.2)** | -2.41  (-40.44,34.8) | -23.56  (-63.12,4.22) | -12.79  (-41.74,-0.8) | -21.08  (-62.57,10.1) | -7.6  (-37.1,9.05) | **-12.98**  **(-41.89,-1)** | 4.5  (-34.64,43.56) | **-13.09**  **(-41.97,-1.09)** | **-14.53**  **(-43.51,-2.1)** |
| 0.89  (-0.79,2.71) | **-12.09**  **(-41.17,-0.14)** | **-12.06**  **(-41.23,-0.11)** | 0.43  (-0.39,1.34) | -11.58  (-45.01,15.35) | -11.87  (-45.33,15.07) | **13**  **(1.01,42.16)** | -11.2  (-44.62,15.74) | -11.52  (-44.92,15.42) | -5.52  (-39.92,23.72) | 2.59  (-34.68,40.6) | **Ginkgo+MPH** | 7.97  (0.72,26.62) | -10.71  (-44.14,16.3) | 0.15  (-0.7,1.09) | -21.57  (-64.87,14.5) | -11.39  (-44.8,15.57) | -18.93  (-63.79,19.6) | -5.83  (-38.32,20.04) | -11.59  (-44.99,15.37) | **5.85**  **(0.58,20.62)** | -11.66  (-45.1,15.28) | -13.08  (-46.6,13.97) |
| -7.09  (-25.78,0.37) | **-22.04**  **(-54.06,-4.01)** | **-22.01**  **(-54.39,-4.05)** | **-7.53**  **(-26.16,-0.35)** | -21.12  (-57.64,8.34) | -21.38  (-57.96,8.08) | 4.41  (-18,35.2) | -20.69  (-57.27,8.76) | -21.04  (-57.56,8.4) | -15.01  (-52.51,16.71) | -6.79  (-47.14,33.1) | **-7.97**  **(-26.62,-0.72)** | **Zinc+MPH** | -20.24  (-56.76,9.21) | **-7.81**  **(-26.44,-0.6)** | -31.07  (-76.96,7.06) | -20.9  (-57.51,8.54) | -28.46  (-75.82,12.1) | -15.39  (-50.98,12.98) | -21.09  (-57.65,8.4) | -1.66  (-20.72,13.45) | -21.18  (-57.68,8.26) | -22.61  (-59.31,7.04) |
| 11.61  (-15.37,45.14) | -2.54  (-40.88,34.54) | -2.58  (-40.77,34.64) | 11.12  (-15.75,44.61) | **-0.88**  **(-1.3,-0.47)** | -1.13  (-1.8,-0.58) | 25.78  (-6.82,67.41) | **-0.44**  **(-1.1,-0.03)** | **-0.8**  **(-1.14,-0.49)** | 4.35  (-1.56,17.99) | **12.13**  **(0.14,41.04)** | 10.71  (-16.26,44.14) | 20.24  (-9.21,56.76) | **Zinc** | 10.84  (-16.05,44.3) | -9.26  (-40.26,11.9) | -0.69  (-1.55,0.25) | -6.99  (-39.58,18.6) | 3.37  (-0.41,13.6) | **-0.83**  **(-1.49,-0.36)** | 17.89  (-10.78,53.14) | **-0.95**  **(-1.43,-0.5)** | **-2.18**  **(-5.55,-0.21)** |
| 0.72  (-0.75,2.35) | **-12.25**  **(-41.31,-0.32)** | **-12.22**  **(-41.36,-0.29)** | **0.27**  **(0.09,0.53)** | -11.73  (-45.2,15.15) | -12.01  (-45.47,14.87) | **12.83**  **(0.88,42)** | -11.35  (-44.75,15.54) | -11.66  (-45.13,15.21) | -5.7  (-40.09,23.57) | 2.41  (-34.81,40.4) | -0.15  (-1.09,0.7) | **7.81**  **(0.63,26.44)** | -10.84  (-44.31,16.1) | **Vitamin D**  **+ MPH** | -21.74  (-65.1,14.36) | -11.54  (-44.99,15.4) | -19.11  (-64.04,19.4) | -5.98  (-38.49,19.87) | -11.73  (-45.13,15.17) | **5.68**  **(0.53,20.43)** | -11.81  (-45.27,15.1) | -13.22  (-46.74,13.8) |
| 22.46  (-13.64,65.8) | 7.76  (-37.32,54.72) | 7.82  (-36.75,54.85) | 22  (-14.07,65.37) | 8.37  (-12.8,39.42) | 8.1  (-13.08,39.12) | 36.7  (-4.43,86.48) | 8.79  (-12.37,39.78) | 8.43  (-12.72,39.47) | 14.85  (-8.3,47.29) | 23.56  (-4.22,63.12) | 21.57  (-14.49,64.87) | 31.07  (-7.06,76.96) | 9.26  (-11.95,40.3) | 21.74  (-14.36,65.1) | **Vitamin D** | 8.56  (-12.6,39.61) | 2.61  (-36.74,42.1) | 14.24  (-8.58,46.47) | 8.4  (-12.78,39.4) | 28.61  (-8.64,73.52) | 8.29  (-12.87,39.3) | 6.93  (-14.48,37.9) |
| 12.29  (-14.71,45.7) | -1.87  (-40.2,35.21) | -1.91  (-40.1,35.35) | 11.82  (-15.07,45.28) | -0.18  (-1.11,0.65) | -0.45  (-1.52,0.5) | 26.46  (-6.13,68.12) | 0.23  (-0.82,1.17) | -0.11  (-1,0.68) | 5.06  (-0.93,18.67) | 12.79  (0.81,41.74) | 11.39  (-15.57,44.8) | 20.9  (-8.54,57.51) | 0.69  (-0.25,1.55) | 11.54  (-15.36,44.9) | -8.56  (-39.61,12.6) | **Phosphatidy-lserine**  **+ omega-3** | -6.32  (-38.93,19.2) | **4.06**  **(0.1,14.27)** | -0.16  (-1.21,0.79) | 18.54  (-10.02,53.79) | -0.26  (-1.19,0.6) | -1.52  (-4.95,0.63) |
| 19.84  (-18.64,64.8) | 5.23  (-41.28,53.55) | 5.26  (-41.15,53.51) | 19.39  (-19.11,64.33) | 6.11  (-19.5,38.73) | 5.83  (-19.78,38.44) | 34.18  (-8.95,85.2) | 6.53  (-19.08,39.15) | 6.18  (-19.43,38.79) | 12.2  (-14.76,46.84) | 21.08  (-10.04,62.6) | 18.93  (-19.59,63.79) | 28.46  (-12.03,75.8) | 6.99  (-18.61,39.6) | 19.11  (-19.41,64.1) | -2.61  (-42.04,36.7) | 6.32  (-19.23,38.9) | **Phosphatidy-lserine** | 11.96  (-14.73,45.44) | 6.13  (-19.48,38.75) | 26.09  (-13.56,72.37) | 6.04  (-19.61,38.6) | 4.64  (-21.1,37.45) |
| 6.71  (-19.2,39.2) | -7.3  (-44.92,28.45) | -7.3  (-44.73,28.91) | 6.26  (-19.56,38.76) | **-4.25**  **(-14.5,-0.46)** | **-4.51**  **(-14.75,-0.75)** | 20.94  (-10.78,61.6) | **-3.81**  **(-14.07,-0.15)** | **-4.18**  **(-14.44,-0.4)** | 0.51  (-11.79,15.36) | 7.6  (-9.05,37.1) | 5.83  (-20.04,38.32) | 15.39  (-12.98,50.9) | -3.37  (-13.6,0.41) | 5.98  (-19.87,38.5) | -14.24  (-46.47,8.58) | **-4.06**  **(-14.27,-0.1)** | -11.96  (-45.44,14.7) | **omega-3+6**  **+ MPH** | **-4.19**  **(-14.48,-0.53)** | 12.98  (-14.52,47.36) | **-4.34**  **(-14.6,-0.53)** | **-5.96**  **(-16.28,-1)** |
| 12.47  (-14.51,45.9) | -1.68  (-40,35.35) | -1.74  (-39.89,35.51) | 12.02  (-14.87,45.45) | -0.03  (-0.59,0.59) | -0.27  (-0.95,0.22) | 26.64  (-5.98,68.3) | **0.37**  **(0.16,0.67)** | 0.04  (-0.46,0.61) | 5.21  (-0.69,18.85) | **12.98**  **(1,41.89)** | 11.59  (-15.37,44.99) | 21.09  (-8.4,57.65) | **0.83**  **(0.36,1.49)** | 11.73  (-15.17,45.2) | -8.4  (-39.4,12.78) | 0.16  (-0.79,1.21) | -6.13  (-38.75,19.5) | **4.19**  **(0.53,14.48)** | **omega-3+6** | 18.73  (-9.91,53.98) | -0.11  (-0.72,0.55) | -1.34  (-4.71,0.7) |
| -4.98  (-19.76,0.61) | **-19.62**  **(-50.33,-3.39)** | **-19.55**  **(-50.56,-3.32)** | **-5.4**  **(-20.13,-0.26)** | -18.75  (-54.05,9.88) | -19.02  (-54.33,9.56) | 6.38  (-12.9,36.46) | -18.33  (-53.58,10.28) | -18.69  (-53.95,9.93) | -12.64  (-48.81,18.16) | -4.5  (-43.56,34.6) | **-5.85**  **(-20.62,-0.58)** | 1.66  (-13.45,20.7) | -17.89  (-53.14,10.8) | **-5.68**  **(-20.43,-0.5)** | -28.61  (-73.52,8.64) | -18.54  (-53.79,10.1) | -26.09  (-72.37,13.6) | -12.98  (-47.36,14.52) | -18.73  (-53.98,9.91) | **omega-3**  **+ MPH** | -18.82  (-54.1,9.8) | -20.2  (-55.61,8.54) |
| 12.55  (-14.42,46.0) | -1.57  (-39.91,35.48) | -1.63  (-39.79,35.63) | 12.08  (-14.8,45.55) | 0.08  (-0.34,0.49) | -0.18  (-0.86,0.43) | 26.74  (-5.84,68.36) | 0.5  (-0.16,1.08) | 0.15  (-0.18,0.49) | 5.31  (-0.59,18.95) | **13.09**  **(1.09,41.97)** | 11.66  (-15.28,45.1) | 21.18  (-8.26,57.68) | **0.95**  **(0.5,1.43)** | 11.81  (-15.05,45.3) | -8.29  (-39.31,12.9) | 0.26  (-0.6,1.19) | -6.04  (-38.63,19.6) | **4.34**  **(0.53,14.6)** | 0.11  (-0.55,0.72) | 18.82  (-9.8,54.1) | **omega-3** | -1.22  (-4.57,0.7) |
| 14  (-13.1,47.53) | -0.21  (-38.51,37) | -0.21  (-38.41,37.18) | 13.51  (-13.5,47.04) | 1.31  (-0.66,4.66) | 1.04  (-1,4.44) | 28.14  (-4.57,69.94) | 1.72  (-0.31,5.1) | 1.38  (-0.57,4.72) | **6.8**  **(0.19,20.59)** | **14.53**  **(2.01,43.51)** | 13.08  (-13.97,46.6) | 22.61  (-7.04,59.31) | **2.18**  **(0.21,5.55)** | 13.22  (-13.77,46.7) | -6.93  (-37.98,14.5) | 1.52  (-0.63,4.95) | -4.64  (-37.45,21.1) | **5.96**  **(1,16.28)** | 1.34  (-0.7,4.71) | 20.2  (-8.54,55.61) | 1.22  (-0.7,4.57) | **omega-6** |

Note: All results are presented as Lg[ OR (95% CrI) ].

**B.Network meta-analysis for attention score of Conners’ Parent Rating Scale [ MD (95% CrI) ]**

| **Acetyl-L-carnitine** | 0.11  (-1.67,1.86) | 0.5  (-1.69,2.64) | -4.88  (-30.89,21.14) | -0.39  (-11.63,11.06) | -1.06  (-12.19,10.27) | 0.34  (-10.89,11.8) |
| --- | --- | --- | --- | --- | --- | --- |
| -0.11  (-1.86,1.67) | **Placebo** | 0.39  (-0.87,1.65) | -5.02  (-30.94,20.92) | -0.52  (-11.57,10.82) | -1.18  (-12.1,10.05) | 0.21  (-10.81,11.55) |
| -0.5  (-2.64,1.69) | -0.39  (-1.65,0.87) | **Pycnogenol** | -5.4  (-31.37,20.53) | -0.91  (-12.01,10.49) | -1.57  (-12.55,9.69) | -0.17  (-11.28,11.18) |
| 4.88  (-21.14,30.89) | 5.02  (-20.92,30.94) | 5.4  (-20.53,31.37) | **Phosphatidylserine**  **+ omega-3** | 4.42  (-23.78,32.5) | 3.78  (-24.39,31.85) | 5.21  (-23.1,33.29) |
| 0.39  (-11.06,11.63) | 0.52  (-10.82,11.57) | 0.91  (-10.49,12.01) | -4.42  (-32.5,23.78) | **omega-3+6** | -0.66  (-2.32,1.02) | 0.73  (-1.61,3.07) |
| 1.06  (-10.27,12.19) | 1.18  (-10.05,12.1) | 1.57  (-9.69,12.55) | -3.78  (-31.85,24.39) | 0.66  (-1.02,2.32) | **omega-3** | 1.39  (-0.26,3.05) |
| -0.34  (-11.8,10.89) | -0.21  (-11.55,10.81) | 0.17  (-11.18,11.28) | -5.21  (-33.29,23.1) | -0.73  (-3.07,1.61) | -1.39  (-3.05,0.26) | **omega-6** |

**C.Network meta-analysis for hyperactivity score of Conners’ Parent Rating Scale [ MD (95% CrI) ]**

| **Acetyl-L-carnitine** | 0.11  (-1.55,1.78) | -1.72  (-4.88,1.45) | -3.64  (-36.53,29.47) | 1.39  (-42.65,45.75) | -1.73  (-10.75,7.27) | 2.16  (-41.91,46.37) |
| --- | --- | --- | --- | --- | --- | --- |
| -0.11  (-1.78,1.55) | **Placebo** | -1.83  (-4.52,0.85) | -3.76  (-36.6,29.29) | 1.32  (-42.86,45.61) | -1.84  (-10.73,7) | 2.03  (-41.98,46.19) |
| 1.72  (-1.45,4.88) | 1.83  (-0.85,4.52) | **Pycnogenol** | -1.95  (-34.86,31.23) | 3.12  (-40.97,47.57) | -0.01  (-9.31,9.28) | 3.84  (-40.26,48.22) |
| 3.64  (-29.47,36.53) | 3.76  (-29.29,36.6) | 1.95  (-31.23,34.86) | **Phosphatidylserine**  **+ omega-3** | 4.94  (-50.3,60.27) | 1.88  (-32.35,35.84) | 5.79  (-49.13,60.83) |
| -1.39  (-45.75,42.65) | -1.32  (-45.61,42.86) | -3.12  (-47.57,40.97) | -4.94  (-60.27,50.3) | **omega-3+6** | -3.14  (-46.59,39.75) | 0.77  (-60.39,62.15) |
| 1.73  (-7.27,10.75) | 1.84  (-7,10.73) | 0.01  (-9.28,9.31) | -1.88  (-35.84,32.35) | 3.14  (-39.75,46.59) | **omega-3** | 3.91  (-39.21,47.32) |
| -2.16  (-46.37,41.91) | -2.03  (-46.19,41.98) | -3.84  (-48.22,40.26) | -5.79  (-60.83,49.13) | -0.77  (-62.15,60.39) | -3.91  (-47.32,39.21) | **omega-6** |

**D.Network meta-analysis for total score of Conners’ Parent Rating Scale (network A) [ MD (95% CrI) ]**

| **Acetyl-L-carnitine** | 0.1  (-1.1,1.3) | -3.64  (-9.73,2.44) | -2.91  (-17.91,12.1) | 0.6  (-26.9,28.05) | -0.08  (-7.7,7.51) | 2.54  (-30.75,35.79) |
| --- | --- | --- | --- | --- | --- | --- |
| -0.1  (-1.3,1.1) | **Placebo** | -3.71  (-9.69,2.24) | -3  (-17.97,11.92) | 0.5  (-27.01,28.01) | -0.17  (-7.72,7.34) | 2.43  (-30.81,35.7) |
| 3.64  (-2.44,9.73) | 3.71  (-2.24,9.69) | **Vitamin D** | 0.69  (-15.43,16.73) | 4.19  (-23.9,32.41) | 3.56  (-6.05,13.08) | 6.17  (-27.41,39.78) |
| 2.91  (-12.1,17.91) | 3  (-11.92,17.97) | -0.69  (-16.73,15.43) | **Phosphatidylserine**  **+ omega-3** | 3.52  (-27.82,34.55) | 2.86  (-13.32,18.88) | 5.42  (-30.76,41.66) |
| -0.6  (-28.05,26.9) | -0.5  (-28.01,27.01) | -4.19  (-32.41,23.9) | -3.52  (-34.55,27.82) | **omega-3+6** | -0.68  (-27.84,26.4) | 1.95  (-40.55,44.08) |
| 0.08  (-7.51,7.7) | 0.17  (-7.34,7.72) | -3.56  (-13.08,6.05) | -2.86  (-18.88,13.32) | 0.68  (-26.4,27.84) | **omega-3** | 2.6  (-29.82,35) |
| -2.54  (-35.79,30.75) | -2.43  (-35.7,30.81) | -6.17  (-39.78,27.41) | -5.42  (-41.66,30.76) | -1.95  (-44.08,40.55) | -2.6  (-35,29.82) | **omega-6** |

**E.Network meta-analysis for total score of Conners’ Parent Rating Scale (network B) [ MD (95% CrI) ]**

| **Folic+MPH** | 0.13  (-35.33,35.51) | -2.38  (-41.83,37.16) | -1.67  (-40.04,36.98) | 0.19  (-42.46,42.98) |
| --- | --- | --- | --- | --- |
| -0.13  (-35.51,35.33) | **Placebo+MPH** | -2.45  (-20.08,15.23) | -1.75  (-16.98,13.4) | 0.13  (-23.79,23.85) |
| 2.38  (-37.16,41.83) | 2.45  (-15.23,20.08) | **Zinc+MPH** | 0.78  (-22.5,23.99) | 2.62  (-22.01,27.21) |
| 1.67  (-36.98,40.04) | 1.7  5(-13.4,16.98) | -0.78  (-23.99,22.5) | **Vitamin D+MPH** | 1.89  (-26.46,29.97) |
| -0.19  (-42.98,42.46) | -0.13  (-23.85,23.79) | -2.62  (-27.21,22.01) | -1.89  (-29.97,26.46) | **omega-3+MPH** |

**F.Network meta-analysis for attention score of Conners’ Teacher Rating Scale [ MD (95% CrI) ]**

| **Acetyl-L-carnitine** | 0.11  (-1.67,1.88) | -2.29  (-8.15,3.64) | -1.59  (-23.21,20.32) | -0.73  (-12.17,10.69) |
| --- | --- | --- | --- | --- |
| -0.11  (-1.88,1.67) | **Placebo** | -2.4  (-7.96,3.21) | -1.73  (-23.28,20.14) | -0.84  (-12.14,10.42) |
| 2.29  (-3.64,8.15) | 2.4  (-3.21,7.96) | **Pycnogenol** | 0.68  (-21.62,23.16) | 1.54  (-11.03,14.12) |
| 1.59  (-20.32,23.21) | 1.73  (-20.14,23.28) | -0.68  (-23.16,21.62) | **Phosphatidylserine**  **+ omega-3** | 0.84  (-23.73,25.19) |
| 0.73  (-10.69,12.17) | 0.84  (-10.42,12.14) | -1.54  (-14.12,11.03) | -0.84  (-25.19,23.73) | **omega-3** |

**G.Network meta-analysis for hyperactivity score of Conners’ Teacher Rating Scale [ MD (95% CrI) ]**

| **Acetyl-L-carnitine** | -3.52  (-7.34,0.28) | -0.12  (-1.98,1.72) | -1.07  (-7.8,5.83) | -3.31  (-6.65,0.03) | -3.87  (-29.31,21.47) | -0.59  (-17.15,16.02) |
| --- | --- | --- | --- | --- | --- | --- |
| 3.52  (-0.28,7.34) | **MPH** | 3.39  (-0.08,6.73) | 2.46  (-4.84,9.81) | 0.21  (-1.63,2.04) | -0.37  (-26.01,25.22) | 2.91  (-13.93,19.71) |
| 0.12  (-1.72,1.98) | -3.39  (-6.73,0.08) | **Placebo** | -0.95  (-7.43,5.62) | -3.19  (-5.96,-0.42) | -3.76  (-29.19,21.51) | -0.47  (-16.93,16.04) |
| 1.07  (-5.83,7.8) | -2.46  (-9.81,4.84) | 0.95  (-5.62,7.43) | **Pycnogenol** | -2.26  (-9.36,4.78) | -2.83  (-28.98,23.34) | 0.46  (-17.17,18.24) |
| 3.31  (-0.03,6.65) | -0.21  (-2.04,1.63) | 3.19  (-0.42,5.96) | 2.26  (-4.78,9.36) | **Zinc** | -0.58  (-26.14,24.83) | 2.71  (-14,19.43) |
| 3.87  (-21.47,29.31) | 0.37  (-25.22,26.01) | 3.76  (-21.51,29.19) | 2.83  (-23.34,28.98) | 0.58  (-24.83,26.14) | **Phosphatidylserine**  **+ omega-3** | 3.31  (-26.68,33.37) |
| 0.59  (-16.02,17.15) | -2.91  (-19.71,13.93) | 0.47  (-16.04,16.93) | -0.46  (-18.24,17.17) | -2.71  (-19.43,14) | -3.31  (-33.37,26.68) | **omega-3** |

**H.Network meta-analysis for total score of Conners’ Teacher Rating Scale [ MD (95% CrI) ]**

| **Acetyl-L-carnitine** | -1.31  (-5,2.41) | 0.44  (-1.04,1.93) | -1.16  (-4.35,2.02) | -2.15  (-23.49,19.49) | -0.86  (-5.01,3.27) | 0.35  (-3.39,4.05) |
| --- | --- | --- | --- | --- | --- | --- |
| 1.31  (-2.41,5) | **MPH** | 1.75  (-1.64,5.13) | 0.15  (-1.76,2.04) | -0.87  (-22.48,21.02) | 0.45  (-4.72,5.56) | 1.66  (-3.19,6.46) |
| -0.44  (-1.93,1.04) | -1.75  (-5.13,1.64) | **Placebo** | -1.6  (-4.4,1.2) | -2.61  (-23.87,19) | -1.3  (-5.17,2.55) | -0.09  (-3.51,3.32) |
| 1.16  (-2.02,4.35) | -0.15  (-2.04,1.76) | 1.6  (-1.2,4.4) | **Zinc** | -1.02  (-22.59,20.77) | 0.3  (-4.48,5.06) | 1.51  (-2.92,5.91) |
| 2.15  (-19.49,23.49) | 0.87  (-21.02,22.48) | 2.61  (-19,23.87) | 1.02  (-20.77,22.59) | **Phosphatidylserine**  **+ omega-3** | 1.28  (-20.68,22.93) | 2.5  (-19.37,24.08) |
| 0.86  (-3.27,5.01) | -0.45  (-5.56,4.72) | 1.3  (-2.55,5.17) | -0.3  (-5.06,4.48) | -1.28  (-22.93,20.68) | **omega-3+6** | 1.22  (-4,6.38) |
| -0.35  (-4.05,3.39) | -1.66  (-6.46,3.19) | 0.09  (-3.32,3.51) | -1.51  (-5.91,2.92) | -2.5  (-24.08,19.37) | -1.22  (-6.38,4) | **omega-3** |

**I.Network meta-analysis for attention score of ADHD Rating Scale-Parent [ MD (95% CrI) ]**

| **Folic+MPH** | -1.02  (-20.9,18.84) | -0.3  (-15.8,15.28) | -1.04  (-28.74,26.77) | -1.67  (-20.78,17.57) | -2.42  (-25.09,20.25) | -0.98  (-25.13,23.26) | 2.47  (-35.93,41.34) | 2.14  (-27.17,31.43) | -2.18  (-22.07,17.85) | -2.51  (-25.33,20.19) | -10.04  (-85.29,64.87) | -2.53  (-27.31,22.31) | -3.31  (-28.14,21.78) | -1.67  (-21.74,18.53) | -1.54  (-24.48,21.41) | -0.73  (-18.78,17.44) | -0.48  (-24.65,23.79) |
| --- | --- | --- | --- | --- | --- | --- | --- | --- | --- | --- | --- | --- | --- | --- | --- | --- | --- |
| 1.02  (-18.84,20.9) | **Sweet**  **almond syrup**  **+Placebo** | 0.69  (-11.52,13.19) | -0.04  (-26.08,26.03) | -0.61  (-17.25,16.11) | -1.35  (-22.13,19.1) | 0.04  (-22.04,22.16) | 3.52  (-34.08,41.43) | 3.23  (-24.51,31.01) | -1.13  (-18.65,16.6) | -1.47  (-22.27,19.09) | -9.04  (-83.53,65.53) | -1.48  (-24.23,21.31) | -2.22  (-25.22,20.88) | -0.61  (-18.21,17.12) | -0.51  (-21.25,20.28) | 0.26  (-15.11,15.95) | 0.53  (-21.58,22.73) |
| 0.3  (-15.28,15.8) | -0.69  (-13.19,11.52) | **Placebo**  **+ MPH** | -0.73  (-23.96,22.3) | -1.37  (-12.65,9.98) | -2.13  (-18.87,14.48) | -0.68  (-19.28,17.81) | 2.83  (-32.86,38.64) | 2.46  (-22.53,27.48) | -1.89  (-14.15,10.62) | -2.24  (-19.12,14.42) | -9.87  (-83.1,63.66) | -2.28  (-21.67,17.03) | -3.01  (-22.47,16.53) | -1.35  (-14.25,11.5) | -1.28  (-18.34,15.64) | -0.43  (-9.71,8.99) | -0.2  (-18.81,18.36) |
| 1.04  (-26.77,28.74) | 0.04  (-26.03,26.08) | 0.73  (-22.3,23.96) | **Quercetin** | -0.58  (-26.17,25.01) | -1.38  (-19.32,16.63) | 0.08  (-13.61,13.91) | 3.62  (-30.08,37.14) | 3.17  (-22.47,28.95) | -1.07  (-27.37,25.1) | -1.5  (-19.43,16.6) | -8.88  (-86.31,67.69) | -1.48  (-16.26,13.37) | -2.18  (-17.38,12.91) | -0.57  (-19.8,18.77) | -0.5  (-16.34,15.28) | 0.34  (-24.47,25.33) | 0.55  (-13.21,14.45) |
| 1.67  (-17.57,20.78) | 0.61  (-16.11,17.25) | 1.37  (-9.98,12.65) | 0.58  (-25.01,26.17) | **Resveratrol**  **+ MPH** | -0.79  (-20.97,19.29) | 0.66  (-21.06,22.34) | 4.21  (-33.2,41.71) | 3.79  (-23.58,31.31) | -0.48  (-17.32,16.37) | -0.88  (-21.13,19.27) | -8.44  (-82.74,66.03) | -0.85  (-23.29,21.41) | -1.61  (-24.18,20.89) | 0.02  (-17.18,17.08) | 0.13  (-20.3,20.35) | 0.94  (-13.7,15.56) | 1.15  (-20.6,22.82) |
| 2.42  (-20.25,25.09) | 1.35  (-19.1,22.13) | 2.13  (-14.48,18.87) | 1.38  (-16.63,19.32) | 0.79  (-19.29,20.97) | **MPH** | 1.46  (-10,12.86) | 5.06  (-27.71,37.32) | 4.56  (-13.96,23.19) | 0.29  (-20.38,21.14) | -0.1  (-1.63,1.43) | -7.62  (-82.6,67.66) | -0.07  (-12.78,12.56) | -0.83  (-13.87,12.19) | 0.8  (-9.88,11.5) | 0.87  (-7.74,9.49) | 1.75  (-17.45,20.86) | 1.94  (-9.58,13.4) |
| 0.98  (-23.26,25.13) | -0.04  (-22.16,22.04) | 0.68  (-17.81,19.28) | -0.08  (-13.91,13.61) | -0.66  (-22.34,21.06) | -1.46  (-12.86,10) | **Placebo** | 3.62  (-26.97,33.85) | 3.09  (-18.54,24.91) | -1.23  (-23.33,21.2) | -1.55  (-13.08,9.98) | -9.1  (-84.81,66.44) | -1.53  (-7.02,4) | -2.29  (-8.51,3.99) | -0.64  (-14.15,12.8) | -0.6  (-8.17,7.01) | 0.3  (-20.56,20.96) | 0.48  (-0.77,1.73) |
| -2.47  (-41.34,35.93) | -3.52  (-41.43,34.08) | -2.83  (-38.64,32.86) | -3.62  (-37.14,30.08) | -4.21  (-41.71,33.2) | -5.06  (-37.32,27.71) | -3.62  (-33.85,26.97) | **Pycnogenol** | -0.49  (-37.73,37.4) | -4.73  (-42.47,33.04) | -5.17  (-37.47,27.61) | -12.55  (-94.23,68.48) | -5.15  (-35.86,25.85) | -5.87  (-36.7,25.24) | -4.25  (-37.37,29.29) | -4.15  (-35.36,27.26) | -3.3  (-40.23,33.69) | -3.11  (-33.4,27.48) |
| -2.14  (-31.43,27.17) | -3.23  (-31.01,24.51) | -2.46  (-27.48,22.53) | -3.17  (-28.95,22.47) | -3.79  (-31.31,23.58) | -4.56  (-23.19,13.96) | -3.09  (-24.91,18.54) | 0.49  (-37.4,37.73) | **Ginko** | -4.35  (-32.01,23.71) | -4.67  (-23.34,13.92) | -12.21  (-89.02,65.52) | -4.65  (-27.13,17.67) | -5.39  (-28.13,17.11) | -3.81  (-25.22,17.65) | -3.74  (-24.16,16.64) | -2.89  (-29.45,23.82) | -2.61  (-24.45,19.07) |
| 2.18  (-17.85,22.07) | 1.13  (-16.6,18.65) | 1.89  (-10.62,14.15) | 1.07  (-25.1,27.37) | 0.48  (-16.37,17.32) | -0.29  (-21.14,20.38) | 1.23  (-21.2,23.33) | 4.73  (-33.04,42.47) | 4.35  (-23.71,32.01) | **Ginko+MPH** | -0.39  (-21.3,20.31) | -7.91  (-82.64,66.15) | -0.3  (-23.52,22.48) | -1.02  (-24.28,21.86) | 0.53  (-17.42,18.28) | 0.59  (-20.45,21.32) | 1.46  (-14.14,17.06) | 1.73  (-20.8,23.84) |
| 2.51  (-20.19,25.33) | 1.47  (-19.09,22.27) | 2.24  (-14.42,19.12) | 1.5  (-16.6,19.43) | 0.88  (-19.27,21.13) | 0.1  (-1.43,1.63) | 1.55  (-9.98,13.08) | 5.17  (-27.61,37.47) | 4.67  (-13.92,23.34) | 0.39  (-20.31,21.3) | **Zinc** | -7.55  (-82.5,67.75) | 0.03  (-12.83,12.77) | -0.73  (-13.85,12.37) | 0.88  (-9.94,11.69) | 0.97  (-7.78,9.72) | 1.85  (-17.43,21.05) | 2.03  (-9.55,13.62) |
| 10.04  (-64.87,85.29) | 9.04  (-65.53,83.53) | 9.87  (-63.66,83.1) | 8.88  (-67.69,86.31) | 8.44  (-66.03,82.74) | 7.62  (-67.66,82.6) | 9.1  (-66.44,84.81) | 12.55  (-68.48,94.23) | 12.21  (-65.52,89.02) | 7.9  1(-66.15,82.64) | 7.55  (-67.75,82.5) | **Vitamin D**  **+ MPH** | 7.5  (-68.28,83.33) | 6.74  (-68.73,82.75) | 8.4  (-66.03,82.63) | 8.52  (-66.71,83.64) | 9.3  (-64.58,83.36) | 9.6  (-65.97,85.28) |
| 2.53  (-22.31,27.31) | 1.48  (-21.31,24.23) | 2.28  (-17.03,21.67) | 1.48  (-13.37,16.26) | 0.85  (-21.41,23.29) | 0.07  (-12.56,12.78) | 1.53  (-4,7.02) | 5.15  (-25.85,35.86) | 4.65  (-17.67,27.13) | 0.3  (-22.48,23.52) | -0.03  (-12.77,12.83) | -7.5  (-83.33,68.28) | **Vitamin D** | -0.75  (-9.03,7.6) | 0.87  (-13.6,15.39) | 0.93  (-8.39,10.34) | 1.86  (-19.58,23.25) | 2.02  (-3.65,7.64) |
| 3.31  (-21.78,28.14) | 2.22  (-20.88,25.22) | 3.01  (-16.53,22.47) | 2.18  (-12.91,17.38) | 1.61  (-20.89,24.18) | 0.83  (-12.19,13.87) | 2.29  (-3.99,8.51) | 5.87  (-25.24,36.7) | 5.39  (-17.11,28.13) | 1.02  (-21.86,24.28) | 0.73  (-12.37,13.85) | -6.74  (-82.75,68.73) | 0.75  (-7.6,9.03) | **Phosphatid**  **-ylserine** | 1.63  (-13.28,16.37) | 1.68  (-8.08,11.43) | 2.56  (-19.18,24.18) | 2.77  (-3.62,9.14) |
| 1.67  (-18.53,21.74) | 0.61  (-17.12,18.21) | 1.35  (-11.5,14.25) | 0.57  (-18.77,19.8) | -0.02  (-17.08,17.18) | -0.8  (-11.5,9.88) | 0.64  (-12.8,14.15) | 4.25  (-29.29,37.37) | 3.81  (-17.65,25.22) | -0.53  (-18.28,17.42) | -0.88  (-11.69,9.94) | -8.4  (-82.63,66.03) | -0.87  (-15.39,13.6) | -1.63  (-16.37,13.28) | **omega-3+6**  **+ MPH** | 0.06  (-11.16,11.22) | 0.91  (-14.93,16.75) | 1.14  (-12.37,14.66) |
| 1.54  (-21.41,24.48) | 0.51  (-20.28,21.25) | 1.28  (-15.64,18.34) | 0.5  (-15.28,16.34) | -0.13  (-20.35,20.3) | -0.87  (-9.49,7.74) | 0.6  (-7.01,8.17) | 4.15  (-27.26,35.36) | 3.74  (-16.64,24.16) | -0.59  (-21.32,20.45) | -0.97  (-9.72,7.78) | -8.52  (-83.64,66.71) | -0.93  (-10.34,8.39) | -1.68  (-11.43,8.08) | -0.06  (-11.22,11.16) | **omega-3+6** | 0.88  (-18.55,20.16) | 1.08  (-6.64,8.71) |
| 0.73  (-17.44,18.78) | -0.26  (-15.95,15.11) | 0.43  (-8.99,9.71) | -0.34  (-25.33,24.47) | -0.94  (-15.56,13.7) | -1.75  (-20.86,17.45) | -0.3  (-20.96,20.56) | 3.3  (-33.69,40.23) | 2.89  (-23.82,29.45) | -1.46  (-17.06,14.14) | -1.85  (-21.05,17.43) | -9.3  (-83.36,64.58) | -1.86  (-23.25,19.58) | -2.56  (-24.18,19.18) | -0.91  (-16.75,14.93) | -0.88  (-20.16,18.55) | **omega-3**  **+ MPH** | 0.18  (-20.59,21.13) |
| 0.48  (-23.79,24.65) | -0.53  (-22.73,21.58) | 0.2  (-18.36,18.81) | -0.55  (-14.45,13.21) | -1.15  (-22.82,20.6) | -1.94  (-13.4,9.58) | -0.48  (-1.73,0.77) | 3.11  (-27.48,33.4) | 2.61  (-19.07,24.45) | -1.73  (-23.84,20.8) | -2.03  (-13.62,9.55) | -9.6  (-85.28,65.97) | -2.02  (-7.64,3.65) | -2.77  (-9.14,3.62) | -1.14  (-14.66,12.37) | -1.08  (-8.71,6.64) | -0.18  (-21.13,20.59) | **omega-3** |

1. **Network meta-analysis for hyperactivity score of ADHD Rating Scale-Parent [ MD (95% CrI) ]**

| **Folic+MPH** | -0.54  (-17.77,16.75) | -0.04  (-7.84,7.97) | 12.18  (-16.05,40.57) | -2.2  (-15.39,10.99) | 7.63  (-10.78,25.89) | 12.03  (-10.53,34.6) | 11.79  (-48.39,72.09) | 12.67  (-11.59,36.72) | -0.64  (-17.45,16.21) | 7.61  (-11.04,26.03) | 1.9  (-51.09,54.66) | 10.84  (-12.14,33.81) | 10.53  (-12.47,33.53) | 1.96  (-10.66,14.68) | 10.9  (-8.84,30.63) | 1.73  (-13.46,16.96) | 11.67  (-10.8,34.34) |
| --- | --- | --- | --- | --- | --- | --- | --- | --- | --- | --- | --- | --- | --- | --- | --- | --- | --- |
| 0.54  (-16.75,17.77) | **Sweet almond syrup**  **+ Placebo** | 0.5  (-14.87,15.86) | 12.7  (-18.33,44.06) | -1.67  (-20.25,16.84) | 8.14  (-14.27,30.71) | 12.52  (-13.44,38.61) | 12.45  (-49.29,74.07) | 13.15  (-14.3,40.73) | -0.08  (-21.29,21.32) | 8.1  (-14.41,30.76) | 2.3  (-52.38,56.81) | 11.31  (-15.13,37.83) | 11.07  (-15.38,37.49) | 2.52  (-15.78,20.78) | 11.42  (-12.37,35.22) | 2.22  (-17.88,22.26) | 12.2  (-13.84,38.39) |
| 0.04  (-7.97,7.84) | -0.5  (-15.86,14.87) | **Placebo**  **+ MPH** | 12.18  (-15,39.58) | -2.2  (-12.74,8.32) | 7.64  (-8.93,24.17) | 12.09  (-9.06,33.14) | 11.75  (-47.73,71.59) | 12.71  (-10.17,35.33) | -0.63  (-15.35,14.3) | 7.6  (-9.14,24.28) | 1.91  (-50.44,54.15) | 10.86  (-10.77,32.27) | 10.6  (-10.96,32.13) | 2  (-7.96,12) | 10.92  (-7.16,29.14) | 1.73  (-11.27,14.6) | 11.76  (-9.37,32.89) |
| -12.18  (-40.57,16.05) | -12.7  (-44.06,18.33) | -12.18  (-39.58,15) | **Quercetin** | -14.42  (-43.7,14.59) | -4.57  (-29.66,20.36) | -0.13  (-17.46,16.89) | -0.39  (-58.42,57.53) | 0.41  (-29.26,30.21) | -12.79  (-43.91,18.07) | -4.6  (-29.87,20.44) | -10.38  (-69.5,48.24) | -1.34  (-19.26,16.31) | -1.63  (-19.46,15.9) | -10.22  (-35.79,15.06) | -1.27  (-22.01,19.05) | -10.46  (-40.65,19.72) | -0.47  (-17.87,16.72) |
| 2.2  (-10.99,15.39) | 1.67  (-16.84,20.25) | 2.2  (-8.32,12.74) | 14.42  (-14.59,43.7) | **Resveratrol**  **+ MPH** | 9.85  (-9.71,29.48) | 14.35  (-9.37,37.82) | 13.95  (-46.38,74.75) | 14.86  (-10.26,40.15) | 1.6  (-16.42,19.85) | 9.84  (-9.92,29.61) | 4.06  (-49.45,57.36) | 13.13  (-10.82,37.08) | 12.86  (-11.35,36.72) | 4.2  (-10.26,18.81) | 13.13  (-7.83,34.11) | 3.89  (-12.81,20.58) | 14.02  (-9.72,37.57) |
| -7.63  (-25.89,10.78) | -8.14  (-30.71,14.27) | -7.64  (-24.17,8.93) | 4.57  (-20.36,29.66) | -9.85  (-29.48,9.71) | **MPH** | 4.4  (-13.75,22.62) | 4.18  (-54.23,62.71) | 5.02  (-10.81,20.63) | -8.26  (-30.26,14.02) | -0.03  (-2.31,2.24) | -5.88  (-60.56,49.18) | 3.21  (-15.55,21.94) | 2.95  (-15.87,21.7) | -5.65  (-18.72,7.49) | 3.33  (-11.3,17.8) | -5.98  (-26.8,15.13) | 4.07  (-14.12,22.38) |
| -12.03  (-34.6,10.53) | -12.52  (-38.61,13.44) | -12.09  (-33.14,9.06) | 0.13  (-16.89,17.46) | -14.35  (-37.82,9.37) | -4.4  (-22.62,13.75) | **Placebo** | -0.14  (-55.4,55.43) | 0.55  (-23.58,24.64) | -12.66  (-38.48,13.02) | -4.42  (-22.77,13.91) | -10.22  (-66.89,46.12) | -1.22  (-5.87,3.48) | -1.49  (-5.97,2.96) | -10.08  (-28.64,8.46) | -1.12  (-12.13,9.82) | -10.39  (-34.97,14.51) | -0.33  (-1.98,1.33) |
| -11.79  (-72.09,48.39) | -12.45  (-74.07,49.29) | -11.75  (-71.59,47.73) | 0.39  (-57.53,58.42) | -13.95  (-74.75,46.38) | -4.18  (-62.71,54.23) | 0.14  (-55.43,55.4) | **Pycnogenol** | 0.81  (-60.04,61.48) | -12.3  (-73.75,49.07) | -4.21  (-62.92,54.2) | -9.99  (-89.45,69.25) | -1  (-56.82,54.49) | -1.42  (-57.14,54.05) | -9.85  (-68.9,49.03) | -0.9  (-57.63,55.62) | -10.18  (-71.12,51) | -0.17  (-55.81,55.12) |
| -12.67  (-36.72,11.59) | -13.15  (-40.73,14.3) | -12.71  (-35.33,10.17) | -0.41  (-30.21,29.26) | -14.86  (-40.15,10.26) | -5.02  (-20.63,10.81) | -0.55  (-24.64,23.58) | -0.81  (-61.48,60.04) | **Ginkgo** | -13.17  (-40.27,14.1) | -5.05  (-20.78,10.86) | -10.84  (-67.69,45.95) | -1.78  (-26.34,22.78) | -2.03  (-26.47,22.48) | -10.66  (-31.1,9.77) | -1.69  (-23.23,19.77) | -10.99  (-37.17,15.26) | -0.9  (-25.01,23.38) |
| 0.64  (-16.21,17.45) | 0.08  (-21.32,21.29) | 0.63  (-14.3,15.35) | 12.79  (-18.07,43.91) | -1.6  (-19.85,16.42) | 8.26  (-14.02,30.26) | 12.66  (-13.02,38.48) | 12.3  (-49.07,73.75) | 13.17  (-14.1,40.27) | **Ginkgo+MPH** | 8.25  (-14.1,30.36) | 2.44  (-52.11,56.76) | 11.44  (-14.74,37.64) | 11.15  (-14.88,37.4) | 2.56  (-15.43,20.38) | 11.51  (-11.91,34.94) | 2.33  (-17.46,21.92) | 12.32  (-13.41,38.21) |
| -7.61  (-26.03,11.04) | -8.1  (-30.76,14.41) | -7.6  (-24.28,9.14) | 4.6  (-20.44,29.87) | -9.84  (-29.61,9.92) | 0.03  (-2.24,2.31) | 4.42  (-13.91,22.77) | 4.21  (-54.2,62.92) | 5.05  (-10.86,20.78) | -8.25  (-30.36,14.1) | **Zinc** | -5.83  (-60.55,49.24) | 3.23  (-15.62,22.15) | 2.97  (-15.91,21.87) | -5.62  (-18.88,7.75) | 3.36  (-11.49,17.99) | -5.92  (-26.93,15.35) | 4.11  (-14.29,22.49) |
| -1.9  (-54.66,51.09) | -2.3  (-56.81,52.38) | -1.91  (-54.15,50.44) | 10.38  (-48.24,69.5) | -4.06  (-57.36,49.45) | 5.88  (-49.18,60.56) | 10.22  (-46.12,66.89) | 9.99  (-69.25,89.45) | 10.84  (-45.95,67.69) | -2.44  (-56.76,52.11) | 5.83  (-49.24,60.55) | **Vitamin D**  **+ MPH** | 8.94  (-47.52,65.79) | 8.79  (-47.67,65.63) | 0.19  (-52.95,53.46) | 9.11  (-46.2,64.42) | -0.18  (-53.75,53.68) | 9.87  (-46.4,66.57) |
| -10.84  (-33.81,12.14) | -11.31  (-37.83,15.13) | -10.86  (-32.27,10.77) | 1.34  (-16.31,19.26) | -13.13  (-37.08,10.82) | -3.21  (-21.94,15.55) | 1.22  (-3.48,5.87) | 1  (-54.49,56.82) | 1.78  (-22.78,26.34) | -11.44  (-37.64,14.74) | -3.23  (-22.15,15.62) | -8.94  (-65.79,47.52) | **Vitamin D** | -0.27  (-6.75,6.16) | -8.87  (-28.02,10.21) | 0.12  (-11.9,12.09) | -9.24  (-34.13,16.16) | 0.88  (-4.1,5.86) |
| -10.53  (-33.53,12.47) | -11.07  (-37.49,15.38) | -10.6  (-32.13,10.96) | 1.63  (-15.9,19.46) | -12.86  (-36.72,11.35) | -2.95  (-21.7,15.87) | 1.49  (-2.96,5.97) | 1.42  (-54.05,57.14) | 2.03  (-22.48,26.47) | -11.15  (-37.4,14.88) | -2.97  (-21.87,15.91) | -8.79  (-65.63,47.67) | 0.27  (-6.16,6.75) | **Phosphatid-ylserine** | -8.59  (-27.66,10.52) | 0.36  (-11.57,12.22) | -8.9  (-33.92,16.4) | 1.15  (-3.56,5.95) |
| -1.96  (-14.68,10.66) | -2.52  (-20.78,15.78) | -2  (-12,7.96) | 10.22  (-15.06,35.79) | -4.2  (-18.81,10.26) | 5.65  (-7.49,18.72) | 10.08  (-8.46,28.64) | 9.85  (-49.03,68.9) | 10.66  (-9.77,31.1) | -2.56  (-20.38,15.43) | 5.62  (-7.75,18.88) | -0.19  (-53.46,52.95) | 8.87  (-10.21,28.02) | 8.59  (-10.52,27.66) | **omega-3+6**  **+ MPH** | 8.98  (-6.19,24.03) | -0.28  (-16.67,16.1) | 9.75  (-8.87,28.39) |
| -10.9  (-30.63,8.84) | -11.42  (-35.22,12.37) | -10.92  (-29.14,7.16) | 1.27  (-19.05,22.01) | -13.13  (-34.11,7.83) | -3.33  (-17.8,11.3) | 1.12  (-9.82,12.13) | 0.9  (-55.62,57.63) | 1.69  (-19.77,23.23) | -11.51  (-34.94,11.91) | -3.36  (-17.99,11.49) | -9.11  (-64.42,46.2) | -0.12  (-12.09,11.9) | -0.36  (-12.22,11.57) | -8.98  (-24.03,6.19) | **omega-3+6** | -9.28  (-31.37,13.09) | 0.8  (-10.28,11.97) |
| -1.73  (-16.96,13.46) | -2.22  (-22.26,17.88) | -1.73  (-14.6,11.27) | 10.46  (-19.72,40.65) | -3.89  (-20.58,12.81) | 5.98  (-15.13,26.8) | 10.39  (-14.51,34.97) | 10.18  (-51,71.12) | 10.99  (-15.26,37.17) | -2.33  (-21.92,17.46) | 5.92  (-15.35,26.93) | 0.18  (-53.68,53.75) | 9.24  (-16.16,34.13) | 8.9  (-16.4,33.92) | 0.28  (-16.1,16.67) | 9.28  (-13.09,31.37) | **omega-3**  **+ MPH** | 10.07  (-14.85,34.69) |
| -11.67  (-34.34,10.8) | -12.2  (-38.39,13.84) | -11.76  (-32.89,9.37) | 0.47  (-16.72,17.87) | -14.02  (-37.57,9.72) | -4.07  (-22.38,14.12) | 0.33  (-1.33,1.98) | 0.17  (-55.12,55.81) | 0.9  (-23.38,25.01) | -12.32  (-38.21,13.41) | -4.11  (-22.49,14.29) | -9.87  (-66.57,46.4) | -0.88  (-5.86,4.1) | -1.15  (-5.95,3.56) | -9.75  (-28.39,8.87) | -0.8  (-11.97,10.28) | -10.07  (-34.69,14.85) | **omega-3** |

**K.Network meta-analysis for total score of ADHD Rating Scale-Parent (network A) [ MD (95% CrI) ]**

| **Quercetin** | 0.3  (-20.44,21.31) | -0.24  (-25.3,24.91) | 7.95  (-26.55,43.05) | 0.33  (-20.57,21.36) | -3.13  (-29.44,23.17) | -3.74  (-31.13,23.54) | -0.4  (-25.52,24.77) | -0.65  (-25.77,24.56) |
| --- | --- | --- | --- | --- | --- | --- | --- | --- |
| -0.3  (-21.31,20.44) | **MPH** | -0.51  (-33.32,32.07) | 7.61  (-19.92,35.59) | 0.02  (-1.58,1.61) | -3.43  (-36.99,30.05) | -4.1  (-38.56,30.37) | -0.7  (-33.5,31.99) | -0.94  (-33.69,31.68) |
| 0.24  (-24.91,25.3) | 0.51  (-32.07,33.32) | **Placebo** | 8.19  (-34.63,51.3) | 0.58  (-32.14,33.3) | -2.89  (-10.5,4.7) | -3.52  (-14.3,7.24) | -0.17  (-1.81,1.47) | -0.41  (-1.81,0.98) |
| -7.95  (-43.05,26.55) | -7.61  (-35.59,19.92) | -8.19  (-51.3,34.63) | **Ginkgo** | -7.6  (-35.59,20) | -11.05  (-54.8,32.25) | -11.71  (-55.99,32.49) | -8.35  (-51.58,34.49) | -8.62  (-51.78,34.27) |
| -0.33  (-21.36,20.57) | -0.02  (-1.61,1.58) | -0.58  (-33.3,32.14) | 7.6  (-20,35.59) | **Zinc** | -3.46  (-37.08,30.15) | -4.13  (-38.64,30.35) | -0.74  (-33.48,32.04) | -0.99  (-33.75,31.77) |
| 3.13  (-23.17,29.44) | 3.43  (-30.05,36.99) | 2.89  (-4.7,10.5) | 11.05  (-32.25,54.8) | 3.46  (-30.15,37.08) | **Vitamin D** | -0.64  (-13.75,12.59) | 2.73  (-5.06,10.52) | 2.49  (-5.23,10.25) |
| 3.74  (-23.54,31.13) | 4.1  (-30.37,38.56) | 3.52  (-7.24,14.3) | 11.71  (-32.49,55.99) | 4.13  (-30.35,38.64) | 0.64  (-12.59,13.75) | **Phosphatidylserine** | 3.35  (-7.57,14.25) | 3.12  (-7.78,14.01) |
| 0.4  (-24.77,25.52) | 0.7  (-31.99,33.5) | 0.17  (-1.47,1.81) | 8.35  (-34.49,51.58) | 0.74  (-32.04,33.48) | -2.73  (-10.52,5.06) | -3.35  (-14.25,7.57) | **omega-3+6** | -0.23  (-2.4,1.94) |
| 0.65  (-24.56,25.77) | 0.94  (-31.68,33.69) | 0.41  (-0.98,1.81) | 8.62  (-34.27,51.78) | 0.99  (-31.77,33.75) | -2.49  (-10.25,5.23) | -3.12  (-14.01,7.78) | 0.23  (-1.94,2.4) | **omega-3** |

**L.Network meta-analysis for total score of ADHD Rating Scale-Parent (network B) [ MD (95% CrI) ]**

| **Folic+MPH** | -1.11  (-41.8,39.85) | 2.23  (-43.71,48.62) | 4.03  (-32.7,41.3) | 0.38  (-40.38,41.84) | 1.7  (-39.73,43.61) | -4.5  (-49.14,41.09) | -1.23  (-71.27,69.38) | 6.23  (-37.07,49.66) | 2.96  (-34.26,40.52) |
| --- | --- | --- | --- | --- | --- | --- | --- | --- | --- |
| 1.11  (-39.85,41.8) | **Sweet almond syrup+Placebo** | 3.32  (-29.2,35.67) | 5.18  (-12.28,22.47) | 1.5  (-23.76,26.7) | 2.72  (-23.19,28.8) | -3.33  (-34.52,27.48) | -0.26  (-62.57,62.87) | 7.35  (-20.87,35.53) | 4.05  (-14.55,22.43) |
| -2.23  (-48.62,43.71) | -3.32  (-35.67,29.2) | **Acetyl-L**  **-carnitine+MPH** | 1.8  (-25.86,29.21) | -1.85  (-34.82,31.12) | -0.64  (-34.17,32.87) | -6.7  (-44.79,30.98) | -3.65  (-69.49,62.49) | 3.97  (-31.63,39.52) | 0.7  (-27.57,28.91) |
| -4.03  (-41.3,32.7) | -5.18  (-22.47,12.28) | -1.8  (-29.21,25.86) | **Placebo**  **+ MPH** | -3.67  (-21.84,14.62) | -2.36  (-21.69,16.96) | -8.47  (-34.46,17.53) | -5.35  (-64.98,55.07) | 2.2  (-20.21,24.49) | -1.1  (-7.37,5.16) |
| -0.38  (-41.84,40.38) | -1.5  (-26.7,23.76) | 1.85  (-31.12,34.82) | 3.67  (-14.62,21.84) | **Resveratrol**  **+ MPH** | 1.3  (-25.29,27.87) | -4.84  (-36.39,26.83) | -1.66  (-63.81,61.18) | 5.84  (-22.89,34.72) | 2.57  (-16.84,21.67) |
| -1.7  (-43.61,39.73) | -2.72  (-28.8,23.19) | 0.64  (-32.87,34.17) | 2.36  (-16.96,21.69) | -1.3  (-27.87,25.29) | **Ginkgo+MPH** | -6.04  (-38.69,26.38) | -3.09  (-65.63,60.43) | 4.55  (-24.95,34.19) | 1.25  (-18.99,21.63) |
| 4.5  (-41.09,49.14) | 3.33  (-27.48,34.52) | 6.7  (-30.98,44.79) | 8.47  (-17.53,34.46) | 4.84  (-26.83,36.39) | 6.04  (-26.38,38.69) | **Zinc+MPH** | 3.09  (-61.65,68.66) | 10.65  (-23.67,45.05) | 7.34  (-19.39,34.25) |
| 1.23  (-69.38,71.27) | 0.26  (-62.87,62.57) | 3.65  (-62.49,69.49) | 5.35  (-55.07,64.98) | 1.66  (-61.18,63.81) | 3.09  (-60.43,65.63) | -3.09  (-68.66,61.65) | **Vitamin D**  **+ MPH** | 7.55  (-56.88,71.1) | 4.27  (-56.69,64.22) |
| -6.23  (-49.66,37.07) | -7.35  (-35.53,20.87) | -3.97  (-39.52,31.63) | -2.2  (-24.49,20.21) | -5.84  (-34.72,22.89) | -4.55  (-34.19,24.95) | -10.65  (-45.05,23.67) | -7.55  (-71.1,56.88) | **omega-3+6**  **+ MPH** | -3.28  (-26.6,19.96) |
| -2.96  (-40.52,34.26) | -4.05  (-22.43,14.55) | -0.7  (-28.91,27.57) | 1.1  (-5.16,7.37) | -2.57  (-21.67,16.84) | -1.25  (-21.63,18.99) | -7.34  (-34.25,19.39) | -4.27  (-64.22,56.69) | 3.28  (-19.96,26.6) | **omega-3**  **+ MPH** |

**M.Network meta-analysis for attention score of ADHD Rating Scale-Teacher [ MD (95% CrI) ]**

| **Placebo** | -9.91  (-51.45,31.62) | -1.73  (-6.99,3.54) | -0.01  (-1.93,1.91) |
| --- | --- | --- | --- |
| 9.91  (-31.62,51.45) | **Pycnogenol** | 8.14  (-33.7,49.94) | 9.87  (-31.67,51.47) |
| 1.73  (-3.54,6.99) | -8.14  (-49.94,33.7) | **Vitamin D** | 1.71  (-3.85,7.31) |
| 0.01  (-1.91,1.93) | -9.87  (-51.47,31.67) | -1.71  (-7.31,3.85) | **omega-3** |

**N.Network meta-analysis for hyperactivity score of ADHD Rating Scale-Teacher [ MD (95% CrI) ]**

| **Placebo** | -7.47  (-52.9,38.08) | -2.85  (-8.65,3.01) | -0.11  (-2.16,1.92) |
| --- | --- | --- | --- |
| 7.47  (-38.08,52.9) | **Pycnogenol** | 4.57  (-41.29,50.59) | 7.35  (-38.25,52.73) |
| 2.85  (-3.01,8.65) | -4.57  (-50.59,41.29) | **Vitamin D** | 2.74  (-3.45,8.93) |
| 0.11  (-1.92,2.16) | -7.35  (-52.73,38.25) | -2.74  (-8.93,3.45) | **omega-3** |

**O.Network meta-analysis for total score of Rating Scale-Teacher (network A) [ MD (95% CrI) ]**

| **Placebo** | -4.54  (-13.61,4.6) | -0.73  (-2.52,1.05) | -0.06  (-1.67,1.54) |
| --- | --- | --- | --- |
| 4.54  (-4.6,13.61) | **Vitamin D** | 3.81  (-5.48,13.03) | 4.48  (-4.82,13.75) |
| 0.73  (-1.05,2.52) | -3.81  (-13.03,5.48) | **omega-3+6** | 0.67  (-1.72,3.07) |
| 0.06  (-1.54,1.67) | -4.48  (-13.75,4.82) | -0.67  (-3.07,1.72) | **omega-3** |

**P.Network meta-analysis for total score of ADHD Rating Scale-Teacher (network B) [ MD (95% CrI) ]**

| **Sweet almond syrup**  **+ Placebo** | -6.3  (-41.79,29.34) | -4.91  (-26.3,16.42) | -6.86  (-40.54,26.82) | -11.97  (-43.16,19.26) | -3.38  (-26.27,19.23) |
| --- | --- | --- | --- | --- | --- |
| 6.3  (-29.34,41.79) | **Acetyl-L-carnitine**  **+ MPH** | 1.39  (-26.84,29.51) | -0.61  (-38.81,37.47) | -5.66  (-42.04,30.32) | 2.88  (-26.35,32.05) |
| 4.91  (-16.42,26.3) | -1.39  (-29.51,26.84) | **Placebo+MPH** | -1.97  (-27.93,24) | -7.07  (-29.7,15.46) | 1.48  (-6.42,9.4) |
| 6.86  (-26.82,40.54) | 0.61  (-37.47,38.81) | 1.97  (-24,27.93) | **Resveratrol+MPH** | -5.2  (-39.31,29.63) | 3.42  (-23.66,30.69) |
| 11.97  (-19.26,43.16) | 5.66  (-30.32,42.04) | 7.07  (-15.46,29.7) | 5.2  (-29.63,39.31) | **Zinc+MPH** | 8.59  (-15.42,32.52) |
| 3.38  (-19.23,26.27) | -2.88  (-32.05,26.35) | -1.48  (-9.4,6.42) | -3.42  (-30.69,23.66) | -8.59  (-32.52,15.42) | **omega-3+MPH** |

**Q.Network meta-analysis for total score of ADHD Rating Scale-Teacher (networkC) [ MD (95% CrI) ]**

| **Quercetin** | -0.83  (-26.45,24.86) | 8.18  (-30.31,46.46) | -0.64  (-26.25,25.12) |
| --- | --- | --- | --- |
| 0.83  (-24.86,26.45) | **MPH** | 8.99  (-19.82,37.58) | 0.22  (-1.44,1.87) |
| -8.18  (-46.46,30.31) | -8.99  (-37.58,19.82) | **Ginkgo** | -8.77  (-37.53,20.14) |
| 0.64  (-25.12,26.25) | -0.22  (-1.87,1.44) | 8.77  (-20.14,37.53) | **Zinc** |

1. **Network meta-analysis for Clinical Global Impressions scale**

| **Acetyl-L-Carnitine** | -0.39  (-1.48,0.68) | 0.07  (-2.44,2.44) | -0.23  (-1.16,0.67) | -0.84  (-4.45,1.82) | 1.09  (-0.39,2.85) |
| --- | --- | --- | --- | --- | --- |
| 0.39  (-0.68,1.48) | **Resveratrol**  **+ MPH** | 0.46  (-1.95,2.74) | 0.16  (-0.42,0.75) | -0.44  (-4,2.15) | 1.47  (-0.19,3.14) |
| -0.07  (-2.44,2.44) | -0.46  (-2.74,1.95) | **MPH** | -0.31  (-2.5,2.04) | -0.89  (-4.39,1.6) | 1.01  (-0.51,3.05) |
| 0.23  (-0.67,1.16) | -0.16  (-0.75,0.42) | 0.31  (-2.04,2.5) | **Placebo** | -0.59  (-4.12,1.89) | 1.3  (-0.18,2.89) |
| 0.84  (-1.82,4.45) | 0.44  (-2.15,4) | 0.89  (-1.6,4.39) | 0.59  (-1.89,4.12) | **omega-3+6**  **+ MPH** | 1.9  (-0.02,5.27) |
| -1.09  (-2.85,0.39) | -1.47  (-3.14,-0.19) | -1.01  (-3.05,0.51) | -1.3  (-2.89,0.18) | -1.9  (-5.27,0.02) | **omega-3+6** |

Note: All results are presented as Lg[ OR (95% CrI) ]

**S.Network meta-analysis for Continuous Performance Test [ MD (95% CrI) ]**

| **Placebo** | 0.46  (-32.68,33.39) | 2.48  (-9.37,14.4) | -5.6  (-66.34,54.97) | 2.16  (-58.41,63.24) |
| --- | --- | --- | --- | --- |
| -0.46  (-33.39,32.68) | **Pycnogenol** | 2.1  (-32.98,37.19) | -5.92  (-74.81,63.17) | 1.82  (-67.22,71.28) |
| -2.48  (-14.4,9.37) | -2.1  (-37.19,32.98) | **Vitamin D** | -8.05  (-69.8,53.7) | -0.28  (-61.86,61.6) |
| 5.6  (-54.97,66.34) | 5.92  (-63.17,74.81) | 8.05  (-53.7,69.8) | **omega-3+6** | 7.77  (-78.3,93.74) |
| -2.16  (-63.24,58.41) | -1.82  (-71.28,67.22) | 0.28  (-61.6,61.86) | -7.77  (-93.74,78.3) | **omega-3** |

Note: Results in Table A and Table R are showed as Lg[OR (95% CrI)]. Results in Table B-Q and Table S are showed as MD (95% CrI), significant results are in bold and underscored. omega-3=omega-3 fatty acids, omega-6=omega-6 fatty acids, omega-3+6=omega-3 fatty acids plus omega-6 fatty acids, MPH=Methylphenidate, MD=mean difference, OR=odds ratio, CrI=credibility interval.
