# Supplementary material for: Safety and efficacy of antioxidant therapy in children and adolescents with attention deficit hyperactivity disorder: A systematic review and network meta-analysis
Source: PLoS One. 2024 Mar 28;19(3):e0296926. doi: 10.1371/journal.pone.0296926 (PMC10977718; doi:10.1371/journal.pone.0296926)
Supplement: S4 Table — (DOCX) [file pone.0296926.s005.docx]

Supplementary Material

## S5 Table. Probability ranking of SUCRA value.

**A.Probability ranking for safety of antioxidant therapy**

| **Intervention** | **SUCRA** | **Rank** | **Intervention** | **SUCRA** | **Rank** |
| --- | --- | --- | --- | --- | --- |
| MPH + Folic | 0.70 | 19 | Zinc+MPH | 0.88 | 22 |
| sweet almond syrup+Placebo | 0.31 | 7 | Zinc | 0.54 | 13 |
| Acetyl-L-carnitine+MPH | 0.32 | 6 | Vitamin D+MPH | 0.62 | 15 |
| Placebo + MPH | 0.68 | 18 | Vitamin D | 0.19 | 2 |
| Acetyl-L-carnitine | 0.32 | 8 | omega-3+Phosphatidylserine | 0.38 | 11 |
| Quercetin | 0.24 | 3 | Phosphatidylserine | 0.25 | 4 |
| Resveratrol+MPH | 0.92 | 23 | omega-3+6 + MPH | 0.65 | 16 |
| MPH | 0.46 | 12 | omega-3+6 | 0.33 | 9 |
| Placebo | 0.36 | 10 | omega-3+MPH | 0.85 | 21 |
| Pycnogenol | 0.64 | 17 | omega-3 | 0.29 | 5 |
| Ginkgo | 0.77 | 20 | omega-6 | 0.18 | 1 |
| Ginkgo+MPH | 0.61 | 14 |  |  |  |

**B.Probability ranking for attention score of Conners’ Parent Rating Scale**

| **Intervention** | **SUCRA** | **Rank** |
| --- | --- | --- |
| Acetyl-L-carnitine | 0.47 | 3 |
| Placebo | 0.54 | 5 |
| Pycnogenol | 0.59 | 6 |
| Phosphatidylserine + omega-3 | 0.38 | 2 |
| omega-3+6 | 0.48 | 4 |
| omega-3 | 0.35 | 1 |
| omega-6 | 0.67 | 7 |

**C.Probability ranking for hyperactivity score of Conners’ Parent Rating Scale**

| **Intervention** | **SUCRA** | **Rank** |
| --- | --- | --- |
| Acetyl-L-carnitine | 0.58 | 6 |
| Placebo | 0.61 | 7 |
| Pycnogenol | 0.36 | 1 |
| Phosphatidylserine + omega-3 | 0.42 | 2 |
| omega-3+6 | 0.53 | 4 |
| omega-3 | 0.44 | 3 |
| omega-6 | 0.55 | 5 |

**D.Probability ranking for total score of Conners’ Parent Rating Scale(network A)**

| **Intervention** | **SUCRA** | **Rank** |
| --- | --- | --- |
| Acetyl-L-carnitine | 0.56 | 5 |
| Placebo | 0.59 | 7 |
| Vitamin D | 0.27 | 1 |
| Phosphatidylserine + omega-3 | 0.39 | 2 |
| omega-3+6 | 0.53 | 3 |
| omega-3 | 0.54 | 4 |
| omega-6 | 0.57 | 6 |

**E.Probability ranking for total score of Conners’ Parent Rating Scale(network B)**

| **Intervention** | **SUCRA** | **Rank** |
| --- | --- | --- |
| Folic + MPH | 0.52 | 3 |
| Placebo + MPH | 0.54 | 5 |
| Zinc + MPH | 0.43 | 1 |
| Vitamin D + MPH | 0.46 | 2 |
| omega-3 + MPH | 0.53 | 4 |

**F.Probability ranking for attentionl score of Conners’ Teacher Rating Scale**

| **Intervention** | **SUCRA** | **Rank** |
| --- | --- | --- |
| Acetyl-L-carnitine | 0.58 | 4 |
| Placebo | 0.62 | 5 |
| Pycnogenol | 0.32 | 1 |
| Phosphatidylserine + omega-3 | 0.47 | 2 |
| omega-3 | 0.50 | 3 |

**G.Probability ranking for hyperactivity score of Conners’ Teacher Rating Scale**

| **Intervention** | **SUCRA** | **Rank** |
| --- | --- | --- |
| Acetyl-L-carnitine | 0.71 | 7 |
| MPH | 0.29 | 2 |
| Placebo | 0.69 | 6 |
| Pycnogenol | 0.55 | 4 |
| Zinc | 0.30 | 3 |
| Phosphatidylserine+omega-3 | 0.26 | 1 |
| omega-3 | 0.55 | 5 |

**H.Probability ranking for total score of Conners’ Teacher Rating Scale**

| **Intervention** | **SUCRA** | **Rank** |
| --- | --- | --- |
| Acetyl-L-carnitine | 0.57 | 5 |
| MPH | 0.36 | 2 |
| Placebo | 0.71 | 7 |
| Zinc | 0.34 | 1 |
| Phosphatidylserine+omega-3 | 0.44 | 4 |
| omega-3+6 | 0.42 | 3 |
| omega-3 | 0.63 | 6 |

**I.Probability ranking for attention score of ADHD Rating Scale-Parent**

| **Intervention** | **SUCRA** | **Rank** | **Intervention** | **SUCRA** | **Rank** |
| --- | --- | --- | --- | --- | --- |
| Folic+MPH | 0.54 | 14 | Ginkgo+MPH | 0.46 | 6 |
| Sweet almond syrup+Placebo | 0.51 | 10 | Zinc | 0.44 | 4 |
| MPH+Placebo | 0.55 | 15 | Vitamin D+MPH | 0.40 | 2 |
| Quercetin | 0.52 | 11 | Vitamin D | 0.43 | 3 |
| Resveratrol+MPH | 0.48 | 7 | Phosphatidylserine | 0.39 | 1 |
| MPH | 0.45 | 5 | omega-3+6 + MPH | 0.49 | 8 |
| Placebo | 0.53 | 12 | omega-3+6 | 0.50 | 9 |
| Pycnogenol | 0.58 | 17 | omega-3+MPH | 0.53 | 13 |
| Ginkgo | 0.61 | 18 | omega-3 | 0.57 | 16 |

**J.Probability ranking for hyperactivity score of ADHD Rating Scale-Parent**

| **Intervention** | **SUCRA** | **Rank** | **Intervention** | **SUCRA** | **Rank** |
| --- | --- | --- | --- | --- | --- |
| Folic+MPH | 0.32 | 4 | Ginkgo+MPH | 0.31 | 3 |
| Sweet almond syrup+Placebo | 0.33 | 5 | Zinc | 0.54 | 9 |
| MPH+Placebo | 0.30 | 2 | Vitamin D+MPH | 0.43 | 8 |
| Quercetin | 0.65 | 14 | Vitamin D | 0.62 | 13 |
| Resveratrol+MPH | 0.24 | 1 | Phosphatidylserine | 0.60 | 12 |
| MPH | 0.55 | 10 | omega-3+6 + MPH | 0.36 | 6 |
| Placebo | 0.70 | 18 | omega-3+6 | 0.66 | 15 |
| Pycnogenol | 0.57 | 11 | omega-3+MPH | 0.38 | 7 |
| Ginkgo | 0.69 | 16 | omega-3 | 0.67 | 17 |

**K.Probability ranking for total score of ADHD Rating Scale-Parent (network A)**

| **Intervention** | **SUCRA** | **Rank** |
| --- | --- | --- |
| Quercetin | 0.51 | 5 |
| MPH | 0.50 | 4 |
| Placebo | 0.58 | 8 |
| Ginkgo | 0.67 | 9 |
| Zinc | 0.50 | 6 |
| Vitamin D | 0.35 | 2 |
| Phosphatidylserine | 0.34 | 1 |
| omega-3+6 | 0.53 | 7 |
| omega-3 | 0.49 | 3 |

**L.Probability ranking for total score of ADHD Rating Scale-Parent (network B)**

| **Intervention** | **SUCRA** | **Rank** |
| --- | --- | --- |
| Folic+MPH | 0.47 | 5 |
| Sweet almond syrup + Placebo | 0.41 | 2 |
| Acetyl-L-carnitine+MPH | 0.52 | 7 |
| Placebo+MPH | 0.60 | 9 |
| Resveratrol+MPH | 0.46 | 3 |
| Ginkgo+MPH | 0.50 | 6 |
| Zinc+MPH | 0.35 | 1 |
| Vitamin D+MPH | 0.46 | 4 |
| omega-3+6 + MPH | 0.62 | 10 |
| omega-3+MPH | 0.54 | 8 |

**M.Probability ranking for attention score of ADHD Rating Scale-Teacher**

| **Intervention** | **SUCRA** | **Rank** |
| --- | --- | --- |
| Placebo | 0.64 | 4 |
| Pycnogenol | 0.32 | 1 |
| Vitamin D | 0.39 | 2 |
| omega-3 | 0.63 | 3 |

**N.Probability ranking for hyperactivity score of ADHD Rating Scale-Teacher**

| **Intervention** | **SUCRA** | **Rank** |
| --- | --- | --- |
| Placebo | 0.66 | 4 |
| Pycnogenol | 0.39 | 2 |
| Vitamin D | 0.31 | 1 |
| omega-3 | 0.62 | 3 |

**O.Probability ranking for total score of ADHD Rating Scale-Teacher(network A)**

| **Intervention** | **SUCRA** | **Rank** |
| --- | --- | --- |
| Placebo | 0.72 | 4 |
| Vitamin D | 0.18 | 1 |
| omega-3+6 | 0.43 | 2 |
| omega-3 | 0.66 | 3 |

**P.Probability ranking for total score of ADHD Rating Scale-Teacher(network B)**

| **Intervention** | **SUCRA** | **Rank** |
| --- | --- | --- |
| sweet almond syrup +Placebo | 0.67 | 6 |
| Acetyl-L-carnitine+MPH | 0.47 | 3 |
| Placebo+MPH | 0.50 | 4 |
| Resveratrol+MPH | 0.45 | 2 |
| Zinc+MPH | 0.29 | 1 |
| omega-3+MPH | 0.59 | 5 |

**Q.Probability ranking for total score of ADHD Rating Scale-Teacher(networkC)**

| **Intervention** | **SUCRA** | **Rank** |
| --- | --- | --- |
| Quercetin | 0.46 | 3 |
| MPH | 0.38 | 1 |
| Ginkgo | 0.70 | 4 |
| Zinc | 0.44 | 2 |

**R.Probability ranking for Clinical Global Impressions scale**

| **Intervention** | **SUCRA** | **Rank** |
| --- | --- | --- |
| Acetyl-L-carnitine | 0.55 | 2 |
| Quercetin | 0.30 | 5 |
| MPH | 0.52 | 3 |
| Placebo | 0.41 | 4 |
| omega-3+6 + MPH | 0.24 | 6 |
| omega-3+6 | 0.95 | 1 |

**S.Probability ranking for Continuous Performance Test**

| **Intervention** | **SUCRA** | **Rank** |
| --- | --- | --- |
| Placebo | 0.46 | 2 |
| Pycnogenol | 0.50 | 3 |
| Vitamin D | 0.57 | 5 |
| omega-3+6 | 0.42 | 1 |
| omega-3 | 0.52 | 4 |

Note: The SUCRA value ranges from 0 to 1. The closer the value is to 0, the lower the probability of an event, while the closer the value is to 1, the higher the probability. omega-3=omega-3 fatty acids, omega-6=omega-6 fatty acids, omega-3+6=omega-3 fatty acids + omega-6 fatty acids, MPH=Methylphenidate.
