# Supplementary material for: Safety and efficacy of antioxidant therapy in children and adolescents with attention deficit hyperactivity disorder: A systematic review and network meta-analysis
Source: PLoS One. 2024 Mar 28;19(3):e0296926. doi: 10.1371/journal.pone.0296926 (PMC10977718; doi:10.1371/journal.pone.0296926)
Supplement: S5 Table — (DOCX) [file pone.0296926.s006.docx]

Supplementary Material

## S6 Table. Risk of bias for included studies.

| **Stydy, year** | **Random sequence generation (selection bias)** | **Allocation concealment (selection bias)** | **Blinding of participants and personnel (performance bias)** | **Blinding of outcome assessment (detection bias)** | **Incomplete outcome data (attrition bias)** | **Selective reporting (reporting bias)** | **Other bias** |
| --- | --- | --- | --- | --- | --- | --- | --- |
| Rafeiy Torghabeh et al, 2020 | low | low | low | low | high | low | unclear |
| Motaharifard et al, 2019 | low | low | low | low | high | low | unclear |
| Akhondzadeh et al, 2005 | low | low | low | low | low | low | unclear |
| Weber et al, 2008 | low | low | unclear | low | low | low | unclear |
| Ghanizadeh et al, 2013 | unclear | unclear | unclear | unclear | low | low | unclear |
| Riahi et al, 2018 | low | low | unclear | unclear | low | low | unclear |
| Salehi et al, 2009 | low | low | low | low | low | low | unclear |
| Shakibaei et al, 2015 | low | low | unclear | low | low | low | unclear |
| Abbasi et al, 2013 | low | low | low | low | low | low | unclear |
| Arnold et al, 2007 | unclear | unclear | unclear | unclear | high | low | unclear |
| Akhondzadeh et al, 2004 | low | low | low | low | low | low | unclear |
| Arnold et al, 2011 | unclear | unclear | unclear | unclear | low | low | unclear |
| Bilici et al, 2004 | unclear | low | unclear | unclear | low | low | unclear |
| Noorazar et al, 2020 | unclear | low | unclear | unclear | low | low | unclear |
| Hsu et al, 2021 | low | unclear | unclear | unclear | low | low | unclear |
| Trebaticka et al, 2006 | low | low | unclear | unclear | high | low | unclear |
| Manor et al, 2012 | low | low | unclear | unclear | low | low | unclear |
| Hirayama et al, 2014 | unclear | low | unclear | unclear | low | low | unclear |
| Vaisman et al, 2008 | low | unclear | unclear | unclear | low | low | unclear |
| Dehbokri et al, 2018 | unclear | low | unclear | unclear | high | low | unclear |
| Elshorbagy et al, 2018 | unclear | low | unclear | unclear | low | low | unclear |
| Mohammadpour et al, 2016 | low | low | unclear | unclear | low | low | unclear |
| Naeini et al, 2019 | unclear | unclear | unclear | unclear | low | low | unclear |
| Rahmani et al, 2022 | unclear | unclear | unclear | unclear | high | low | unclear |
| Hemamy et al, 2020 | low | low | low | low | low | low | unclear |
| Assareh et al, 2012 | low | low | low | low | low | low | unclear |
| Barragán et al, 2014 | unclear | unclear | unclear | unclear | low | low | unclear |
| Carucci et al, 2021 | low | low | low | low | low | low | unclear |
| Döpfner et al, 2019 | low | low | low | low | high | low | unclear |
| Johnson et al, 2012 | unclear | low | unclear | unclear | low | low | unclear |
| Stevens et al, 2003 | unclear | low | unclear | unclear | low | low | unclear |
| Matsudaira et al, 2015 | unclear | low | unclear | unclear | high | low | unclear |
| Cornu et al, 2017 | low | low | low | unclear | high | low | unclear |
| Behdani et al, 2013 | low | low | unclear | low | low | low | unclear |
| Bélanger et al, 2009 | unclear | unclear | unclear | unclear | low | low | unclear |
| Chang et al, 2019 | low | unclear | low | low | low | low | unclear |
| Crippa et al, 2018 | low | low | low | low | low | low | unclear |
| Kean et al, 2016 | low | low | low | unclear | high | low | unclear |
| Dubnov-Raz et al, 2014 | unclear | low | low | low | low | low | unclear |
| Gustafsson et al, 2010 | low | unclear | unclear | unclear | low | low | unclear |
| Hariri M et al, 2012 | low | low | unclear | unclear | low | low | unclear |
| Milte et al, 2011 | unclear | unclear | low | low | low | low | unclear |
| Moghaddam et al, 2017 | low | low | unclear | unclear | low | low | unclear |
| Mohammadzadeh et al, 2019 | low | low | unclear | unclear | low | low | unclear |
| Raz et al , 2009 | unclear | low | low | low | low | low | unclear |
| Rodríguez et al, 2019 | low | low | low | low | low | low | unclear |
| Salehi et al, 2015 | low | low | unclear | unclear | low | low | unclear |
| Widenhorn-Müller et al, 2014 | low | unclear | low | low | low | low | unclear |

| **Study, Year**  **Country** | **Entry** | **Risk level** | **Judgement criteria** |
| --- | --- | --- | --- |
| Rafeiy Torghabeh et al, 2020  Iran | Random sequence generation (selection bias) | Low | Patients were randomly allocated to treatment groups by a computer-generated random queue. |
|  | Allocation concealment (selection bias) | Low | The allocation was carried out using sealed opaque envelopes with an aluminum foil inside to ensure that the contents are not detectable even in intense light. |
|  | Blinding of participants and personnel (performance bias) | Low | The medication distributors, participants and their guardians, research coordinators, and the outcome assessors were all blinded to allocation. |
|  | Blinding of outcome assessment (detection bias) | Low | The medication distributors, participants and their guardians, research coordinators, and the outcome assessors were all blinded to allocation. |
|  | Incomplete outcome data (attrition bias) | high | There are missed patients in this article without clearly reasons. |
|  | Selective reporting (reporting bias) | Low | Outcome indicators are reported according to the experimental design. |
|  | Other bias | unclear | - |
| Motaharifard et al, 2019  Iran | Random sequence generation (selection bias) | Low | The patients included in the study were randomized in a 1:1 ratiousing a computer-generated code. |
|  | Allocation concealment (selection bias) | Low | The person who administered the medications, the assessor, and patients along with their parents were blinded to the allocation of study groups. |
|  | Blinding of participants and personnel (performance bias) | Low | The person who administered the medications and patients along with their parents were blinded. |
|  | Blinding of outcome assessment (detection bias) | Low | The person who administered the assessor were blinded to the allocation of study groups. |
|  | Incomplete outcome data (attrition bias) | high | Some patients discontinued in this article without specific reasons. |
|  | Selective reporting (reporting bias) | Low | Outcome indicators are reported according to the experimental design. |
|  | Other bias | unclear | - |
| Akhondzadeh et al, 2005  Iran | Random sequence generation (selection bias) | Low | Patients were randomized to receive tablets in a 1:1 ratio using a computer generated code. |
|  | Allocation concealment (selection bias) | Low | The assignments were kept in sealed, opaque envelopes until the point of allocation. |
|  | Blinding of participants and personnel (performance bias) | Low | Throughout the study the person administrated the medications and patients were blind to assignments. |
|  | Blinding of outcome assessment (detection bias) | Low | Throughout the study the rater were blind to assignments. |
|  | Incomplete outcome data (attrition bias) | Low | There are no missed visits in this article. |
|  | Selective reporting (reporting bias) | Low | The outcome indicators have been specified in the experimental design. |
|  | Other bias | unclear | - |
| Weber et al, 2008  America | Random sequence generation (selection bias) | Low | An independent data manager created the randomization sequence allowing the principal investigator and recruitment staff to remain blinded to the randomization code until the database was locked. |
|  | Allocation concealment (selection bias) | Low | An independent pharmacy technician placed the study medication in consecutively numbered bottles that were identical in appearance. |
|  | Blinding of participants and personnel (performance bias) | unclear | - |
|  | Blinding of outcome assessment (detection bias) | Low | The principal investigator, blinded to treatment assignment, administered the outcom at each study visit. |
|  | Incomplete outcome data (attrition bias) | Low | Patients who missed the interview all gave reasons for missing the interview. |
|  | Selective reporting (reporting bias) | Low | Outcome indicators are reported according to the experimental design. |
|  | Other bias | unclear | - |
| Ghanizadeh et al, 2013  Iran | Random sequence generation (selection bias) | unclear | The article only mentions random allocation, but does not describe the method of random allocation. |
|  | Allocation concealment (selection bias) | unclear | - |
|  | Blinding of participants and personnel (performance bias) | unclear | - |
|  | Blinding of outcome assessment (detection bias) | unclear | - |
|  | Incomplete outcome data (attrition bias) | low | Patients who missed the interview all gave reasons for missing the interview |
|  | Selective reporting (reporting bias) | low | Outcome indicators are reported according to the experimental design. |
|  | Other bias | unclear | - |
| Riahi et al, 2018  Iran | Random sequence generation (selection bias) | Low | All the study subjects were randomly assigned at a 1:1 ratio using a computergenerated code. |
|  | Allocation concealment (selection bias) | Low | The assignments were kept in sealed, opaque envelopes until the point of allocation. |
|  | Blinding of participants and personnel (performance bias) | unclear | - |
|  | Blinding of outcome assessment (detection bias) | unclear | - |
|  | Incomplete outcome data (attrition bias) | Low | Patients who missed the interview all gave reasons for missing the interview. |
|  | Selective reporting (reporting bias) | Low | Outcome indicators are reported according to the experimental design. |
|  | Other bias | unclear | - |
| Salehi et al, 2009  Iran | Random sequence generation (selection bias) | Low | Patients were randomized to receive the medicine in a 1:1 ratio using a computer-generated code. |
|  | Allocation concealment (selection bias) | Low | Both tablets were encapsulated and were identical， The assignments were kept in sealed, opaque envelopes until the point of analysis of data. |
|  | Blinding of participants and personnel (performance bias) | Low | Throughout the study the person who administrated the medications and the patients along with their parents were blind to group assignments. |
|  | Blinding of outcome assessment (detection bias) | Low | Throughout the study the rater were blind to group assignments. |
|  | Incomplete outcome data (attrition bias) | Low | There are no missed visits in this article. |
|  | Selective reporting (reporting bias) | Low | Outcome indicators are reported according to the experimental design. |
|  | Other bias | unclear | - |
| Shakibaei et al, 2015  Iran | Random sequence generation (selection bias) | Low | Randomization was done using the random allocation software producing a table with two alphabets which were randomly distributed among consecutive numbers. |
|  | Allocation concealment (selection bias) | Low | The assignments were kept in sealed and opaque envelopes until the point of data analysis. |
|  | Blinding of participants and personnel (performance bias) | unclear | - |
|  | Blinding of outcome assessment (detection bias) | Low | The outcome assessor was not aware about the study arms. |
|  | Incomplete outcome data (attrition bias) | Low | Patients who discontinued all gave reasons for missing the trail. |
|  | Selective reporting (reporting bias) | Low | Outcome indicators are reported according to the experimental design. |
|  | Other bias | unclear | - |
| Abbasi et al, 2013  Iran | Random sequence generation (selection bias) | Low | Patients were randomized to receive the medicine in a 1:1 ratio using a computer-generated code. |
|  | Allocation concealment (selection bias) | Low | The assignments were kept in sealed, opaque envelopes until the point of data analysis. |
|  | Blinding of participants and personnel (performance bias) | Low | Throughout the study, the person who administrated the medications and the patients along with their parents were blind to group assignments. |
|  | Blinding of outcome assessment (detection bias) | Low | Throughout the study, the rater were blind to group assignments. |
|  | Incomplete outcome data (attrition bias) | Low | Patients who missed the interview all gave reasons for missing the interview. |
|  | Selective reporting (reporting bias) | Low | Outcome indicators are reported according to the experimental design. |
|  | Other bias | unclear | - |
| Arnold et al, 2007  American | Random sequence generation (selection bias) | unclear | The article only mentions random allocation, but does not describe the method of random allocation. |
|  | Allocation concealment (selection bias) | unclear | - |
|  | Blinding of participants and personnel (performance bias) | unclear | - |
|  | Blinding of outcome assessment (detection bias) | unclear | - |
|  | Incomplete outcome data (attrition bias) | high | Some patients discontinued in this article without specific reasons. |
|  | Selective reporting (reporting bias) | Low | Outcome indicators are reported according to the experimental design. |
|  | Other bias | unclear | - |
| Akhondzadeh et al, 2004  Iran | Random sequence generation (selection bias) | Low | Patients were randomized to receive the medicine in a 1: 1 ratio using a computer generated code. |
|  | Allocation concealment (selection bias) | Low | The assignments were kept in sealed, opaque envelopes until the point of allocation. |
|  | Blinding of participants and personnel (performance bias) | Low | Throughout the study the person who administrated the medications and patients were blind to assignments. |
|  | Blinding of outcome assessment (detection bias) | Low | Throughout the study the rater were blind to assignments. |
|  | Incomplete outcome data (attrition bias) | Low | There are no missed visits in this article. |
|  | Selective reporting (reporting bias) | Low | Outcome indicators are reported according to the experimental design. |
|  | Other bias | unclear | - |
| Arnold et al, 2011  America | Random sequence generation (selection bias) | unclear | The article only mentions random allocation, but does not describe the method of random allocation. |
|  | Allocation concealment (selection bias) | unclear | - |
|  | Blinding of participants and personnel (performance bias) | unclear | - |
|  | Blinding of outcome assessment (detection bias) | unclear | - |
|  | Incomplete outcome data (attrition bias) | Low | There are no missed patients in the phase that we needed. |
|  | Selective reporting (reporting bias) | Low | Outcome indicators are reported according to the experimental design. |
|  | Other bias | unclear | - |
| Bilici et al, 2004  Turkey | Random sequence generation (selection bias) | unclear | The article only mentions random allocation, but does not describe the method of random allocation. |
|  | Allocation concealment (selection bias) | Low | Study drug materials for both treatment groups were identical in appearance. |
|  | Blinding of participants and personnel (performance bias) | unclear | - |
|  | Blinding of outcome assessment (detection bias) | unclear | - |
|  | Incomplete outcome data (attrition bias) | Low | Patients who missed the interview all gave reasons for missing the interview. |
|  | Selective reporting (reporting bias) | Low | Outcome indicators are reported according to the experimental design. |
|  | Other bias | unclear | - |
| Noorazar et al, 2020  Iran | Random sequence generation (selection bias) | unclear | The article only mentions random allocation, but does not describe the method of random allocation. |
|  | Allocation concealment (selection bias) | Low | The placebo and zinc syrups were prepared and labeled by Razak Company and in terms of appearance were quite similar. |
|  | Blinding of participants and personnel (performance bias) | unclear | - |
|  | Blinding of outcome assessment (detection bias) | unclear | - |
|  | Incomplete outcome data (attrition bias) | Low | There are no missed visits in this article. |
|  | Selective reporting (reporting bias) | Low | Outcome indicators are reported according to the experimental design. |
|  | Other bias | unclear | - |
| Hsu et al, 2021  China | Random sequence generation (selection bias) | Low | The randomization was performed by drawing lots. |
|  | Allocation concealment (selection bias) | unclear | - |
|  | Blinding of participants and personnel (performance bias) | unclear | - |
|  | Blinding of outcome assessment (detection bias) | unclear | - |
|  | Incomplete outcome data (attrition bias) | Low | Patients who missed the interview all gave reasons for missing the interview. |
|  | Selective reporting (reporting bias) | Low | Outcome indicators are reported according to the experimental design. |
|  | Other bias | unclear | - |
| Trebaticka et al, 2006  Slovakia Republic | Random sequence generation (selection bias) | Low | StatDirect2.3.7(computer) was used for the randomization. |
|  | Allocation concealment (selection bias) | Low | Medicine and placebo were identical shape and appearance and the same number. |
|  | Blinding of participants and personnel (performance bias) | unclear | - |
|  | Blinding of outcome assessment (detection bias) | unclear | - |
|  | Incomplete outcome data (attrition bias) | high | Some patients discontinued in this article without specific reasons. |
|  | Selective reporting (reporting bias) | Low | Outcome indicators are reported according to the experimental design. |
|  | Other bias | unclear | - |
| Manor et al, 2012  Israel | Random sequence generation (selection bias) | Low | A web-based random allocation procedure was used to enhance the concealment and ease of use. |
|  | Allocation concealment (selection bias) | Low | During the double-blind phase participants received four identical-looking capsule. |
|  | Blinding of participants and personnel (performance bias) | unclear | - |
|  | Blinding of outcome assessment (detection bias) | unclear | - |
|  | Incomplete outcome data (attrition bias) | Low | Patients who missed the interview all gave reasons for missing the interview. |
|  | Selective reporting (reporting bias) | Low | Outcome indicators are reported according to the experimental design. |
|  | Other bias | unclear | - |
| Hirayama et al, 2014  Japan | Random sequence generation (selection bias) | unclear | The article only mentions random allocation, but does not describe the method of random allocation. |
|  | Allocation concealment (selection bias) | Low | The medicine were matched in taste and appearance. |
|  | Blinding of participants and personnel (performance bias) | unclear | - |
|  | Blinding of outcome assessment (detection bias) | unclear | - |
|  | Incomplete outcome data (attrition bias) | Low | Patients who missed the interview all gave reasons for missing the interview. |
|  | Selective reporting (reporting bias) | Low | Outcome indicators are reported according to the experimental design. |
|  | Other bias | unclear | - |
| Vaisman et al, 2008  Israel | Random sequence generation (selection bias) | Low | The randomization sequence was a block randomization process. |
|  | Allocation concealment (selection bias) | unclear | - |
|  | Blinding of participants and personnel (performance bias) | unclear | - |
|  | Blinding of outcome assessment (detection bias) | unclear | - |
|  | Incomplete outcome data (attrition bias) | Low | Patients who missed the interview all gave reasons for missing the interview. |
|  | Selective reporting (reporting bias) | Low | Outcome indicators are reported according to the experimental design. |
|  | Other bias | unclear | - |
| Dehbokri et al, 2018  Iran | Random sequence generation (selection bias) | unclear | The article only mentions random allocation, but does not describe the method of random allocation. |
|  | Allocation concealment (selection bias) | Low | A local company manufactured both intervention and placebo pearls in a same shape and color. |
|  | Blinding of participants and personnel (performance bias) | unclear | - |
|  | Blinding of outcome assessment (detection bias) | unclear | - |
|  | Incomplete outcome data (attrition bias) | high | Some patients discontinued in this article without specific reasons. |
|  | Selective reporting (reporting bias) | Low | Outcome indicators are reported according to the experimental design. |
|  | Other bias | unclear | - |
| Elshorbagy et al, 2018  Egypt | Random sequence generation (selection bias) | unclear | The article only mentions random allocation, but does not describe the method of random allocation. |
|  | Allocation concealment (selection bias) | Low | Intervention and placebo were identical in appearance to guarantee blinding. |
|  | Blinding of participants and personnel (performance bias) | unclear | - |
|  | Blinding of outcome assessment (detection bias) | unclear | - |
|  | Incomplete outcome data (attrition bias) | Low | Patients who missed the interview all gave reasons for missing the interview. |
|  | Selective reporting (reporting bias) | Low | Outcome indicators are reported according to the experimental design. |
|  | Other bias | unclear | - |
| Mohammadpour et al, 2016  Iran | Random sequence generation (selection bias) | Low | Participants were stratified by gender and randomly assigned into two groups by permuted-block randomization. |
|  | Allocation concealment (selection bias) | Low | Intervention and placebo were identical in appearance to guarantee blinding. |
|  | Blinding of participants and personnel (performance bias) | unclear | - |
|  | Blinding of outcome assessment (detection bias) | unclear | - |
|  | Incomplete outcome data (attrition bias) | Low | Patients who missed the interview all gave reasons for missing the interview. |
|  | Selective reporting (reporting bias) | Low | Outcome indicators are reported according to the experimental design. |
|  | Other bias | unclear | - |
| Naeini et al, 2019  Iran | Random sequence generation (selection bias) | unclear | The article only mentions random allocation, but does not describe the method of random allocation. |
|  | Allocation concealment (selection bias) | unclear | - |
|  | Blinding of participants and personnel (performance bias) | unclear | - |
|  | Blinding of outcome assessment (detection bias) | unclear | - |
|  | Incomplete outcome data (attrition bias) | Low | Patients who missed the interview all gave reasons for missing the interview. |
|  | Selective reporting (reporting bias) | Low | Outcome indicators are reported according to the experimental design. |
|  | Other bias | unclear | - |
| Rahmani et al, 2022  Iran | Random sequence generation (selection bias) | unclear | The article only mentions random allocation, but does not describe the method of random allocation. |
|  | Allocation concealment (selection bias) | unclear | - |
|  | Blinding of participants and personnel (performance bias) | unclear | - |
|  | Blinding of outcome assessment (detection bias) | unclear | - |
|  | Incomplete outcome data (attrition bias) | high | Some patients discontinued in this article without specific reasons. |
|  | Selective reporting (reporting bias) | Low | Outcome indicators are reported according to the experimental design. |
|  | Other bias | unclear | - |
| Hemamy et al, 2020  Iran | Random sequence generation (selection bias) | Low | An independent person made random allocation cards using computer‑generated sequence and used sequentially numbered. |
|  | Allocation concealment (selection bias) | Low | Medicine were sealed, opaque envelopes to conceal the allocation. |
|  | Blinding of participants and personnel (performance bias) | Low | Neither the researcher nor the participants were aware of the two allocated groups. |
|  | Blinding of outcome assessment (detection bias) | Low | Neither the researcher nor the participants were aware of the two allocated groups. |
|  | Incomplete outcome data (attrition bias) | Low | There are no missed visits in this article. |
|  | Selective reporting (reporting bias) | Low | Outcome indicators are reported according to the experimental design. |
|  | Other bias | unclear | - |
| Assareh et al, 2012  Iran | Random sequence generation (selection bias) | Low | The sample was randomly selected based on random numbers table from the outpatient clinic of child psychiatry. |
|  | Allocation concealment (selection bias) | Low | The medicine were identical capsules from the same company with the same order. |
|  | Blinding of participants and personnel (performance bias) | Low | Patients and investigator were blind about the study groups (treatment or placebo). |
|  | Blinding of outcome assessment (detection bias) | Low | Patients and investigator were blind about the study groups (treatment or placebo). |
|  | Incomplete outcome data (attrition bias) | Low | There are no missed visits in this article. |
|  | Selective reporting (reporting bias) | Low | Outcome indicators are reported according to the experimental design. |
|  | Other bias | unclear | - |
| Barragán et al, 2014  Mexico | Random sequence generation (selection bias) | unclear | The article only mentions random allocation, but does not describe the method of random allocation. |
|  | Allocation concealment (selection bias) | unclear | - |
|  | Blinding of participants and personnel (performance bias) | unclear | - |
|  | Blinding of outcome assessment (detection bias) | unclear | - |
|  | Incomplete outcome data (attrition bias) | Low | Patients who missed the interview all gave reasons for missing the interview |
|  | Selective reporting (reporting bias) | Low | Outcome indicators are reported according to the experimental design. |
|  | Other bias | unclear | - |
| Carucci et al, 2021  Italy | Random sequence generation (selection bias) | Low | Random allocation sequence was generated by the Clinical Research Organization (CRO) based at the Clinical Pharmacology Unit. |
|  | Allocation concealment (selection bias) | Low | The medicine capsules matched identical in touch, smell, and size. |
|  | Blinding of participants and personnel (performance bias) | Low | All participants and investigators were blinded. |
|  | Blinding of outcome assessment (detection bias) | Low | All participants and investigators were blinded. |
|  | Incomplete outcome data (attrition bias) | Low | Patients who missed the interview all gave reasons for missing the interview |
|  | Selective reporting (reporting bias) | Low | Outcome indicators are reported according to the experimental design. |
|  | Other bias | unclear | - |
| Döpfner et al, 2019  Germany | Random sequence generation (selection bias) | Low | Participating children were randomly assigned to group using computerized block randomization. |
|  | Allocation concealment (selection bias) | Low | The medicine appeared in identically looking capsules in envelopes that did not indicate. |
|  | Blinding of participants and personnel (performance bias) | Low | The participants were blind regarding the study condition. |
|  | Blinding of outcome assessment (detection bias) | Low | The outcome assessor were blind regarding the study condition. |
|  | Incomplete outcome data (attrition bias) | high | Some patients discontinued in this article without specific reasons |
|  | Selective reporting (reporting bias) | Low | Outcome indicators are reported according to the experimental design. |
|  | Other bias | unclear | - |
| Johnson et al, 2012  Sweden | Random sequence generation (selection bias) | unclear | The article only mentions random allocation, but does not describe the method of random allocation. |
|  | Allocation concealment (selection bias) | Low | The manufacturer of medicine provided consecutively numbered identical bottles in random order according to a code list that was not accessible to the investigators. |
|  | Blinding of participants and personnel (performance bias) | unclear | - |
|  | Blinding of outcome assessment (detection bias) | unclear | - |
|  | Incomplete outcome data (attrition bias) | Low | Patients who missed the interview all gave reasons for missing the interview |
|  | Selective reporting (reporting bias) | Low | Outcome indicators are reported according to the experimental design. |
|  | Other bias | unclear | - |
| Stevens et al, 2003  Indiana | Random sequence generation (selection bias) | unclear | The article only mentions random allocation, but does not describe the method of random allocation. |
|  | Allocation concealment (selection bias) | Low | The odor and appearance of the medicine capsules were comparable. |
|  | Blinding of participants and personnel (performance bias) | unclear | - |
|  | Blinding of outcome assessment (detection bias) | unclear | - |
|  | Incomplete outcome data (attrition bias) | Low | Patients who missed the interview all gave reasons for missing the interview |
|  | Selective reporting (reporting bias) | Low | Outcome indicators are reported according to the experimental design. |
|  | Other bias | unclear | - |
| Matsudaira et al, 2015  United Kingdom | Random sequence generation (selection bias) | unclear | The article only mentions random allocation, but does not describe the method of random allocation. |
|  | Allocation concealment (selection bias) | Low | Medicine provided in four identical bottles labeled with an identifying code and in compliance with good manufacturing process. |
|  | Blinding of participants and personnel (performance bias) | unclear | - |
|  | Blinding of outcome assessment (detection bias) | unclear | - |
|  | Incomplete outcome data (attrition bias) | high | Some patients discontinued in this article without specific reasons. |
|  | Selective reporting (reporting bias) | Low | Outcome indicators are reported according to the experimental design. |
|  | Other bias | unclear | - |
| Cornu et al, 2017  France | Random sequence generation (selection bias) | Low | Randomization was performed according to a pre-established blocked randomization list, stratifed by centre. |
|  | Allocation concealment (selection bias) | Low | The medicine capsules were indistinguishable. |
|  | Blinding of participants and personnel (performance bias) | Low | Patients, investigators, and the coordination centre were blinded to group allocation. |
|  | Blinding of outcome assessment (detection bias) | unclear | - |
|  | Incomplete outcome data (attrition bias) | high | Some patients discontinued in this article without specific reasons. |
|  | Selective reporting (reporting bias) | Low | Outcome indicators are reported according to the experimental design. |
|  | Other bias | unclear | - |
| Behdani et al, 2013  Iran | Random sequence generation (selection bias) | Low | Patients were simply randomized to two groups in a 1:1 ratio using a computer-generated code. |
|  | Allocation concealment (selection bias) | Low | Medicine were encapsulated in a without tast and smell form. |
|  | Blinding of participants and personnel (performance bias) | unclear | - |
|  | Blinding of outcome assessment (detection bias) | Low | All investigational staff members who performed efficacy and tolerability rating scales were blind to the patient treatment group. |
|  | Incomplete outcome data (attrition bias) | Low | Patients who missed the interview all gave reasons for missing the interview. |
|  | Selective reporting (reporting bias) | Low | Outcome indicators are reported according to the experimental design. |
|  | Other bias | unclear | - |
| Bélanger et al, 2009  Canada | Random sequence generation (selection bias) | unclear | The article only mentions random allocation, but does not describe the method of random allocation. |
|  | Allocation concealment (selection bias) | unclear | - |
|  | Blinding of participants and personnel (performance bias) | unclear | - |
|  | Blinding of outcome assessment (detection bias) | unclear | - |
|  | Incomplete outcome data (attrition bias) | Low | Patients who missed the interview all gave reasons for missing the interview. |
|  | Selective reporting (reporting bias) | Low | Outcome indicators are reported according to the experimental design. |
|  | Other bias | unclear | - |
| Chang et al, 2019  China | Random sequence generation (selection bias) | Low | The randomisation numbers were generated from the computer. |
|  | Allocation concealment (selection bias) | unclear | - |
|  | Blinding of participants and personnel (performance bias) | Low | The investigators were blinded to both the group allocation during the study and when assessing the outcome measurements. |
|  | Blinding of outcome assessment (detection bias) | Low | The investigators were blinded to both the group allocation during the study and when assessing the outcome measurements. |
|  | Incomplete outcome data (attrition bias) | Low | Patients who missed the interview all gave reasons for missing the interview. |
|  | Selective reporting (reporting bias) | Low | Outcome indicators are reported according to the experimental design. |
|  | Other bias | unclear | - |
| Crippa et al, 2018  Italy | Random sequence generation (selection bias) | Low | Participants were assigned a study number and randomly allocated by an independent third person to either the supplement or the placebo group using a computer-generated randomization scheme. |
|  | Allocation concealment (selection bias) | Low | The medicine pearls matched in touch, smell, and size. |
|  | Blinding of participants and personnel (performance bias) | Low | Children, parents, and study investigators were blinded to the randomization until completion of data collection and analysis. |
|  | Blinding of outcome assessment (detection bias) | Low | Children, parents, and study investigators were blinded to the randomization until completion of data collection and analysis. |
|  | Incomplete outcome data (attrition bias) | Low | There are no missed visits in this article. |
|  | Selective reporting (reporting bias) | Low | Outcome indicators are reported according to the experimental design. |
|  | Other bias | unclear | - |
| Kean et al, 2016  Australia | Random sequence generation (selection bias) | Low | All participants were assigned to treatment groups A or B through the use of a computer-generated random number. |
|  | Allocation concealment (selection bias) | Low | The medicine capsule matched in touch, taste,smell and size. |
|  | Blinding of participants and personnel (performance bias) | Low | Blinding was achieved by enlisting a person outside of the project to code the treatments and maintain the key to this code until data collection was completed. |
|  | Blinding of outcome assessment (detection bias) | unclear | - |
|  | Incomplete outcome data (attrition bias) | high | Some patients discontinued in this article without specific reasons. |
|  | Selective reporting (reporting bias) | Low | Outcome indicators are reported according to the experimental design. |
|  | Other bias | unclear | - |
| Dubnov-Raz et al, 2014 Israel | Random sequence generation (selection bias) | unclear | The article only mentions random allocation, but does not describe the method of random allocation. |
|  | Allocation concealment (selection bias) | Low | Both types of medicine capsules were supplied in identical amounts in solid plastic bottles that numbered consecutively. |
|  | Blinding of participants and personnel (performance bias) | Low | All study participants, parents, teachers, and study personnel were blinded to the allocation until completion of all data collection. |
|  | Blinding of outcome assessment (detection bias) | Low | All study participants, parents, teachers, and study personnel were blinded to the allocation until completion of all data collection. |
|  | Incomplete outcome data (attrition bias) | Low | Patients who missed the interview all gave reasons for missing the interview. |
|  | Selective reporting (reporting bias) | Low | Outcome indicators are reported according to the experimental design. |
|  | Other bias | unclear | - |
| Gustafsson et al, 2010  Sweden | Random sequence generation (selection bias) | Low | Subjects were assigned a study number and randomized in blocks. |
|  | Allocation concealment (selection bias) | unclear | - |
|  | Blinding of participants and personnel (performance bias) | unclear | - |
|  | Blinding of outcome assessment (detection bias) | unclear | - |
|  | Incomplete outcome data (attrition bias) | Low | Patients who missed the interview all gave reasons for missing the interview. |
|  | Selective reporting (reporting bias) | Low | Outcome indicators are reported according to the experimental design. |
|  | Other bias | unclear | - |
| Hariri M et al, 2012  Iran | Random sequence generation (selection bias) | Low | Patients were randomly allocated into two groups according to prearranged balanced block randomisation. |
|  | Allocation concealment (selection bias) | Low | The medicine capsules, visually similar. |
|  | Blinding of participants and personnel (performance bias) | unclear | - |
|  | Blinding of outcome assessment (detection bias) | unclear | - |
|  | Incomplete outcome data (attrition bias) | Low | Patients who missed the interview all gave reasons for missing the interview. |
|  | Selective reporting (reporting bias) | Low | Outcome indicators are reported according to the experimental design. |
|  | Other bias | unclear | - |
| Milte et al, 2011  Australia | Random sequence generation (selection bias) | unclear | The article only mentions random allocation, but does not describe the method of random allocation. |
|  | Allocation concealment (selection bias) | unclear | - |
|  | Blinding of participants and personnel (performance bias) | Low | Parents and children were blinded to the randomization until completion of the data collection and analysis. |
|  | Blinding of outcome assessment (detection bias) | Low | Study investigators involved in the data collection were blinded to the randomization until completion of the data collection and analysis. |
|  | Incomplete outcome data (attrition bias) | Low | Patients who missed the interview all gave reasons for missing the interview. |
|  | Selective reporting (reporting bias) | Low | Outcome indicators are reported according to the experimental design. |
|  | Other bias | unclear | - |
| Moghaddam et al, 2017  Iran | Random sequence generation (selection bias) | Low | Patients were randomly divided into two groups using a random numbers table. |
|  | Allocation concealment (selection bias) | Low | The medicine were taken to the patients in pre-prepared envelopes based on code 1 and 2. |
|  | Blinding of participants and personnel (performance bias) | unclear | - |
|  | Blinding of outcome assessment (detection bias) | unclear | - |
|  | Incomplete outcome data (attrition bias) | Low | There are no missed visits in this article. |
|  | Selective reporting (reporting bias) | Low | Outcome indicators are reported according to the experimental design. |
|  | Other bias | unclear | - |
| Mohammadzadeh et al, 2019  Iran | Random sequence generation (selection bias) | Low | The randomization list was made using a computerized random-number generator based on a randomnumber Table. |
|  | Allocation concealment (selection bias) | Low | The medicine capsules, visually similar and smell identical. |
|  | Blinding of participants and personnel (performance bias) | unclear | - |
|  | Blinding of outcome assessment (detection bias) | unclear | - |
|  | Incomplete outcome data (attrition bias) | Low | Patients who missed the interview all gave reasons for missing the interview. |
|  | Selective reporting (reporting bias) | Low | Outcome indicators are reported according to the experimental design. |
|  | Other bias | unclear | - |
| Raz et al , 2009  Israel | Random sequence generation (selection bias) | unclear | The article only mentions random allocation, but does not describe the method of random allocation. |
|  | Allocation concealment (selection bias) | Low | To maintain the blinded nature of the study, the medicine were packed independently and sealed in boxes that were inserted into envelopes. |
|  | Blinding of participants and personnel (performance bias) | Low | The person who assigned subjects to groups as well as the person who packed the materials were exposed only to subject numbers (in matched pairs). |
|  | Blinding of outcome assessment (detection bias) | Low | The researcher and the family were all blinded to the condition up to this point. |
|  | Incomplete outcome data (attrition bias) | Low | Patients who missed the interview all gave reasons for missing the interview. |
|  | Selective reporting (reporting bias) | Low | Outcome indicators are reported according to the experimental design. |
|  | Other bias | unclear | - |
| Rodríguez et al, 2019  Spain | Random sequence generation (selection bias) | Low | Randomization (1:1) was performed according to a computer-generated random sequence, |
|  | Allocation concealment (selection bias) | Low | The medicine sachets had the same composition and were indistinguishable. |
|  | Blinding of participants and personnel (performance bias) | Low | Participants, parents, and investigators assessing outcome measures were blind to the intervention condition. |
|  | Blinding of outcome assessment (detection bias) | Low | Participants, parents, and investigators assessing outcome measures were blind to the intervention condition. |
|  | Incomplete outcome data (attrition bias) | Low | Patients who missed the interview all gave reasons for missing the interview. |
|  | Selective reporting (reporting bias) | Low | Outcome indicators are reported according to the experimental design. |
|  | Other bias | unclear | - |
| Salehi et al, 2015  Iran | Random sequence generation (selection bias) | Low | Selection of patients in all groups was done based on block randomization. |
|  | Allocation concealment (selection bias) | Low | The medicine capsule had the same color and the same shape. |
|  | Blinding of participants and personnel (performance bias) | unclear | - |
|  | Blinding of outcome assessment (detection bias) | unclear | - |
|  | Incomplete outcome data (attrition bias) | Low | There are no missed visits in this article. |
|  | Selective reporting (reporting bias) | Low | Outcome indicators are reported according to the experimental design. |
|  | Other bias | unclear | - |
| Widenhorn-Müller et al, 2014  Germany | Random sequence generation (selection bias) | Low | A computer-generated random sequence was used to allocate the participants. |
|  | Allocation concealment (selection bias) | unclear | - |
|  | Blinding of participants and personnel (performance bias) | Low | Participants, parents and those assessing outcome measures were blind to the intervention condition. |
|  | Blinding of outcome assessment (detection bias) | Low | Participants, parents and those assessing outcome measures were blind to the intervention condition. |
|  | Incomplete outcome data (attrition bias) | Low | Patients who missed the interview all gave reasons for missing the interview. |
|  | Selective reporting (reporting bias) | Low | Outcome indicators are reported according to the experimental design. |
|  | Other bias | unclear | - |
