# Supplementary material for: Safety and efficacy of antioxidant therapy in children and adolescents with attention deficit hyperactivity disorder: A systematic review and network meta-analysis
Source: PLoS One. 2024 Mar 28;19(3):e0296926. doi: 10.1371/journal.pone.0296926 (PMC10977718; doi:10.1371/journal.pone.0296926)
Supplement: S6 Table — (DOCX) [file pone.0296926.s007.docx]

Supplementary Material

## S7 Table. Number and severity of side effect event per study.

| **Study, Year** | **Intervention** | **Sample size** | **Number of side effect event**  **（symptom and number）** | **Severity** |
| --- | --- | --- | --- | --- |
| Rafeiy Torghabeh  et al, 2020 | Resveratrol + Methylphenidate (MPH)  Placebo + Methylphenidate (MPH) | 30  30 | Headache5/Insomnia 4 /Drowsiness 3 /Fatigue3/Dry mouth 4/Decreased appetite6/Nausea 4 /Vomiting 3 /Diarrhea 2 /Abdominal pain4  Headache7/Insomnia 6 /Drowsiness 4 /Fatigue3/Dry mouth 3/Decreased appetite8/Nausea 6 /Vomiting 4 /Diarrhea 23/Abdominal pain 3 | Only some side effects were reported as mild to moderate, but no specific details were provided |
| Motaharifard et al,  2019 | Sweet almond syrup + Placebo  Methylphenidate (MPH) + Placebo | 25  25 | Decreased appetite1/ Increased appetite15/Insomnia2/ Increased sleep4/ Difficulty falling asleep3/ Abdominal pain 2/Impulsiveness1/ Irritability1/Nausea 1  Decreased appetite15/ Increased appetite1/Insomnia6/ Increased sleep1/ Difficulty falling asleep9/ Abdominal pain 6/Headache None 2 /Impulsiveness3/ Irritability 6/Nausea 1 /Constipation 1/Dry mouth 1/Sadness 6/Tic 1/Itching1 | Not reported |
| Akhondzadeh  et al,  2005 | Passion flower  Pethylphenidate (MPH) | 17  17 | Decreased appetite1/Palpitation2/Weight loss2/ Dry mouth 1/Constipation1/Early awaking1/Headaches2/Difficulty falling asleep1  Decreased appetite7/Palpitation3/Weight loss5/ Dry mouth 3/Constipation2/Early awaking1/Headaches4/Difficulty falling asleep4/Anxiety6 | Not reported |
| Weber et al,  2008 | Hypericum perforatum  Placebo | 27  27 | Nausea,Vomiting7 /Headache4/Sunburn1  Rash4 /Nausea,Vomiting3 /Headache6/Sunburn1 | Not reported |
| Ghanizadeh et al,  2013 | Methylphenidate (MPH) + Folic  Methylphenidate (MPH) +Placebo | 23  26 | vomiting 2/decreased appetite2  vomiting1/decreased appetite1/nasal bleeding1 | Not reported |
| Salehi et al,  2009 | Ginkgo  Methylphenidate (MPH) | 25  25 | Abdominal pain 3/ nervousness 13/Decreased appetite 5/ Sadness 2/ Insomnia 3/Weight loss 3/Nausea 2 /Dry mouth 2 /Headaches 3 /Anxiety7  Abdominal pain 5/ nervousness 19/Decreased appetite 19/ Sadness 7/ Insomnia 12/Weight loss 8/Nausea 4 /Dry mouth 4 /Headaches 13 /Anxiety9 | Not reported |
| Shakibaei et al,  2015 | Ginkgo + Methylphenidate (MPH)  Placebo + Methylphenidate (MPH) | 31  29 | nausea 4/headache, diarrhea,loss of appetite 2/ constipation,abdominal pain1  loss of appetite 7/nausea,diarrhea 2/headache, palpitation, constipation, abdominal pain1 | Not reported |
| Abbasi et al,  2013 | Acetyl-L-carnitine (ALC) + Methylphenidate (MPH)  Placebo + Methylphenidate (MPH) | 20  20 | Abdominal pain 7/Anxiety 9/Decreased appetite 14/Sadness 9/Trouble in sleeping 10/Weight Loss 7/Nausea 4/Dry mouth 7/ Irritability 10/Headaches 4/Vomiting 5/Fatigue 7/Diarrhea 3/Dizziness 5  Abdominal pain 8/Anxiety 9/Decreased appetite 12/Sadness 9/Trouble in sleeping 10/Weight Loss 6/Nausea 5/Dry mouth 8/ Irritability 18/Headaches 12/Vomiting 6/Fatigue 13/Diarrhea 5/Dizziness 8 | Not reported |
| Arnold et al,  2007 | Placebo  Acetyl-L-carnitine (ALC) | 59  53 | Gastrointestinal Disorders 11/Headache 5/Infections and Infestations 16 / Nasopharyngitis 1/General disorders 3/Cough 4/Musculoskeletal and Connective Tissue Disorders 6/Metabolism and Nutrition Disorders 7/Pharyngitis 7/ Psychiatric Disorders 1/Injury, Poisoning, and Procedural Complications 8/ Nasal Congestion 2/Skin and Subcutaneous Tissue Disorders6  Gastrointestinal Disorders 16/Headache 11/Infections and Infestations 8 / Nasopharyngitis 7/General disorders 6/Cough 5/Musculoskeletal and Connective Tissue Disorders 5/Metabolism and Nutrition Disorders 4/Pharyngitis 4/ Psychiatric Disorders 4/Injury, Poisoning, and Procedural Complications 3/ Nasal Congestion 3/Skin and Subcutaneous Tissue Disorders3 | Not reported |
| Akhondzadeh  et al, 2004 | Methylphenidate (MPH) + Zinc  Methylphenidate (MPH) + placebo | 22  22 | Anxiety 3/Decreased Appetite 8/Difficulty Falling Sleep 6/Abdominal Pain 8/Nausea 9/Headache 9/Metallic taste 13  Anxiety 3/Decreased Appetite 7/Difficulty Falling Sleep 6/Abdominal Pain 4/Nausea 3/Headache 9 | Not reported |
| Arnold et al,  2011 | Zinc  Placebo | 28  24 | Affective blunting 4/Anxiety 12/Appetite 23/Central nervous system reactions 1/Dental3/Depression 15/Fatigue 10/Fever 3/Harm to self and others 1/Head, eyes, ears, nose, and throat 5/Headache 11/Hypersensitivity reaction1/Irritability 15/Musculoskeletal 1/Respiratory 5/Skin4/ Sleep 14/Stereotypical behaviors 9/Stomach aches 10/Other gastrointestinal 4/Upper repiratory infection 1  Affective blunting 6/Anxiety 5Appetite 17/Cardiovascular 2/Central nervous system reactions 7 /Depression 9/Fatigue 2/Fever 1/Head, eyes, ears, nose, and throat 2/Headache 8/Hypersensitivity reaction5/Irritability 14/Musculoskeletal 2/Respiratory 4/Skin2/ Sleep 16/Stereotypical behaviors 9/Stomach aches 11/Other gastrointestinal 3/Upper repiratory infection 2 | Not reported |
| Bilici et al,  2004 | Zinc sulfate  Placebo | 202  198 | Metallic taste 50/Nausea 8 /Vomiting 5/Abdominal pain 3/Diarrhea 1/Acute Infection 5  Metallic taste 8/Nausea 7/Vomiting 4/Abdominal pain 2/Diarrhea 1/Acute Infection 7 | Not reported |
| Hsu et al,  2021 | Pycnogenol  Placebo | 7  13 | 0  0 | Not reported |
| Trebaticka et al, 2006 | Pycnogenol  Placebo | 44  17 | gastritic discomfort2  0 | Not reported |
| Manor et al,  2012 | omega-3 + Phosphatidylserine (PS)  Placebo | 100  47 | gastroin testinal discomfort6/atopic dermatitis1/ hyperactivity1/tics1/nausea1/elevated serum glutamic oxaloacetic transaminase1/tantrum episodes2  gastrointestinal discomfort4/headache1 | Not reported |
| Hirayama et al, 2014 | Phosphatidylserine (PS)  Placebo | 19  17 | 0  0 | Not reported |
| Dehbokri et al, 2018 | Methylphenidate (MPH) + VitaminD  Methylphenidate (MPH) + Placebo | 51  45 | 0  0 | Not reported |
| Elshorbagy et al, 2018 | Methylphenidate (MPH)+ Vitamin D  Methylphenidate (MPH) + Placebo | 20  30 | mild abdominal pain1, diarrhea1, loss of appetite1  loss of appetite1 | mild  mild |
| Mohammadpour et al, 2016 | Methylphenidate (MPH) + VitaminD  Methylphenidate (MPH) + Placebo | 31  31 | Weight loss 3/Appetite loss 12/Impulsiveness 1/Sleep problems 4/Nausea 1/Feeling of fear1/Emotional instability 1  Headache 3/ Weight loss7/Appetite loss17/ Impulsiveness 1 /Stomachache 6/Sleep problems 1/Nausea 1/Vomiting1/Diarrhea 1 | Not reported |
| Vaisman et al, 2008 | omega-3 +Phosphatidylserine (PS)  omega-3  Placebo | 18  21  21 | vomiting1/rash1  vomiting2  recurrence of tics1 | Not reported |
| Rahmani et al, 2022 | Vitamin D  Placebo | 26  26 | 0  0 | Not reported |
| Hemamy et al, 2020 | Vitamin D  Placebo | 33  33 | 0  0 | Not reported |
| Assareh et al, 2012 | Methylphenidate (MPH) + omega-3+6  Methylphenidate (MPH) + Placebo | 20  20 | 0  0 | Not reported |
| Barragán et al, 2014 | Methylphenidate (MPH)  omega-3+6  Methylphenidate (MPH) + omega-3+6. | 30  30  30 | Hyporexia 2/Dyspepsia12/Diarrhea 7  Hyporexia21/Headache17/Irritability7/Tension8/Pallor7/Palpitation7/Insomnia6/Tics2/Tremor1/Nausea 1  Hyporexia 10/Headache10/Tension3/Pallor11/Palpitation 5 | Not reported |
| Carucci et al, 2021 | omega-3+6  Placebo | 67  68 | diarrhoea2  abdominal pain1/itch1/ somnolence1 | Not reported |
| Johnson et al, 2012 | omega-3+6  Placebo | 37  38 | dyspepsia1/vomiting1  diarrhea1/ irritability1 | Not reported |
| Cornu et al,  2017 | omega-3  Placebo | 71  77 | hip pain2/fatigue2/headache2/fever ,cough2/ dermatitis, allergic reaction2/abdominal pain, diarrhoea3  fatigue2/infuenza2/abdominal pain1/dermatitis1/ swollen eyes1/vomiting1/ diarrhoea1 | Not reported |
| Behdani et al, 2013 | Methylphenidate (MPH) + omega-3  Methylphenidate (MPH) + Placebo | 36  33 | 0  0 | Not reported |
| Bélanger et al,  2009 | omega-3  omega-6 | 13  13 | 0  0 | Not reported |
| Chang et al,  2019 | omega-3  placebo | 48  44 | 0  0 | Not reported |
| Crippa et al,  2018 | omega-3  Placebo | 25  25 | 0  0 | Not reported |
| Gustafsson et al, | omega-3  Placebo | 46  46 | nausea5/diarrhoea3/nose bleeding1  nausea6/diarrhoea4/nose bleeding3 | Not reported |
| Matsudaira et al, 2015 | omega-3+6  Placebo | 38  38 | 5 (no specific details were provided)  7 (no specific details were provided) | Only reported 10 mild, 1 moderate, 1 severe, but no specific details were provided |
| Milte et al,  2011 | omega-3  omega-6 | 58  29 | flatulence1/yellow teeth1/unpleasant taste2/ nose bleeds1  bad breath1 | 5 mild  1 mild |
| Moghaddam  et al, 2017 | Methylphenidate (MPH) + omega-3  Methylphenidate (MPH) + Placebo | 20  20 | sleep disorders5/anorexia5/headache5/dizziness4/depression/burping1  sleep disorders5/abdominal pain5/dizziness5/restlessness5 | Not reported |
| Mohammadzadeh et al, 2019 | Methylphenidate (MPH) + omega-3  Methylphenidate (MPH) + Placebo | 33  33 | Nausea 2/ Vomiting 4/Diarrhea 8/Stomachache 1 /Dry mouth 1/Drowsiness 2 /Insomnia 2 /Anxiety 1/Anorexia 41 /Restlessness 3/ Irritability 3  Nausea 1 Vomiting 1/Diarrhea 10/Stomachache 1 /Dry mouth 1/Drowsiness 1 /Insomnia 2 /Anxiety 1/Anorexia 27 /Restlessness 2 / Irritability 2/Seizure 1 | Not reported |
| Raz et al ,  2009 | omega-3  Placebo | 32  31 | Abdomen rash1/toe aches1/Behavioral deterioration in class1/ restlessness1/Headache 1  Headache1/Argumentative 1/Behavioral deterioration1/nervous1/Dandruff 1/Increased appetite1/Oppositional1/definant 1/Restlessness 1/Stomach aches 1/Vocal tic1/difficulty waking up1 | Not reported |
| Rodríguez et al, 2019 | omega-3  Placebo | 32  34 | 0  0 | Not reported |
| Salehi et al,  2015 | Methylphenidate (MPH)+omega-3  Methylphenidate (MPH)+Zinc sulfate  Methylphenidate (MPH)+Placebo | 50  50  50 | 0  0  0 | Not reported |
| Widenhorn-Müller et al,  2014 | omega-3  Placebo | 46  49 | 0  0 | Not reported |
