# Supplementary material for: Safety and efficacy of antioxidant therapy in children and adolescents with attention deficit hyperactivity disorder: A systematic review and network meta-analysis
Source: PLoS One. 2024 Mar 28;19(3):e0296926. doi: 10.1371/journal.pone.0296926 (PMC10977718; doi:10.1371/journal.pone.0296926)
Supplement: S7 Table — (DOCX) [file pone.0296926.s008.docx]

Supplementary Material

## S8 Table. Symptom and Number of side effect event per study.

| **Study, Year** | **Intervention** | **Headache** | **Anxiety**  **/nervou-sness** | **Insomnia** | **Drowsiness** | **Sadness** | **Dry mouth** | **Decreased appetite** | **Increased appetite** | **Nausea** | **Vomit** | **Diarrhea** | **Abdomin-al pain** | **Weight loss** | **Palpita-tion** | **Others** |
| --- | --- | --- | --- | --- | --- | --- | --- | --- | --- | --- | --- | --- | --- | --- | --- | --- |
| Rafeiy Torghabeh  et al, 2020 | Resveratrol + MPH  Placebo + MPH | 5  7 | 0  0 | 4  6 | 3  4 | 0  0 | 4  3 | 6  8 | 0  0 | 4  6 | 3  4 | 2  23 | 4  3 | 0  0 | 0  0 | 3  3 |
| Motaharifard et al,  2019 | Sweet almond syrup  + Placebo  MPH + Placebo | 0  2 | 0  0 | 5  15 | 4  1 | 0  6 | 0  1 | 1  15 | 15  1 | 1  1 | 0  0 | 0  0 | 2  6 | 0  0 | 0  0 | 2  12 |
| Akhondzadeh  et al,  2005 | Passion flower  MPH | 2  4 | 0  6/0 | 2  5 | 0  0 | 0  0 | 1  3 | 1  7 | 0  0 | 0  0 | 0  0 | 0  0 | 0  0 | 2  5 | 2  3 | 1  2 |
| Weber et al,  2008 | Hypericum perforatum  Placebo | 4  6 | 0  0 | 0  0 | 0  0 | 0  0 | 0  0 | 0  0 | 0  0 | 7  3 | 7  3 | 0  0 | 0  0 | 0  0 | 0  0 | 1  5 |
| Ghanizadeh et al,  2013 | MPH + Folic  MPH +Placebo | 0  0 | 0  0 | 0  0 | 0  0 | 0  0 | 0  0 | 2  1 | 0  0 | 0  0 | 2  1 | 0  0 | 0  0 | 0  0 | 0  0 | 0  1 |
| Salehi et al,  2009 | Ginkgo  MPH | 3  13 | 7/13  9/19 | 3  12 | 0  0 | 2  7 | 2  4 | 5  5 | 0  0 | 2  4 | 2  0 | 0  0 | 3  5 | 3  8 | 0  0 | 0  0 |
| Shakibaei  et al,  2015 | Ginkgo + MPH  Placebo + MPH | 2  1 | 0  0 | 0  0 | 0  0 | 0  0 | 0  0 | 2  7 | 0  0 | 4  2 | 0  0 | 2  2 | 1  1 | 0  0 | 0  1 | 1  1 |
| Abbasi et al,  2013 | Acetyl-L-carnitine  + MPH  Placebo + MPH | 4  12 | 9/0  9/0 | 10  0 | 0  0 | 9  9 | 7  8 | 14  12 | 0  0 | 4  5 | 5  6 | 3  5 | 7  8 | 7  6 | 0  0 | 22  49 |
| Arnold et al,  2007 | Placebo  Acetyl-L-carnitine | 5  11 | 0  0 | 0  0 | 0  0 | 0  0 | 0  0 | 0  0 | 0  0 | 0  0 | 0  0 | 0  0 | 11  16 | 0  0 | 0  0 | 51  53 |
| Akhondzadeh  et al,  2004 | MPH + Zinc  MPH + placebo | 9  9 | 3/0  3/0 | 6  6 | 0  0 | 0  0 | 0  0 | 8  7 | 0  0 | 9  3 | 0  0 | 0  0 | 8  4 | 0  0 | 0  0 | 13  0 |
| Arnold et al,  2011 | Zinc  Placebo | 11  8 | 27/0  14/0 | 7  8 | 7  8 | 0  0 | 0  0 | 12  9 | 11  8 | 0  0 | 0  0 | 0  3 | 0  11 | 0  0 | 0  0 | 62  58 |
| Bilici et al,  2004 | Zinc sulfate  Placebo | 0  0 | 0  0 | 0  0 | 0  0 | 0  0 | 0  0 | 0  0 | 0  0 | 8  7 | 5  4 | 1  1 | 3  2 | 0  0 | 0  0 | 55  15 |
| Hsu et al,  2021 | Pycnogenol  Placebo | 0  0 | 0  0 | 0  0 | 0  0 | 0  0 | 0  0 | 0  0 | 0  0 | 0  0 | 0  0 | 0  0 | 0  0 | 0  0 | 0  0 | 0  0 |
| Trebaticka  et al, 2006 | Pycnogenol  Placebo | 0  0 | 0  0 | 0  0 | 0  0 | 0  0 | 0  0 | 0  0 | 0  0 | 0  0 | 0  0 | 0  0 | 0  0 | 0  0 | 0  0 | 0  0 |
| Manor et al,  2012 | omega-3 + Phosphatidylserine  Placebo | 0  1 | 2  0 | 0  0 | 0  0 | 0  0 | 0  0 | 0  0 | 0  0 | 1  0 | 0  0 | 0  0 | 6  4 | 0  0 | 0  0 | 4  0 |
| Hirayama  et al, 2014 | Phosphatidylserine  Placebo | 0  0 | 0  0 | 0  0 | 0  0 | 0  0 | 0  0 | 0  0 | 0  0 | 0  0 | 0  0 | 0  0 | 0  0 | 0  0 | 0  0 | 0  0 |
| Dehbokri  et al, 2018 | MPH + VitaminD  MPH + Placebo | 0  0 | 0  0 | 0  0 | 0  0 | 0  0 | 0  0 | 0  0 | 0  0 | 0  0 | 0  0 | 0  0 | 0  0 | 0  0 | 0  0 | 0  0 |
| Elshorbagy  et al, 2018 | MPH+ Vitamin D  MPH + Placebo | 0  0 | 0  0 | 0  0 | 0  0 | 0  0 | 0  0 | 1  1 | 0  0 | 0  0 | 0  0 | 1  0 | 1  0 | 0  0 | 0  0 | 0  0 |
| Mohammadp-our et al, 2016 | MPH + VitaminD  MPH + Placebo | 0  3 | 0  0 | 2  1 | 2  0 | 0  0 | 0  0 | 12  17 | 0  0 | 1  1 | 0  1 | 0  1 | 0  6 | 3  3 | 0  0 | 1  1 |
| Vaisman  et al, 2008 | omega-3 +Phosphatidylserine  omega-3  Placebo | 0  0  0 | 0  0  0 | 0  0  0 | 0  0  0 | 0  0  0 | 0  0  0 | 0  0  0 | 0  0  0 | 0  0  0 | 1  2  0 | 0  0  0 | 0  0  0 | 0  0  0 | 0  0  0 | 1  0  1 |
| Rahmani  et al, 2022 | Vitamin D  Placebo | 0  0 | 0  0 | 0  0 | 0  0 | 0  0 | 0  0 | 0  0 | 0  0 | 0  0 | 0  0 | 0  0 | 0  0 | 0  0 | 0  0 | 0  0 |
| Hemamy  et al, 2020 | Vitamin D  Placebo | 0  0 | 0  0 | 0  0 | 0  0 | 0  0 | 0  0 | 0  0 | 0  0 | 0  0 | 0  0 | 0  0 | 0  0 | 0  0 | 0  0 | 0  0 |
| Assareh  et al, 2012 | MPH + omega-3+6  MPH + Placebo | 0  0 | 0  0 | 0  0 | 0  0 | 0  0 | 0  0 | 0  0 | 0  0 | 0  0 | 0  0 | 0  0 | 0  0 | 0  0 | 0  0 | 0  0 |
| Barragán  et al, 2014 | MPH  omega-3+6  MPH + omega-3+6 | 0  17  10 | 0  0  0/3 | 0  6  0 | 0  0  0 | 0  0  0 | 0  0  0 | 0  0  0 | 0  0  0 | 0  1  0 | 0  0  0 | 7  0  0 | 12  0  0 | 0  0  0 | 0  0  5 | 2  53  21 |
| Carucci et al, 2021 | omega-3+6  Placebo | 0  0 | 0  0 | 0  0 | 0  1 | 0  0 | 0  0 | 0  0 | 0  0 | 0  0 | 0  0 | 2  0 | 0  1 | 0  0 | 0  0 | 0  1 |
| Johnson et al, 2012 | omega-3+6  Placebo | 0  0 | 0  1 | 0  0 | 0  0 | 0  0 | 0  0 | 0  0 | 0  0 | 0  0 | 1  0 | 0  1 | 1  0 | 0  0 | 0  0 | 0  0 |
| Cornu et al,  2017 | omega-3  Placebo | 2  0 | 0  0 | 0  0 | 0  0 | 0  0 | 0  0 | 0  0 | 0  0 | 0  0 | 0  1 | 2  1 | 1  1 | 0  0 | 0  0 | 8  6 |
| Behdani et al, 2013 | MPH + omega-3  MPH + Placebo | 0  0 | 0  0 | 0  0 | 0  0 | 0  0 | 0  0 | 0  0 | 0  0 | 0  0 | 0  0 | 0  0 | 0  0 | 0  0 | 0  0 | 0  0 |
| Bélanger  et al,  2009 | omega-3  omega-6 | 0  0 | 0  0 | 0  0 | 0  0 | 0  0 | 0  0 | 0  0 | 0  0 | 0  0 | 0  0 | 0  0 | 0  0 | 0  0 | 0  0 | 0  0 |
| Chang et al,  2019 | omega-3  placebo | 0  0 | 0  0 | 0  0 | 0  0 | 0  0 | 0  0 | 0  0 | 0  0 | 0  0 | 0  0 | 0  0 | 0  0 | 0  0 | 0  0 | 0  0 |
| Crippa et al,  2018 | omega-3  Placebo | 0  0 | 0  0 | 0  0 | 0  0 | 0  0 | 0  0 | 0  0 | 0  0 | 0  0 | 0  0 | 0  0 | 0  0 | 0  0 | 0  0 | 0  0 |
| Gustafsson et al, | omega-3  Placebo | 0  0 | 0  0 | 0  0 | 0  0 | 0  0 | 0  0 | 0  0 | 0  0 | 5  6 | 0  0 | 3  4 | 0  0 | 0  0 | 0  0 | 1  3 |
| Matsudaira  et al, 2015 | omega-3+6  Placebo | 0  0 | 0  0 | 0  0 | 0  0 | 0  0 | 0  0 | 0  0 | 0  0 | 0  0 | 0  0 | 0  0 | 0  0 | 0  0 | 0  0 | 5  7 |
| Milte et al,  2011 | omega-3  omega-6 | 0  0 | 0  0 | 0  0 | 0  0 | 0  0 | 0  0 | 0  0 | 0  0 | 0  0 | 0  0 | 0  0 | 0  0 | 0  0 | 0  0 | 5  1 |
| Moghaddam  et al, 2017 | MPH + omega-3  MPH + Placebo | 9  5 | 1/0  5/0 | 5  5 | 0  0 | 0  0 | 0  0 | 5  0 | 0  0 | 0  0 | 0  0 | 0  0 | 0  5 | 0  0 | 0  0 | 1  0 |
| Mohammadz-adeh et al, 2019 | MPH + omega-3  MPH + Placebo | 0  0 | 4/3  3/2 | 2  2 | 2  1 | 0  0 | 1  1 | 41  27 | 0  0 | 2  1 | 4  1 | 8  10 | 1  1 | 0  0 | 0  0 | 0  1 |
| Raz et al ,  2009 | omega-3  Placebo | 1  1 | 1/0  1/0 | 0  0 | 0  1 | 0  0 | 0  0 | 0  0 | 0  1 | 0  0 | 0  0 | 0  0 | 0  1 | 0  0 | 0  0 | 3  7 |
| Rodríguez  et al, 2019 | omega-3  Placebo | 0  0 | 0  0 | 0  0 | 0  0 | 0  0 | 0  0 | 0  0 | 0  0 | 0  0 | 0  0 | 0  0 | 0  0 | 0  0 | 0  0 | 0  0 |
| Salehi et al,  2015 | MPH +omega-3  MPH +Zinc sulfate  MPH +Placebo | 0  0  0 | 0  0  0 | 0  0  0 | 0  0  0 | 0  0  0 | 0  0  0 | 0  0  0 | 0  0  0 | 0  0  0 | 0  0  0 | 0  0  0 | 0  0  0 | 0  0  0 | 0  0  0 | 0  0  0 |
| Widenhorn-Müller et al,  2014 | omega-3  Placebo | 0  0 | 0  0 | 0  0 | 0  0 | 0  0 | 0  0 | 0  0 | 0  0 | 0  0 | 0  0 | 0  0 | 0  0 | 0  0 | 0  0 | 0  0 |
| Total | - | 167 | 144 | 112 | 34 | 33 | 35 | 226 | 36 | 88 | 53 | 82 | 136 | 37 | 11 | - |
