# Supplementary material for: Safety and efficacy of antioxidant therapy in children and adolescents with attention deficit hyperactivity disorder: A systematic review and network meta-analysis
Source: PLoS One. 2024 Mar 28;19(3):e0296926. doi: 10.1371/journal.pone.0296926 (PMC10977718; doi:10.1371/journal.pone.0296926)
Supplement: S8 Table — (DOCX) [file pone.0296926.s009.docx]

Supplementary Material

## S9 Table. Definition and Interpretation of Outcome Indicators and Statistical Analysis

1. **Definition and Interpretation of Outcome Indicators**

| **Outcomes** | **Indicators** | **Definitions** | **Variables types** | **Data used in statistical analysis** | **Results in statistical analysis** |
| --- | --- | --- | --- | --- | --- |
| Safety  (Primary) | The number of adverse events | Adverse events refer to all harmful reactions or symptoms reported after intervention in each study from the beginning to the end of the study. The number of adverse events can be recorded by parents, patients themselves or doctors those occurring only during the implementation stage of intervention measures, excluding the long-term adverse impact of these intervention measures after the end of the study. | Dichotomous variables | Ratio of number of adverse events to number of patients in this group  (If the total number of adverse events is greater than the number of patients, the maximum number of patients is selected as the number of adverse events) | OR (95%CrI)  An OR value greater than 1 indicates that adverse reactions are more likely to occur; An OR value of less than 1 indicates that adverse reactions are less likely to occur.  At the same time, if 95%CrI contains 1, the results are not statistically different. If 95%CrI does not contain 1, the results are statistically different. |
| Efficacy  (Primary) | Conners' parent rating scale  (CPRS) | CPRS is completed by parents that assesses the attention, hyperactivity and general situation of children with ADHD.  The higher the score, the more severe the symptoms, and the lower the score, the less severe the symptoms. | Continuous variables | Difference between scores after intervention and before intervention  (If the difference is negative, the score is lower than before intervention; if the difference is positive, the score is higher than before intervention) | MD (95%CrI)  If 95%CrI contains 0, the results are not statistically different.  If 95%CrI does not contain 0, the results are statistically different. |
| Efficacy  (Primary) | Conners' teacher rating scale  (CTRS) | CTRS is completed by teachers that assesses the attention, hyperactivity and general situation of children with ADHD.  The higher the score, the more severe the symptoms, and the lower the score, the less severe the symptoms. | Continuous variables | Difference between scores after intervention and before intervention  (If the difference is negative, the score is lower than before intervention; if the difference is positive, the score is higher than before intervention) | MD (95%CrI)  If 95%CrI contains 0, the results are not statistically different.  If 95%CrI does not contain 0, the results are statistically different. |
| Efficacy  (Primary) | ADHD rating scale-parent  (ADHD RS-Parent) | ADHD RS-Parent is completed by parents that assesses the attention, hyperactivity and general situation of children with ADHD.  The higher the score, the more severe the symptoms, and the lower the score, the less severe the symptoms. | Continuous variables | Difference between scores after intervention and before intervention  (If the difference is negative, the score is lower than before intervention; if the difference is positive, the score is higher than before intervention) | MD (95%CrI)  If 95%CrI contains 0, the results are not statistically different.  If 95%CrI does not contain 0, the results are statistically different. |
| Efficacy  (Primary) | ADHD rating scale-Teacher  (ADHD RS-Teacher) | ADHD RS-Teacher is completed by teachers that assesses the attention, hyperactivity and general situation of children with ADHD.  The higher the score, the more severe the symptoms, and the lower the score, the less severe the symptoms. | Continuous variables | Difference between scores after intervention and before intervention  (If the difference is negative, the score is lower than before intervention; if the difference is positive, the score is higher than before intervention) | MD (95%CrI)  If 95%CrI contains 0, the results are not statistically different.  If 95%CrI does not contain 0, the results are statistically different. |
| Efficacy  (secondary) | Clinical Global Impressions scale (CGI) | CGI assesses the therapeutic effect of ADHD children. The lower the CGI score, the better the therapeutic effect. In each study, we choose the number of people with CGI≤2 as the evaluation index.  The more people, the better the therapeutic effect, and the less people, the worse the therapeutic effect. | Dichotomous variables | The ratio of the number of people with CGI score ≤2 to the total number of people in this group after treatment | OR (95%CrI)  If 95%CrI contains 1, the results are not statistically different.  If 95%CrI does not contain 1, the results are statistically different. |
| Efficacy  (secondary) | Continuous Performance Test  (CPT) | CPT measures the hit reaction time of children by computer.  The longer the time, the worse the attention, and the shorter the time, the better the attention. | Continuous variables | Difference between lengths of time after intervention and before intervention  (If the difference is negative, the time required to concentrate is lower than before; if the difference is positive, the time required to concentrate is higher than before.) | MD (95%CrI)  If 95%CrI contains 0, the results are not statistically different.  If 95%CrI does not contain 0, the results are statistically different. |

## Definition and Interpretation of Statistical Analysis

| **Types** | **Definitions** | **Results** |
| --- | --- | --- |
| Evidence network | We used R studio 4.2.1 software to plot the network graphics included in the study to present direct and indirect comparisons between different intervention measures. | A network graphic, each node represents an intervention measure. |
| Consistency test | If closed-loop structure exists in the network graphic drawn, we use consistency tests to evaluate consistency between direct evidence and indirect evidence. | We use OR(95%CrI) and P value to show the results of consistency test. If P≥ 0.05, there is no statistical difference between direct comparison and indirect comparison, and the consistency is good. If P<0.05, there is statistical difference between direct comparison and indirect comparison, and the consistency is poor. |
| Heterogeneity test | In the drawn network graph, each node represents an intervention measure, and the thickness of the lines between nodes is positively correlated with the number of studies. If the number of studies is ≧ 2, we use heterogeneity tests to evaluate the heterogeneity among the studies. | We use I^2^ to show the results of heterogeneity test.  I^2^=50% was considered as the critical value of the use of effects models. If I^2^ ≤ 50%, it indicated that the heterogeneity was small, and the fixed-effects model was selected; otherwise, the random-effects model was selected. |
| Network analysis | There are comparisons between intervention measures in the network graph drawn, and we present the results of the network meta-analysis in league tables. | The league table is an inverted triangle table symmetrically distributed along the diagonal. The figures in the table are the difference values between the vertical column and the horizontal column, which are expressed by the effect value (OR or MD) and the confidence interval (95%CrI), so as to judge whether the intervention measures are statistically significant. In this study, we have used red and blue areas to divide them.  For dichotomous variables, the results are presented in OR (95%CrI). If 95%CrI contains 1, the results are not statistically different. If 95%CrI does not contain 1, the results are statistically different.  For continuous variables, the results are presented in MD (95%CrI). If 95%CrI contains 0, the results are not statistically different. If 95%CrI does not contain 0, the results are statistically different. |
| Probability ranking | We used R studio 4.2.1 software to calculate SUCRA, the surface under the cumulative ranking curve, which is used to rank the safety and efficacy of each intervention measure. | In this study, the SUCRA value shows the percentage of safety or efficacy of each intervention measure, and the range of SUCRA value is 0-1. The closer the value is to 0, the lower the probability is, while the closer the value is to 1, the higher the probability is.  In this paper, in the assessment of safety, we use dichotomous variables (the number of adverse events) to calculate SUCRA values. The closer the value to 0, the lower the probability of adverse events, and the higher the ranking, while the closer the value to 1, the higher the probability of adverse events, and the lower the ranking.  In the assessment of efficacy, we use dichotomous variables (the number of people with CGI score ≤2) to calculate SUCRA values. The closer the value is to 0, the lower the probability of children with CGI score ≤2, and the lower the ranking, while the closer the value is to 1, the higher the probability of children with CGI score ≤2, and the higher the ranking.  In the assessment of efficacy, we use continuous variables (CPRS, CTRS, ADHD RS-Parent, ADHD RS-Teacher, difference between the scores after CPT intervention and before CPT intervention or the difference of the length of time) to calculate the SUCRA value. The closer the value is to 0, the lower the probability of increasing the score (time) and the higher the ranking, while the closer the value is to 1, the higher the probability of increasing the score (time) and the lower the ranking. |
