# Supplementary material for: Safety and efficacy of antioxidant therapy in children and adolescents with attention deficit hyperactivity disorder: A systematic review and network meta-analysis
Source: PLoS One. 2024 Mar 28;19(3):e0296926. doi: 10.1371/journal.pone.0296926 (PMC10977718; doi:10.1371/journal.pone.0296926)
Supplement: S1 Fig — (DOCX) [file pone.0296926.s010.docx]

Supplementary Material

## S1 Fig. Consistency Test.

1. **Consistency test for safety of antioxidant therapy**


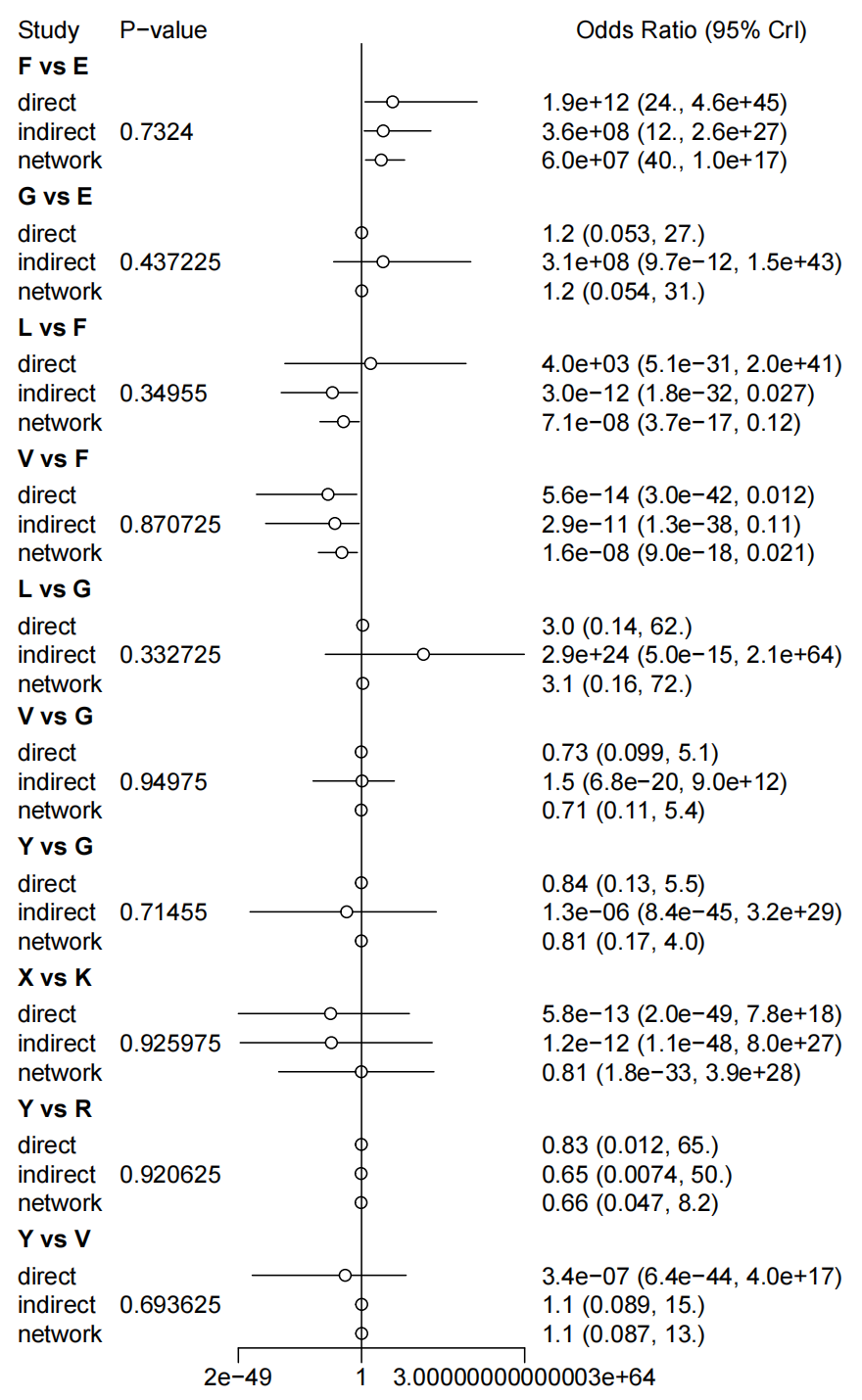


Note: Results are showed as OR (95% CrI). F=MPH, E=Quercetin, G=Placebo, V=omega-3+6, L=Zinc, Y=omega-3, K=Zinc+MPH, X=omega-3+MPH, R=Phosphatidylserine+omega-3, V=omega-3+6. omega-3=omega-3 fatty acids, omega-3+6=omega-3 fatty acids plus omega-6 fatty acids, omega-6=omega-6 fatty acids, MPH=Methylphenidate, OR=Odds ratio, CrI=credibility interval.

1. **Consistency test for attention score of Conners’ Parent Rating Scale**


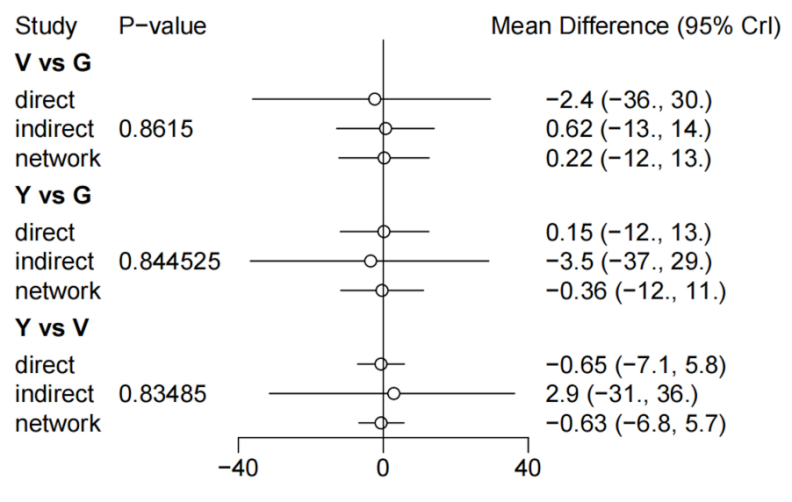


Note: Results are showed as MD (95% CrI). V=omega-3+6, G=Placebo, Y=omega-3. omega-3=omega-3 fatty acids, omega-6=omega-6 fatty acids, omega-3+6=omega-3 fatty acids plus omega-6 fatty acids, MPH=Methylphenidate, MD=mean difference, CrI=credibility interval.

1. **Consistency test for total score of Conners’ Parent Rating Scale (network A)**


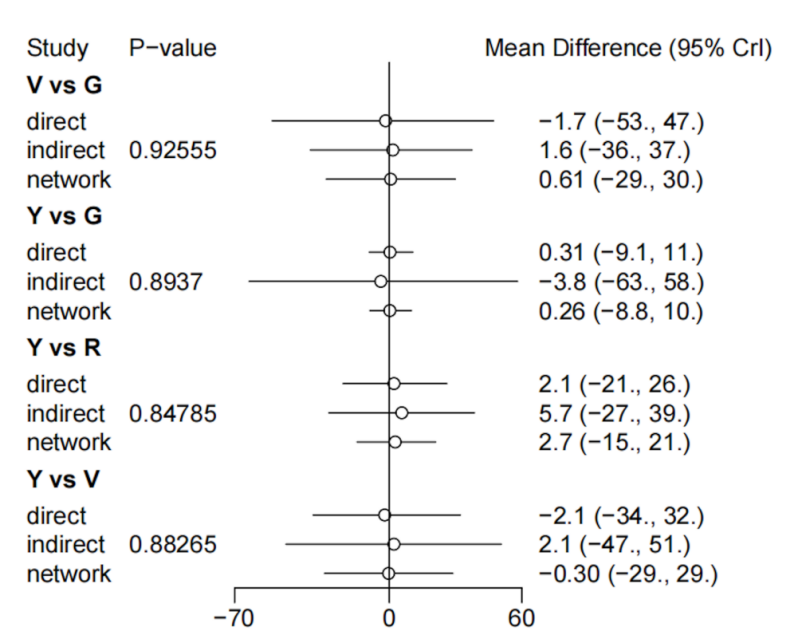


Note: Results are showed as MD (95% CrI). V=omega-3+6, R=Phosphatidylserine+omega-3, G=Placebo, Y=omega-3, V=omega-3+6. omega-3+6=omega-3 fatty acids plus omega-6 fatty acids, omega-3=omega-3 fatty acids, MPH=Methylphenidate, omega-6=omega-6 fatty acids, MD=mean difference, CrI=credibility interval.
