# Supplementary material for: Safety and efficacy of antioxidant therapy in children and adolescents with attention deficit hyperactivity disorder: A systematic review and network meta-analysis
Source: PLoS One. 2024 Mar 28;19(3):e0296926. doi: 10.1371/journal.pone.0296926 (PMC10977718; doi:10.1371/journal.pone.0296926)
Supplement: S2 Fig — (DOCX) [file pone.0296926.s011.docx]

Supplementary Material

## S2 Fig. Heterogeneity Test.

1. **Heterogeneity test for safety of antioxidant therapy**


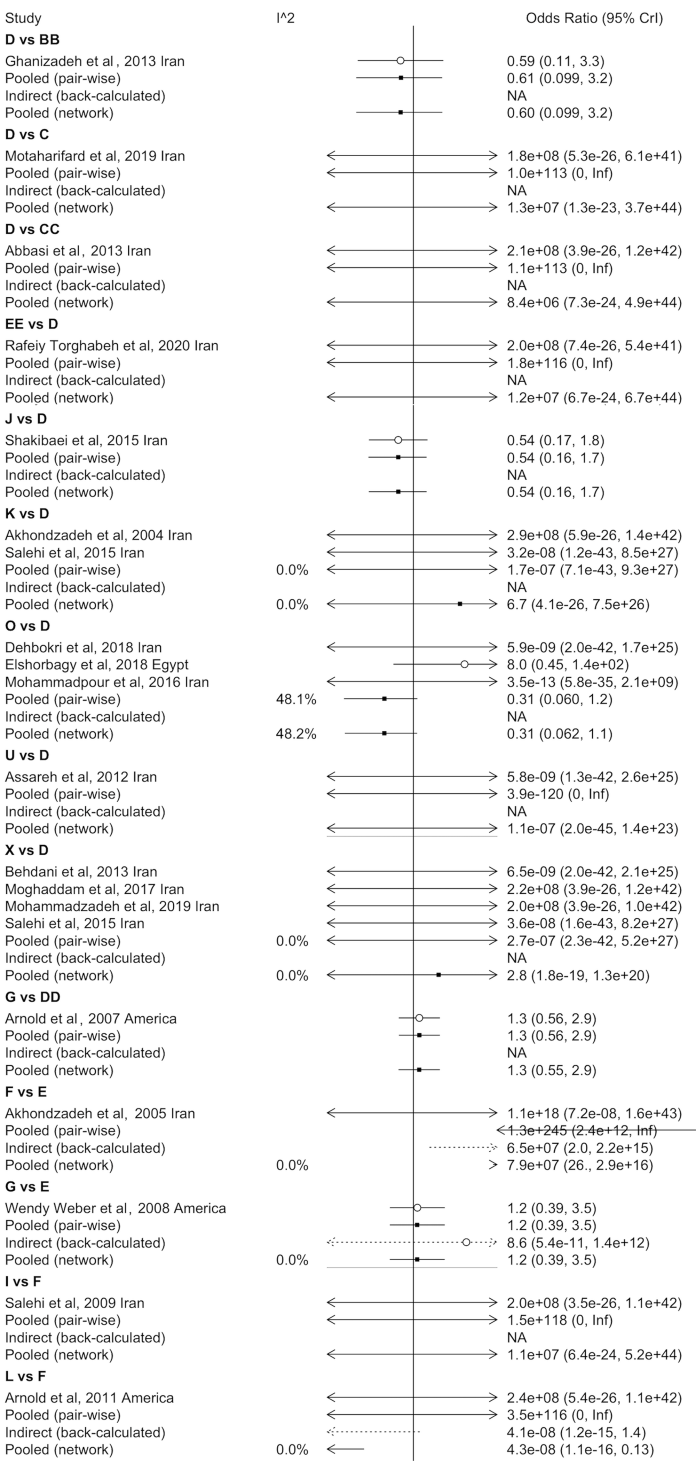

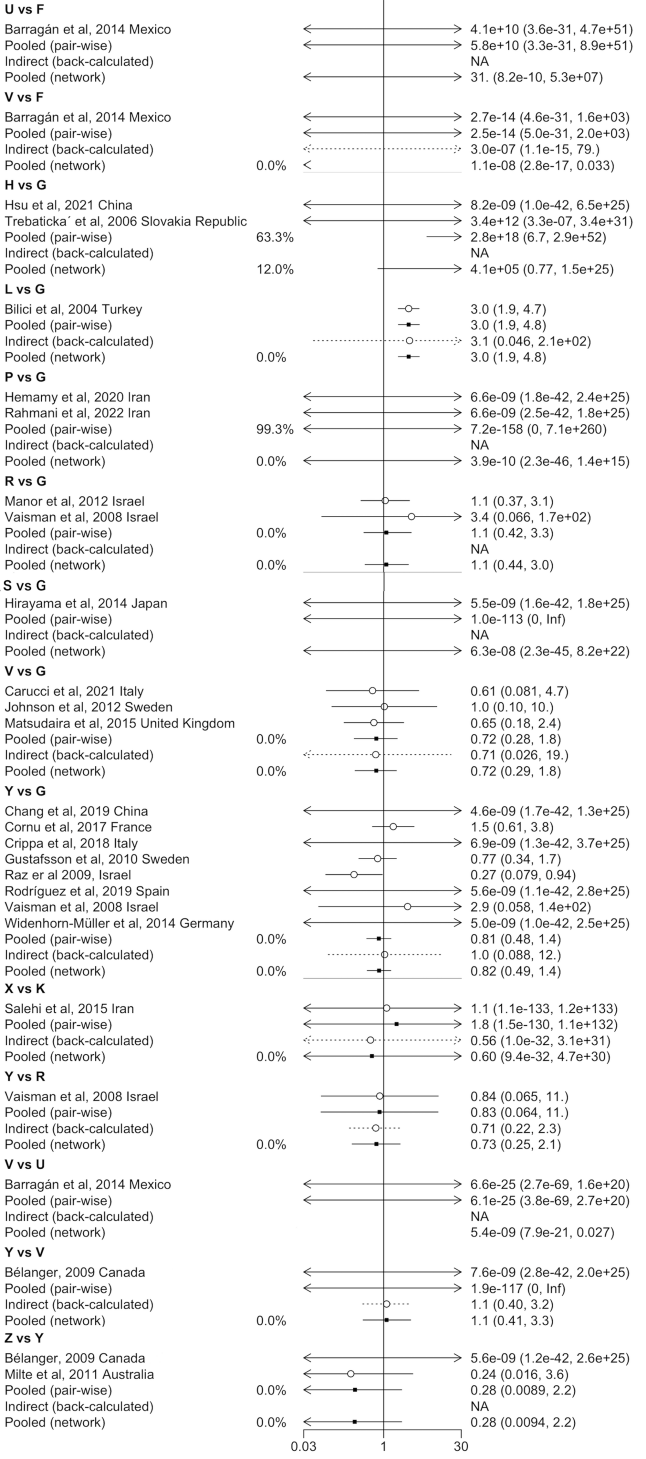


Note: Results are showed as OR (95% CrI). C=Sweet almond syrup+Placebo, D=Placebo+MPH, E=Quercetin, F=MPH, G=Placebo, H=Pycnogenol, I=Ginkgo, J=Ginkgo+MPH, K=Zinc+MPH, L=Zinc, P=Vitamin D, R=Phosphatidylserine+omega-3, S=Phosphatidylserine, U=omega-3+6 + MPH, V=omega-3+6, X=omega-3+MPH, Y=omega-3, Z=omega-6, BB=Folic+MPH, CC=Acetyl-L-carnitine+MPH, DD=Acetyl-L-carnitine, EE=Resveratrol+MPH. omega-3=omega-3 fatty acids, omega-6=omega-6 fatty acids, omega-3+6=omega-3 fatty acids plus omega-6 fatty acids, MPH=Methylphenidate, OR=Odds ratio, CrI=credibility interval.

1. **Heterogeneity test for attention score of Conners’ Parent Rating Scale**


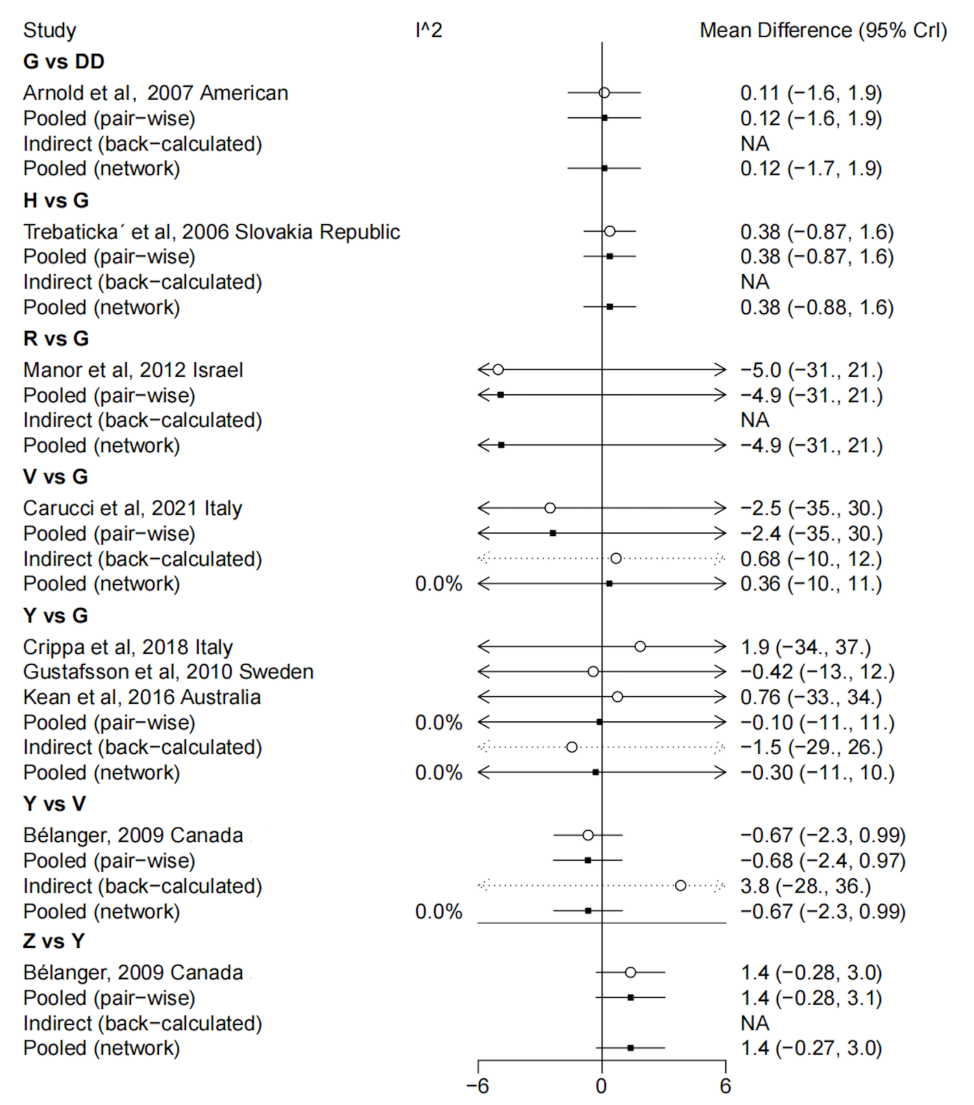


Note: Results are showed as MD (95% CrI). G=Placebo, H=Pycnogenol, V=omega-3+6, R=Phosphatidylserine+omega-3, Y=omega-3, Z=omega-6, DD=Acetyl-L-carnitine. omega-3=omega-3 fatty acids, omega-6=omega-6 fatty acids, omega-3+6=omega-3 fatty acids plus omega-6 fatty acids, MPH=Methylphenidate, MD=mean difference, CrI=credibility interval.

1. **Heterogeneity test for hyperactivity score of Conners’ Parent Rating Scale**


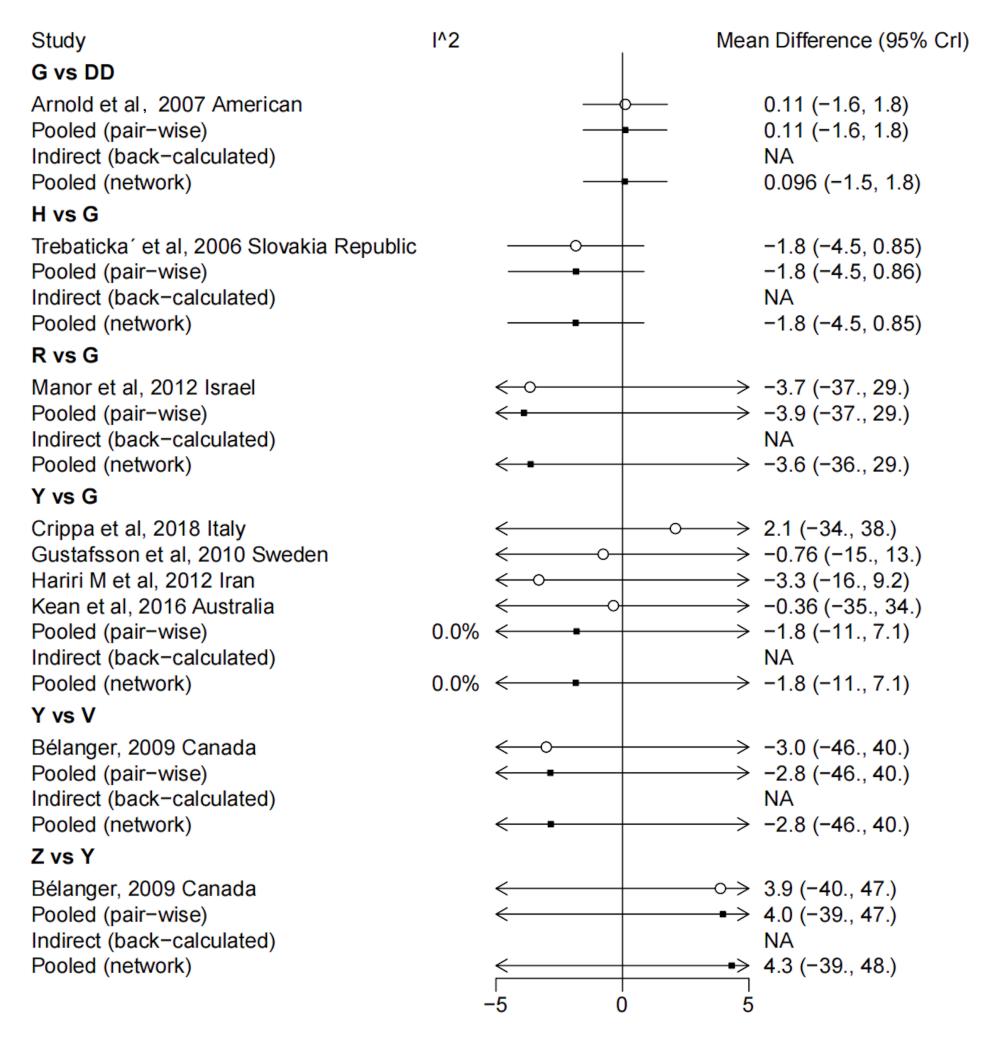


Note: Results are showed as MD (95% CrI). G=Placebo, H=Pycnogenol, V=omega-3+6, R=Phosphatidylserine+omega-3, Y=omega-3, Z=omega-6, DD=Acetyl-L-carnitine. omega-3=omega-3 fatty acids, omega-6=omega-6 fatty acids, omega-3+6=omega-3 fatty acids plus omega-6 fatty acids, MPH=Methylphenidate, MD=mean difference, CrI=credibility interval.

1. **Heterogeneity test for total score of Conners’ Parent Rating Scale(network A)**

**
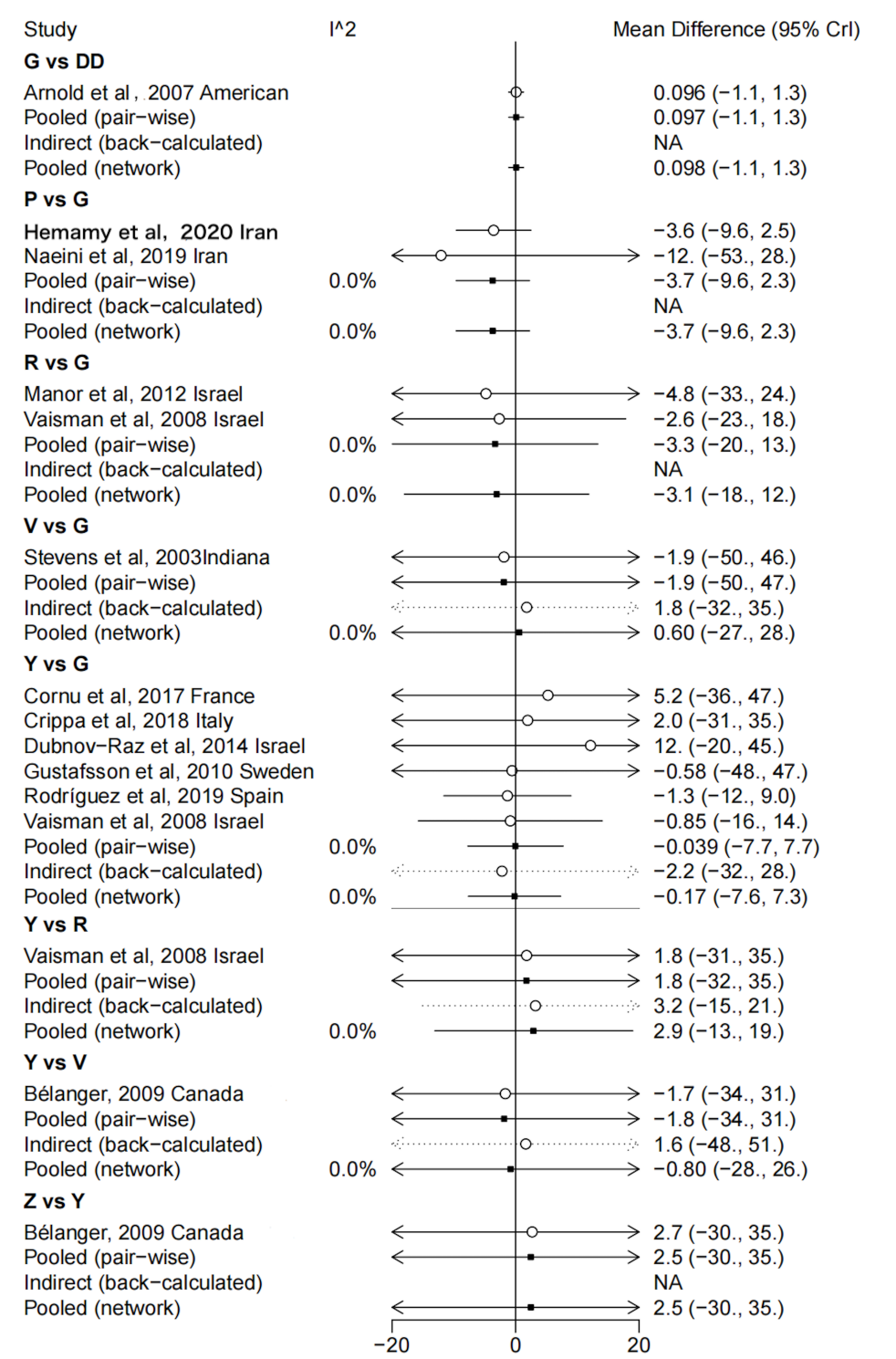
**

Note: Results are showed as MD (95% CrI). G=Placebo, P=Vitamin D, R=Phosphatidylserine+omega-3, V=omega-3+6, Y=omega-3, Z=omega-6, DD=Acetyl-L-carnitine. omega-3=omega-3 fatty acids, omega-6=omega-6 fatty acids, omega-3+6=omega-3 fatty acids plus omega-6 fatty acids, MPH=Methylphenidate, MD=mean difference, CrI=credibility interval.

1. **Heterogeneity test for total score of Conners’ Parent Rating Scale(network B)**

**
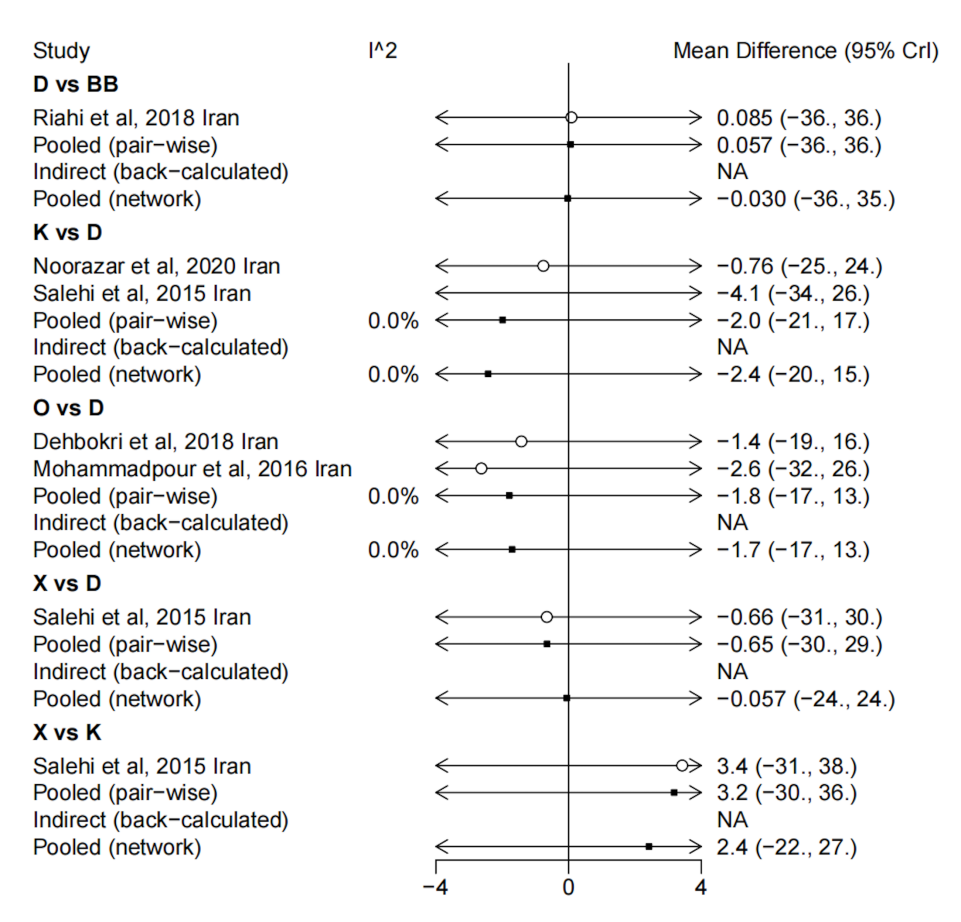
**

Note: Results are showed as MD (95% CrI). D=Placebo+MPH, K=Zinc+MPH, O=Vitamin D+MPH, X=omega-3+MPH, BB=Folic+MPH. omega-3=omega-3 fatty acids, omega-6=omega-6 fatty acids, omega-3+6=omega-3 fatty acids plus omega-6 fatty acids, MPH=Methylphenidate, MD=mean difference, CrI=credibility interval.

1. **Heterogeneity test for attentionl score of Conners’ Teacher Rating Scale**


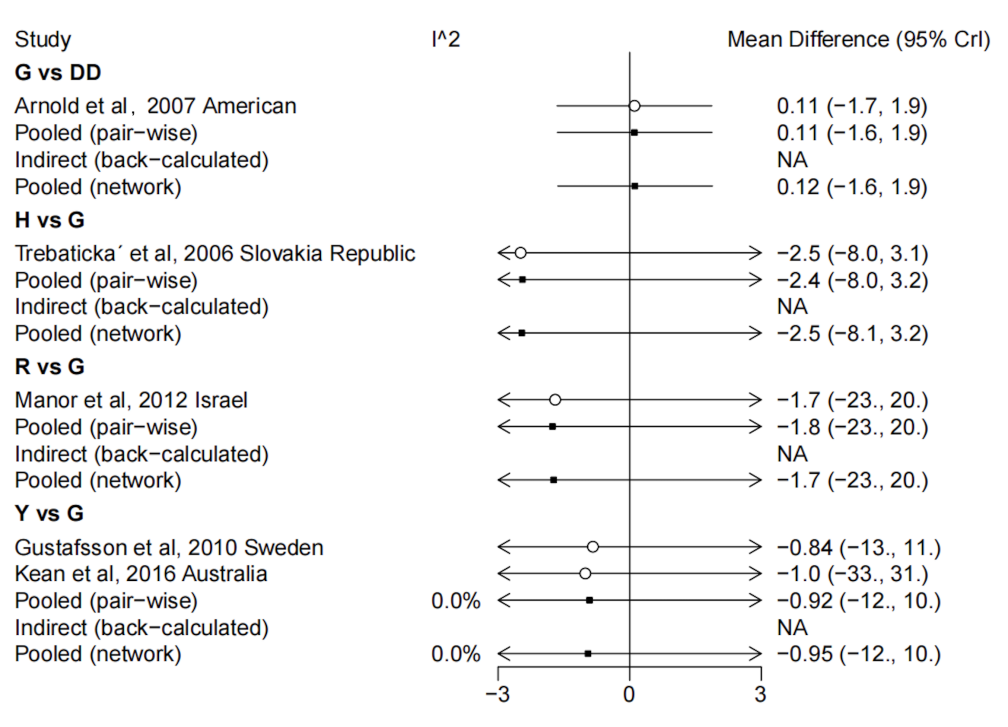


Note: Results are showed as MD (95% CrI). G=Placebo, H=Pycnogenol, R=Phosphatidylserine+omega-3, V=omega-3+6, DD=Acetyl-L-carnitine. omega-3=omega-3 fatty acids, omega-6=omega-6 fatty acids, omega-3+6=omega-3 fatty acids plus omega-6 fatty acids, MPH=Methylphenidate, MD=mean difference, CrI=credibility interval.

1. **Heterogeneity test for total score of Conners’ Teacher Rating Scale**


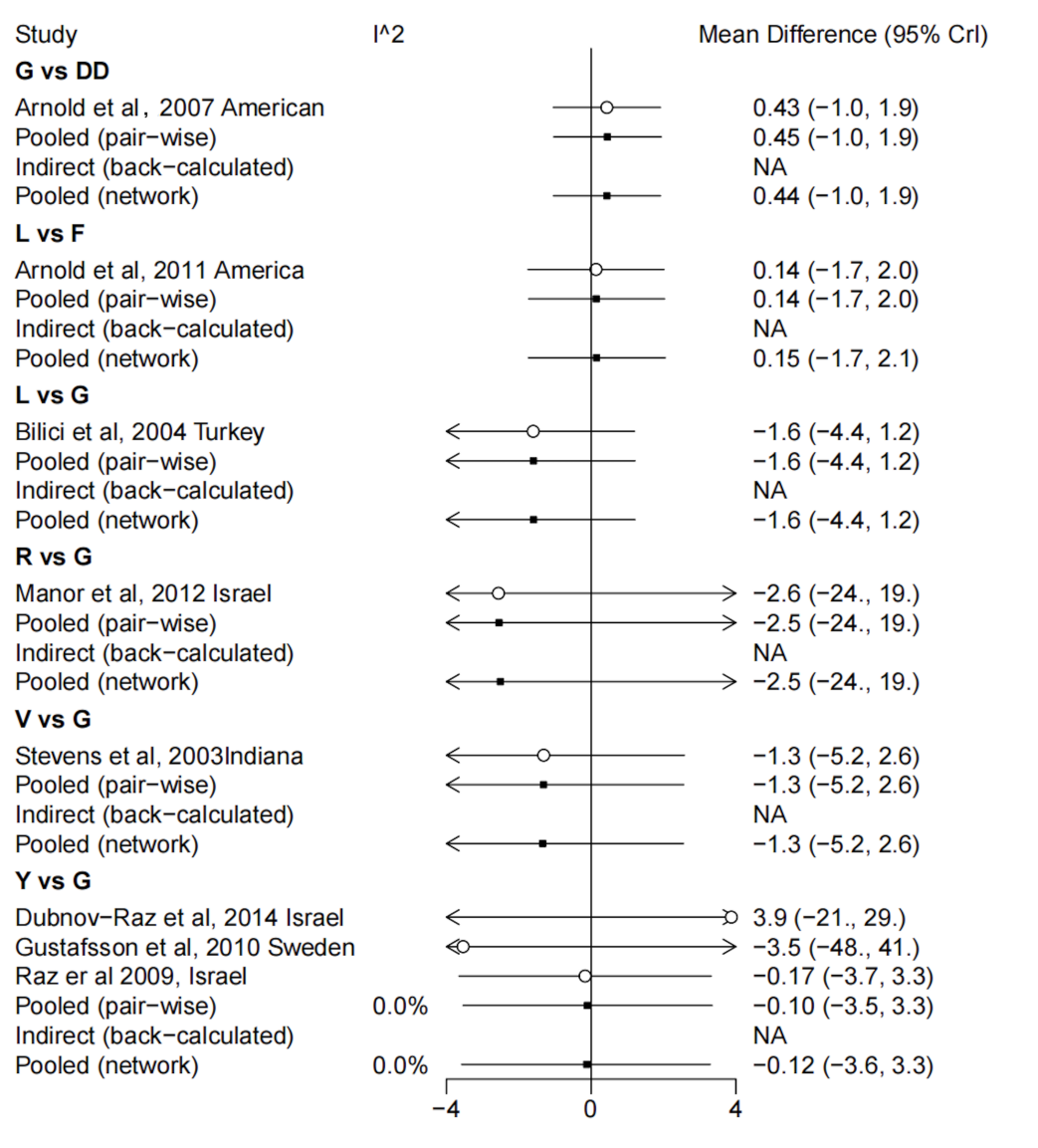


Note: Results are showed as MD (95% CrI). F=MPH, G=Placebo, L=Zinc, R=Phosphatidylserine+omega-3, V=omega-3+6, Y=omega-3, DD=Acetyl-L-carnitine. omega-3=omega-3 fatty acids, omega-6=omega-6 fatty acids, omega-3+6=omega-3 fatty acids plus omega-6 fatty acids, MPH=Methylphenidate, MD=mean difference, CrI=credibility interval.

1. **Heterogeneity test for attention score of ADHD Rating Scale-Parent**


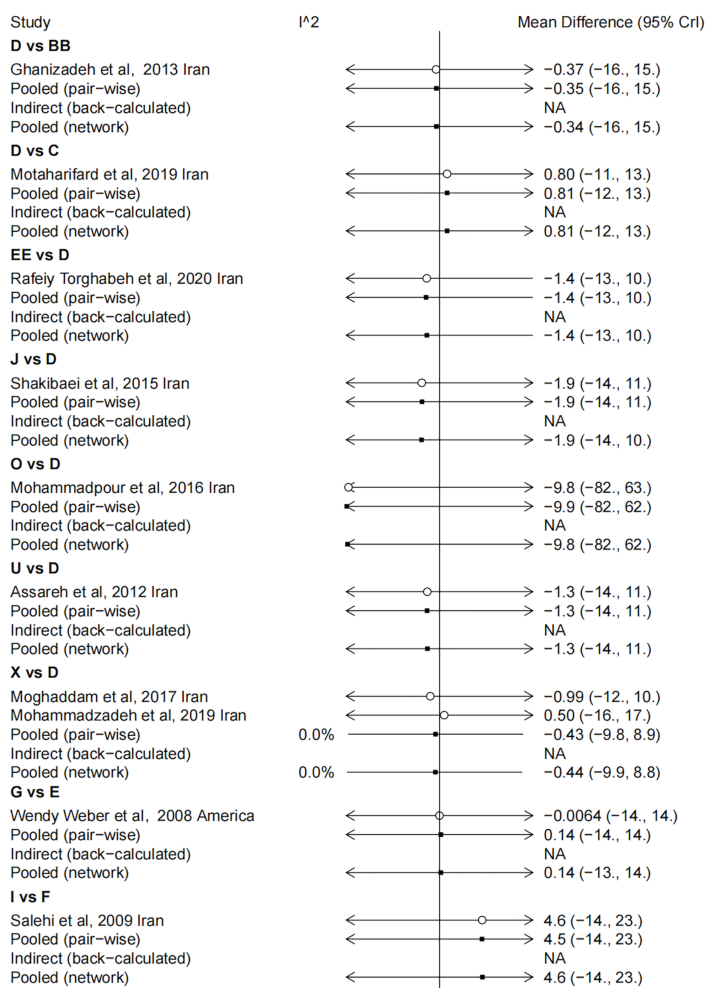

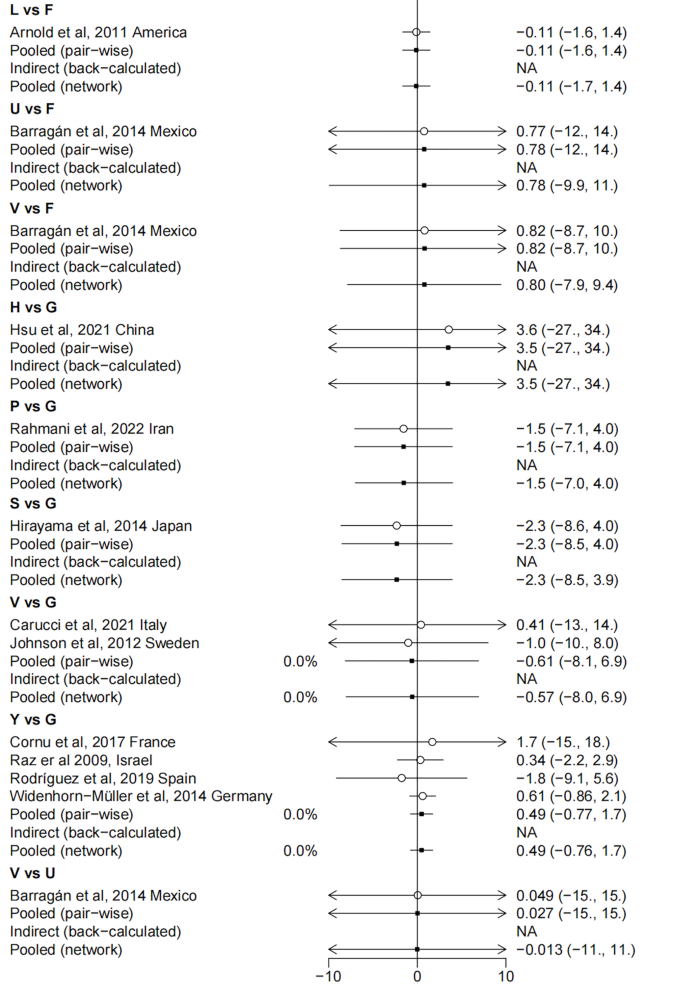


Note: Results are showed as MD (95% CrI). C=Sweet almond syrup+Placebo, D=Placebo+MPH, E=Quercetin, F=MPH, G=Placebo, I=Ginkgo, J=Ginkgo+MPH, L=Zinc, O=Vitamin D+MPH, P=Vitamin D, S=Phosphatidylserine, U=omega-3+6 + MPH, V=omega-3+6, X=omega-3+MPH, Y=omega-3, BB=Folic+MPH, EE=Resveratrol+MPH. omega-3=omega-3 fatty acids, omega-6=omega-6 fatty acids, omega-3+6=omega-3 fatty acids plus omega-6 fatty acids, MPH=Methylphenidate, MD=mean difference, CrI=credibility interval.

1. **Heterogeneity test for hyperactivity score of ADHD Rating Scale-Parent**


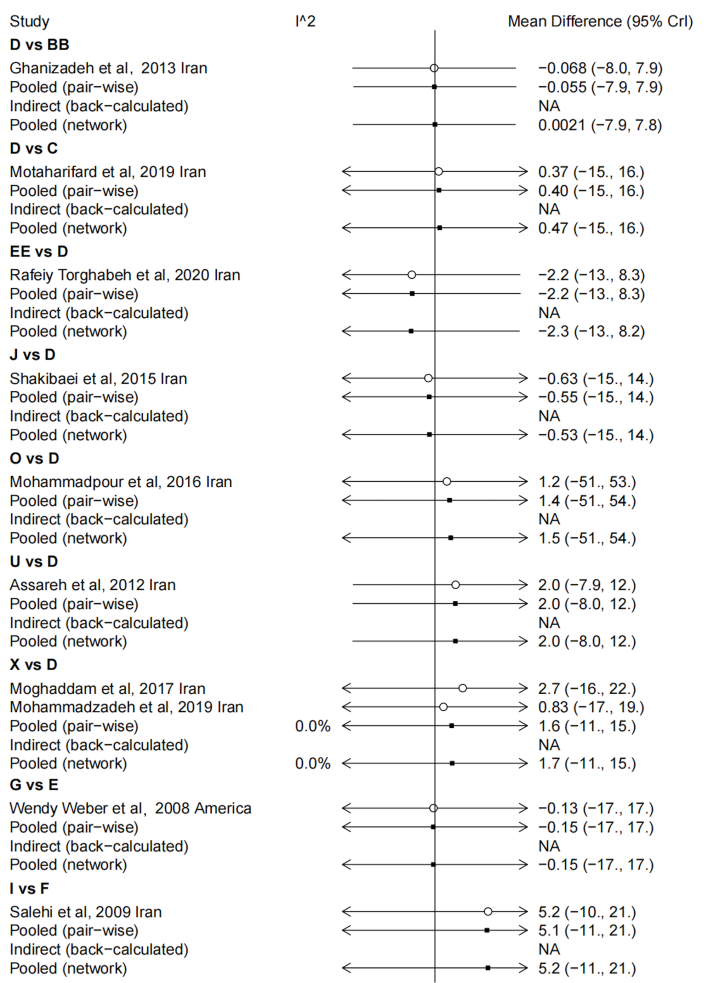

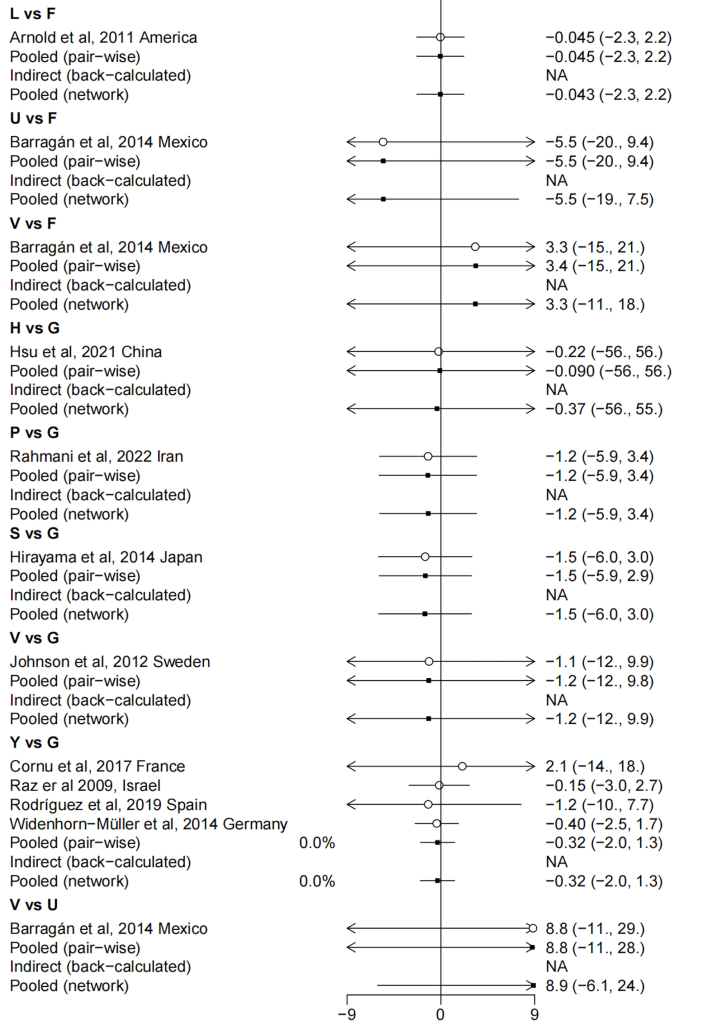


Note: Results are showed as MD (95% CrI). C=Sweet almond syrup+Placebo, D=Placebo+MPH, E=Quercetin, F=MPH, G=Placebo, H=Pycnogenol, I=Ginkgo, J=Ginkgo+MPH, L=Zinc, O=Vitamin D+MPH, P=Vitamin D, S=Phosphatidylserine, U=omega-3+6 + MPH, V=omega-3+6, X=omega-3+MPH, Y=omega-3, BB=Folic+MPH, EE=Resveratrol+MPH. omega-3=omega-3 fatty acids, omega-6=omega-6 fatty acids, omega-3+6=omega-3 fatty acids plus omega-6 fatty acids, MPH=Methylphenidate, MD=mean difference, CrI=credibility interval.

1. **Heterogeneity test for total score of ADHD Rating Scale-Parent (network A)**


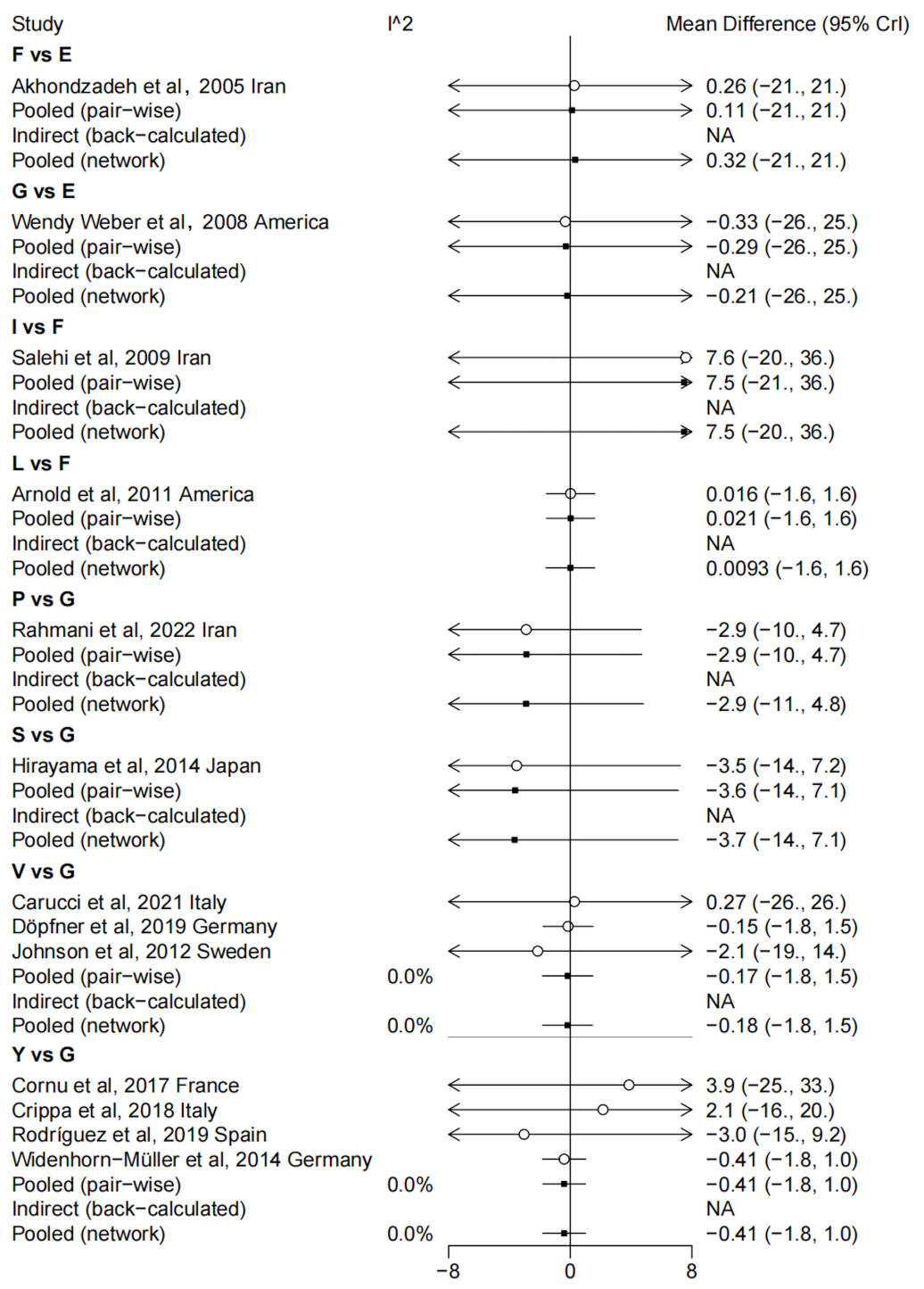


Note: Results are showed as MD (95% CrI). E=Quercetin, F=MPH, G=Placebo, I=Ginkgo, L=Zinc, P=Vitamin D, S=Phosphatidylserine, V=omega-3+6, Y=omega-3. omega-3=omega-3 fatty acids, omega-6=omega-6 fatty acids, omega-3+6=omega-3 fatty acids plus omega-6 fatty acids, MPH=Methylphenidate, MD=mean difference, CrI=credibility interval.

1. **Heterogeneity test for total score of ADHD Rating Scale-Parent (network B)**


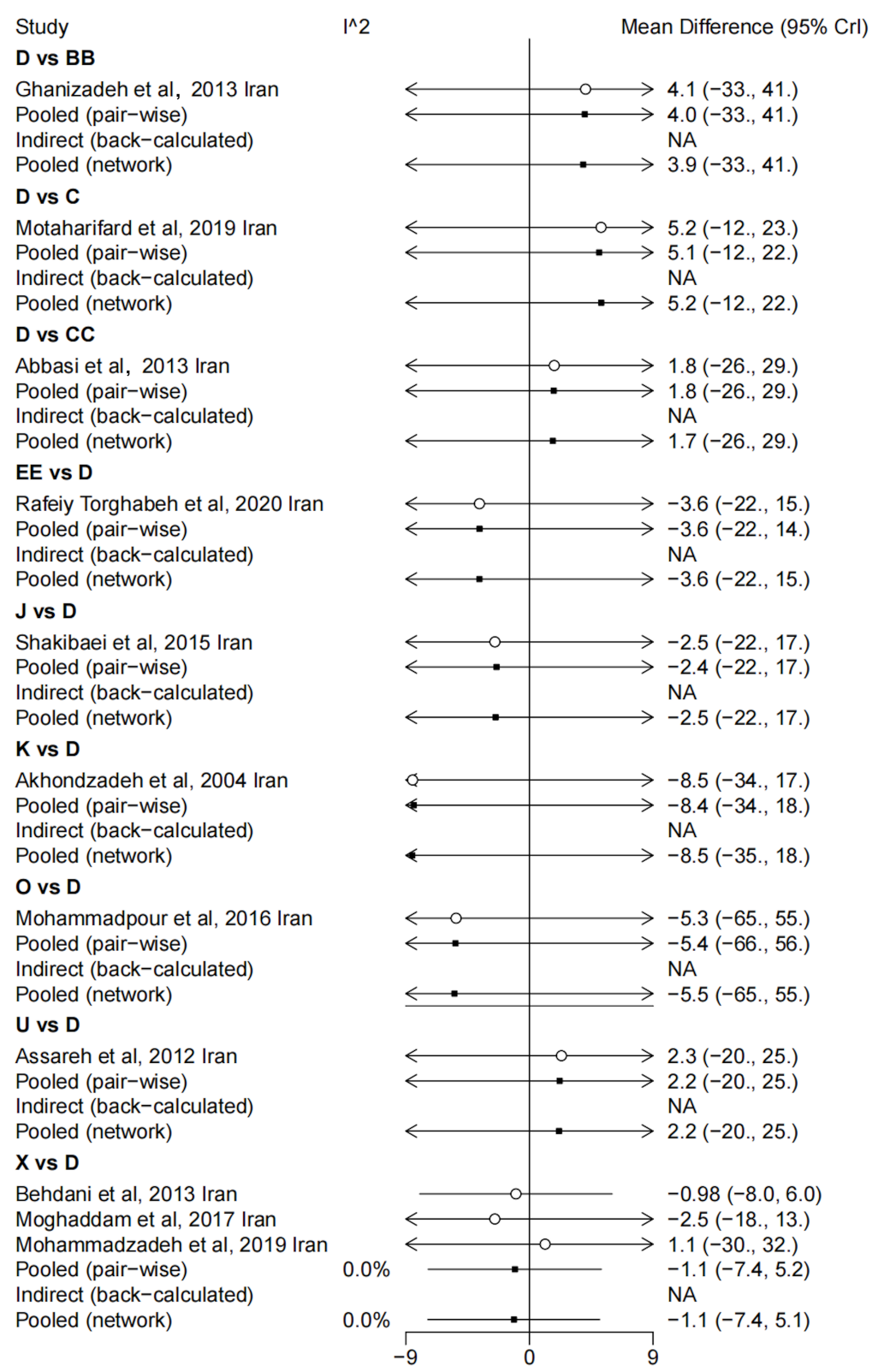


Note: Results are showed as MD (95% CrI). C=Sweet almond syrup+Placebo, D=Placebo+MPH, J=Gingko+MPH, K=Zinc+MPH, O=Vitamin D+MPH, U=omega-3+6 + MPH, X=omega-3+MPH, BB=Folic+MPH, CC=Acetyl-L-carnitine+MPH, EE=Resveratrol+MPH. omega-3=omega-3 fatty acids, omega-6=omega-6 fatty acids, omega-3+6=omega-3 fatty acids plus omega-6 fatty acids, MPH=Methylphenidate, MD=mean difference, CrI=credibility interval.
