# Supplementary material for: Safety and efficacy of antioxidant therapy in children and adolescents with attention deficit hyperactivity disorder: A systematic review and network meta-analysis
Source: PLoS One. 2024 Mar 28;19(3):e0296926. doi: 10.1371/journal.pone.0296926 (PMC10977718; doi:10.1371/journal.pone.0296926)
Supplement: S3 Fig — (DOCX) [file pone.0296926.s012.docx]

Supplementary Material

## S3 Fig. Network geometry.

1. **Evidence network for safety of antioxidant therapy**

**(23 intervention nodes, 12 antioxidants, 41 studies, and 3141 participants)**

**
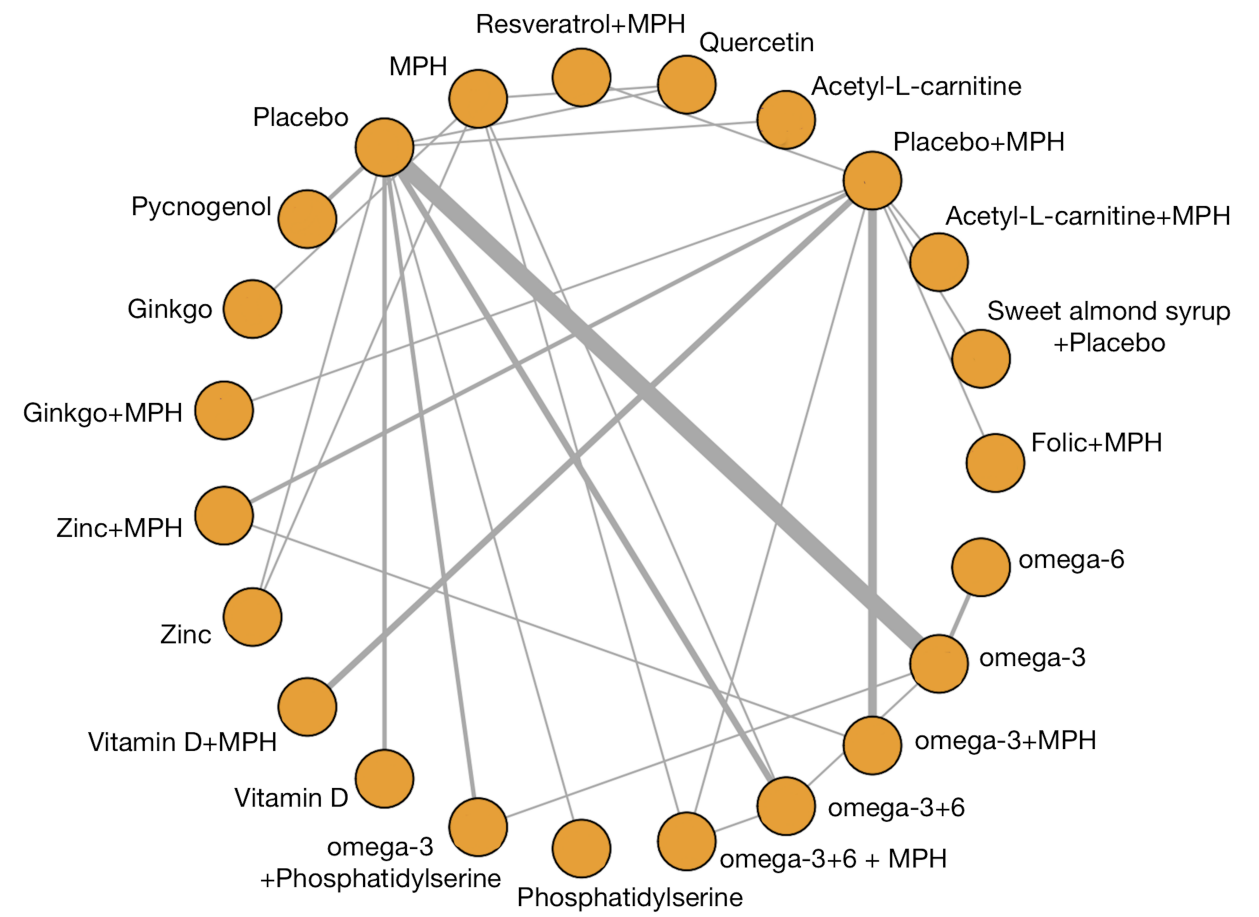
**

1. **Evidence network for attention score of Conners’ Parent Rating Scale**

**(7 intervention nodes, 5 antioxidants, 9 studies, and 761 participants)**

**
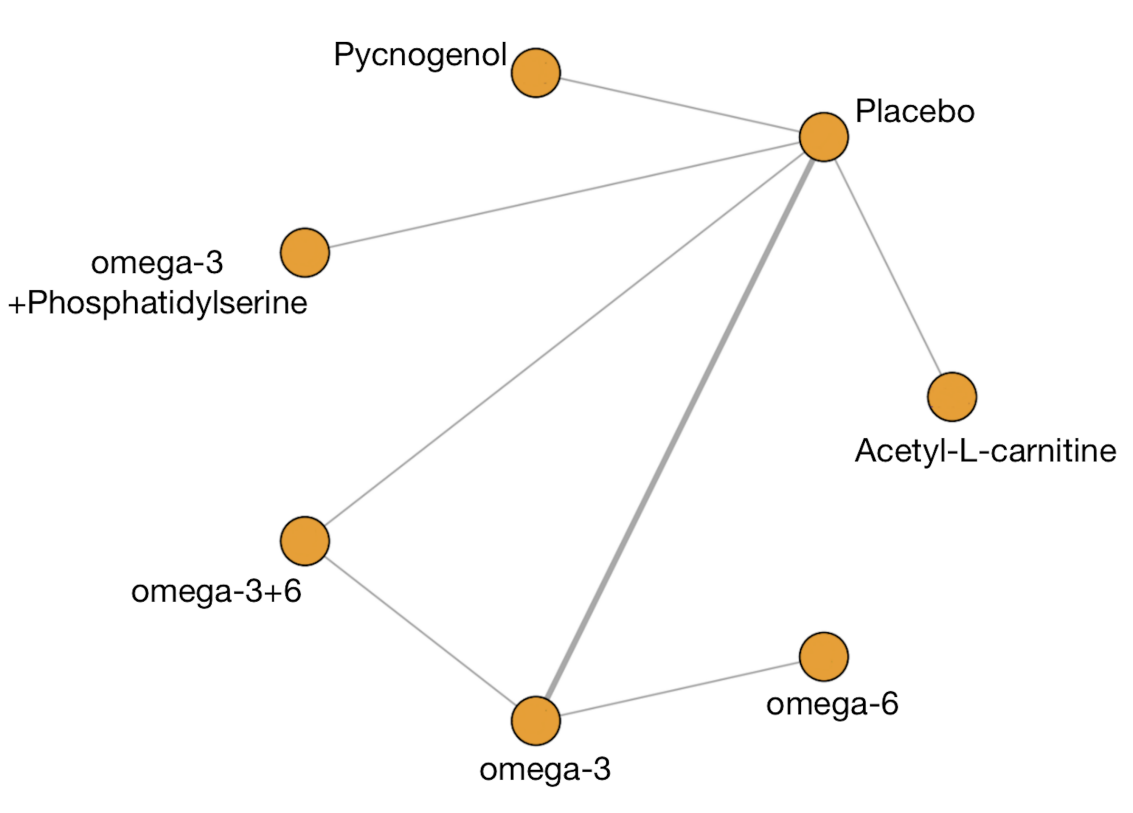
**

1. **Evidence network for hyperactivity score of Conners’ Parent Rating Scale**

**(7 intervention nodes, 5 antioxidants, 9 studies, and 729 participants)**

**
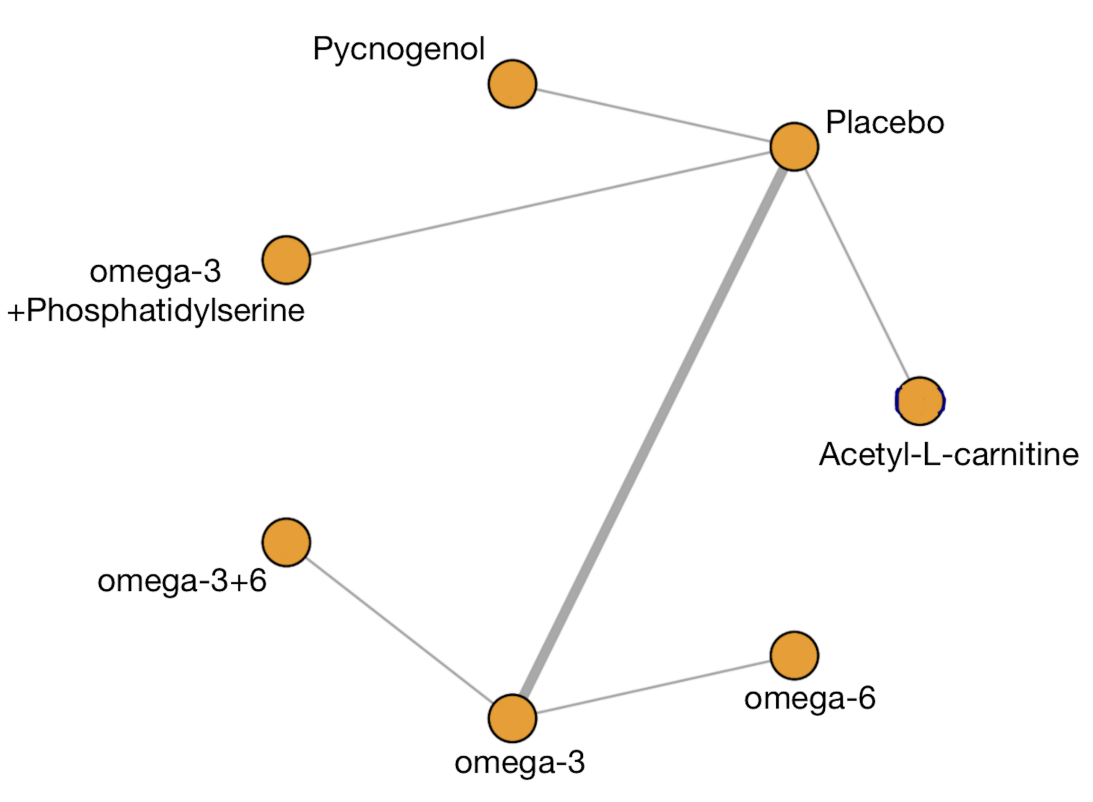
**

1. **Evidence network for total score of Conners’ Parent Rating Scale (network A)**

**(7 intervention nodes, 5 antioxidants, 12 studies, and 928 participants)**

**
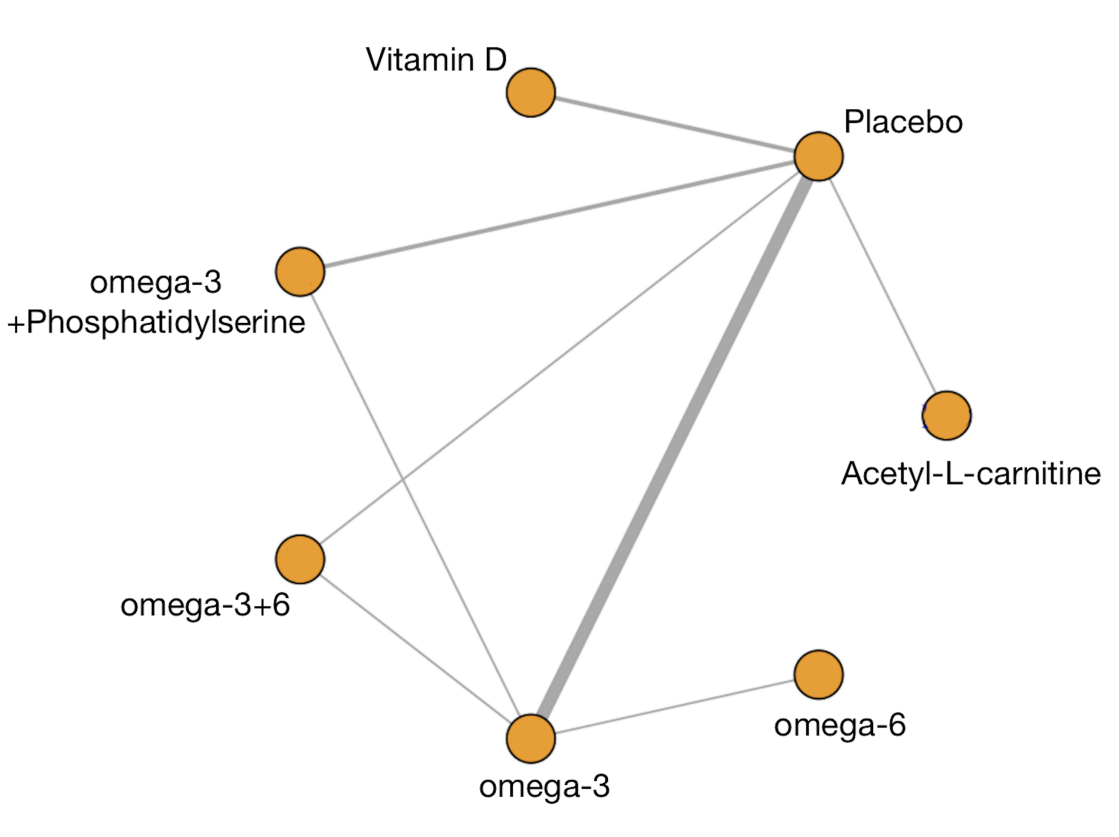
**

1. **Evidence network for total score of Conners’ Parent Rating Scale (network B)**

**(5 intervention nodes, 4 antioxidants, 5 studies, and 428 participants)**

**
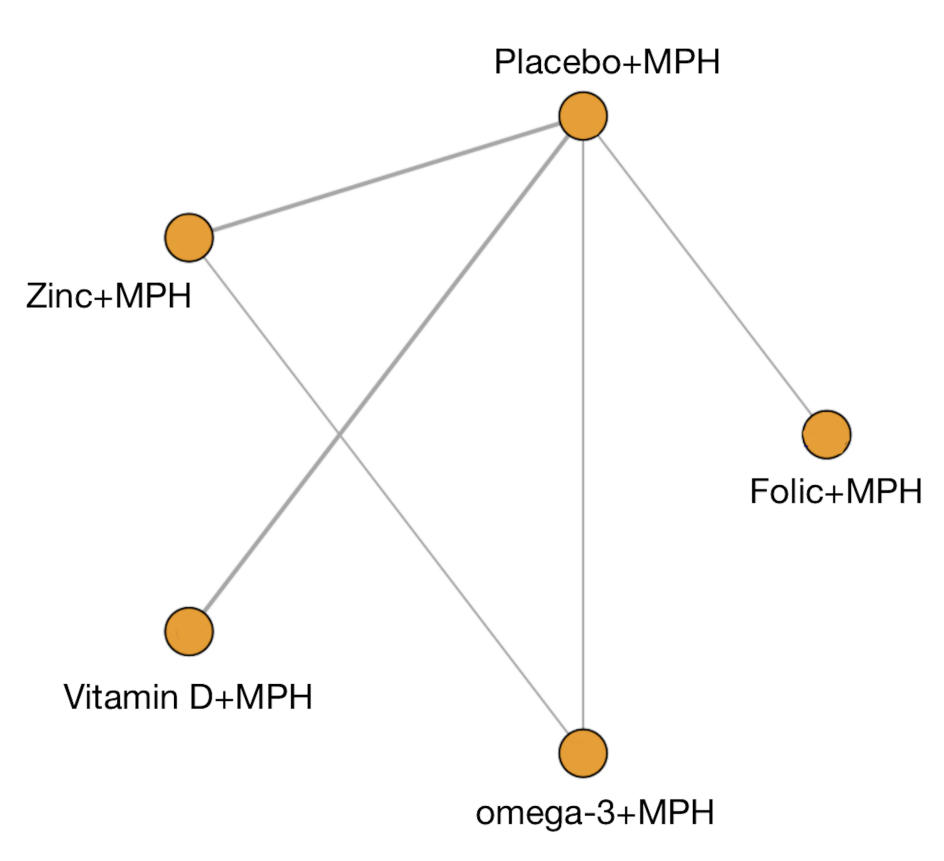
**

1. **Evidence network for attention score of Conners’ Teacher Rating Scale**

**(5 intervention nodes, 4 antioxidants, 5 studies, and 524 participants)**

**
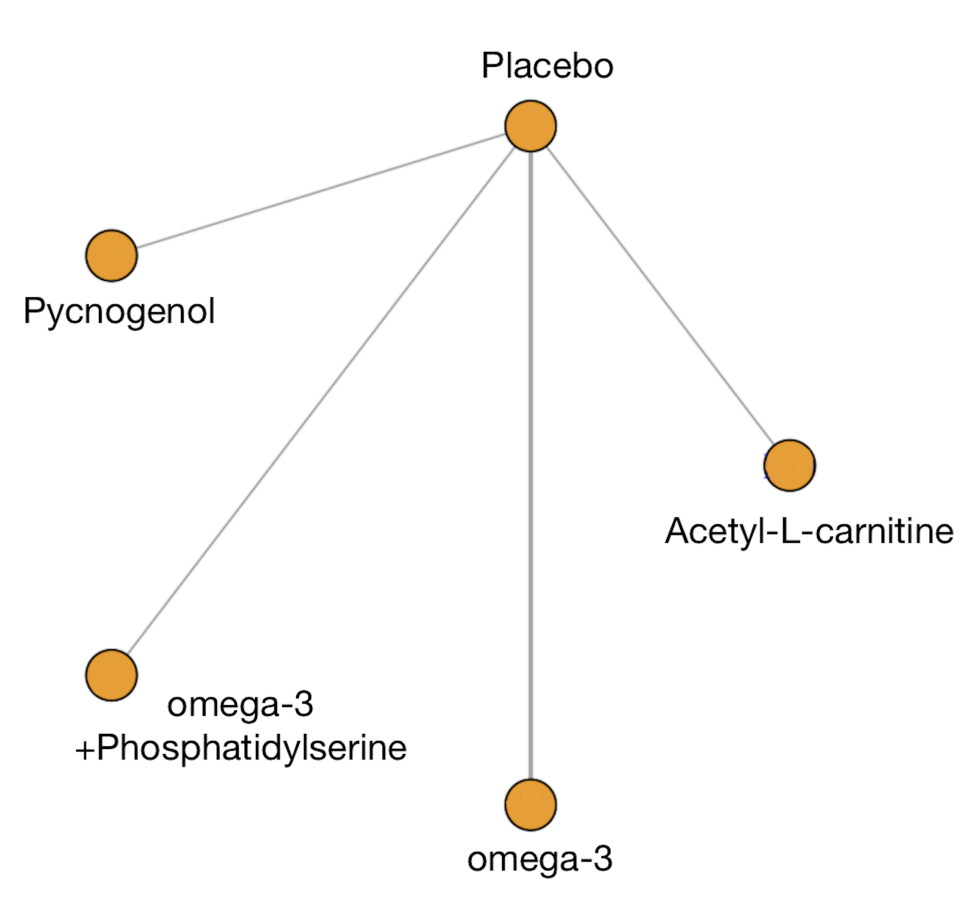
**

1. **Evidence network for hyperactivity score of Conners’ Teacher Rating Scale**

**(7 intervention nodes, 5 antioxidants, 6 studies, and 864 participants)**

**
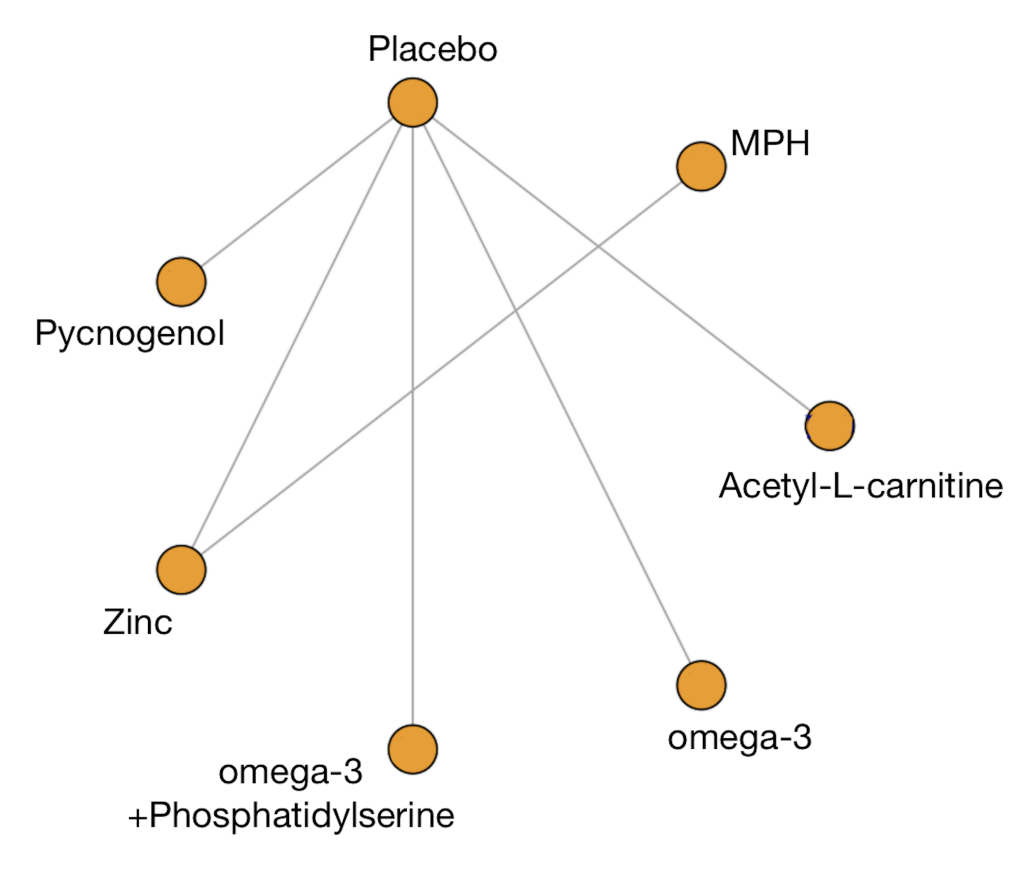
**

1. **Evidence network for total score of Conners’ Teacher Rating Scale**

**(7 intervention nodes, 5 antioxidants, 8 studies, and 930 participants)**

**
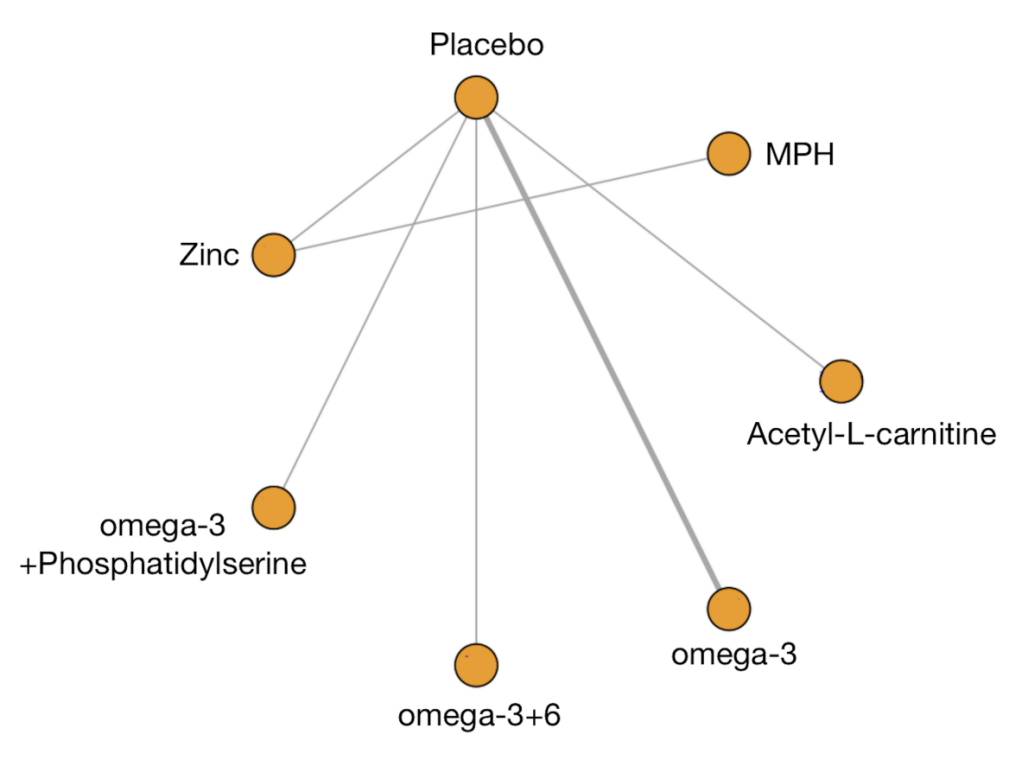
**

1. **Evidence network for attention score of ADHD Rating Scale-Parent**

**(18 intervention nodes, 11 antioxidants, 21 studies, and 1342 participants)**

**
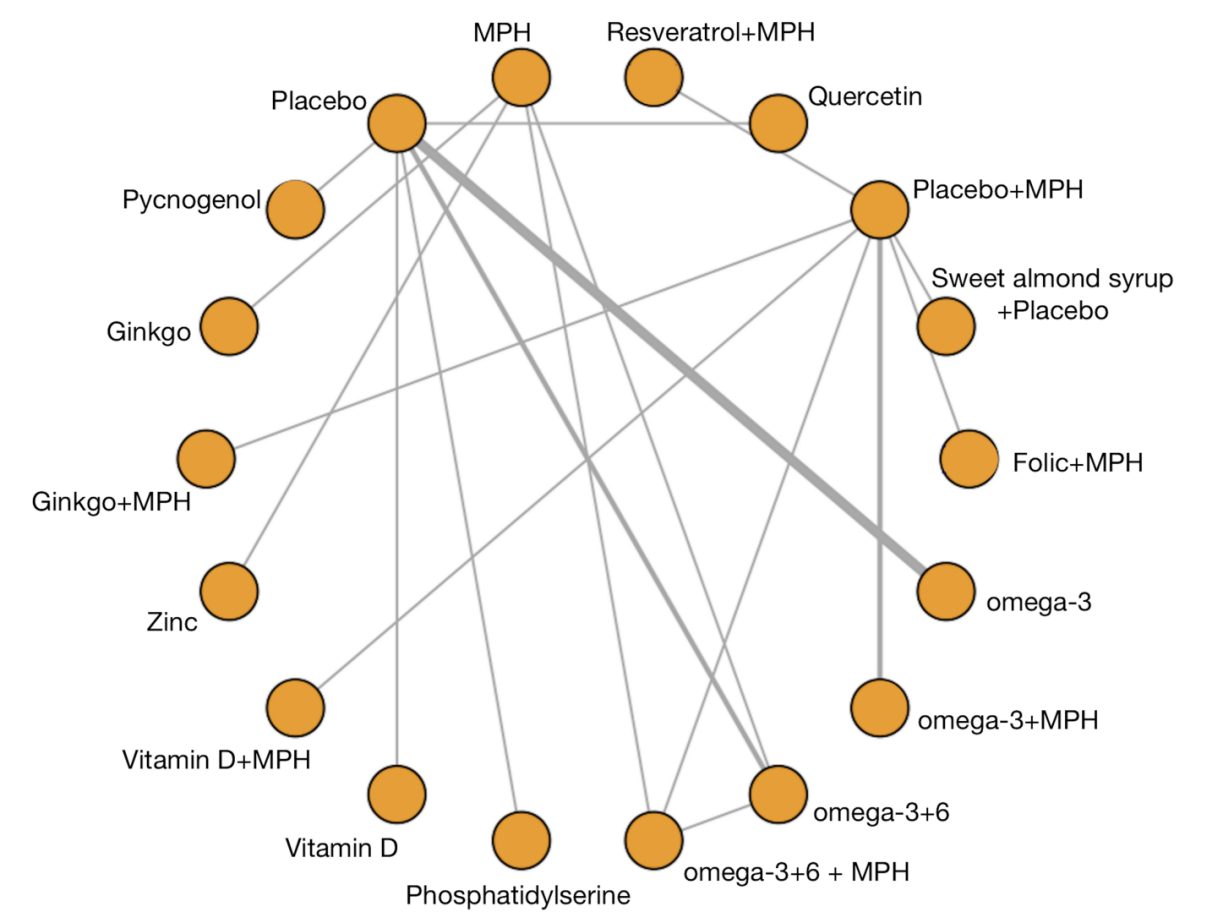
**

1. **Evidence network for hyperactivity score of ADHD Rating Scale-Parent**

**(18 intervention nodes, 11 antioxidants, 20 studies, and 1207 participants)**

**
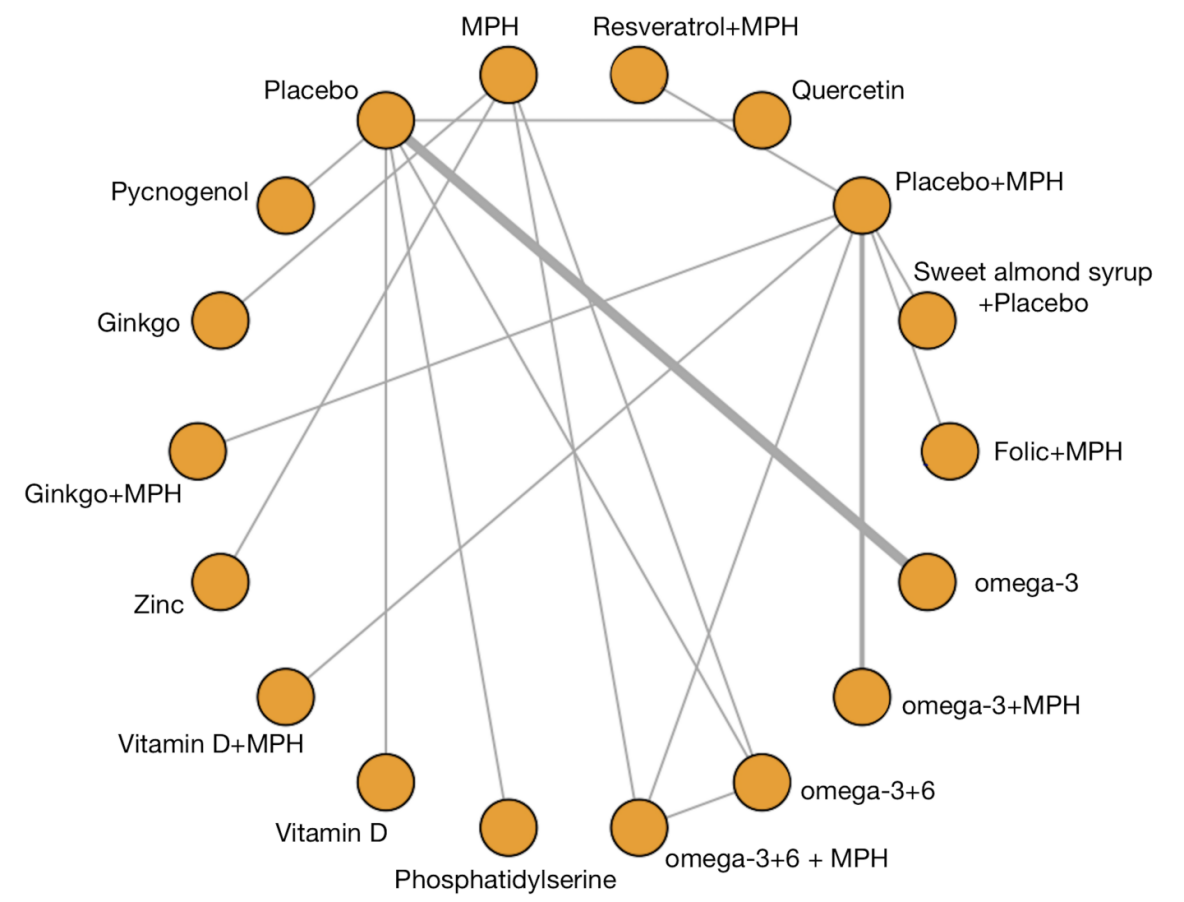
**

1. **Evidence network for total score of ADHD Rating Scale-Parent (network A)**

**(9 intervention nodes, 7 antioxidants, 13 studies, and 887 participants)**

**
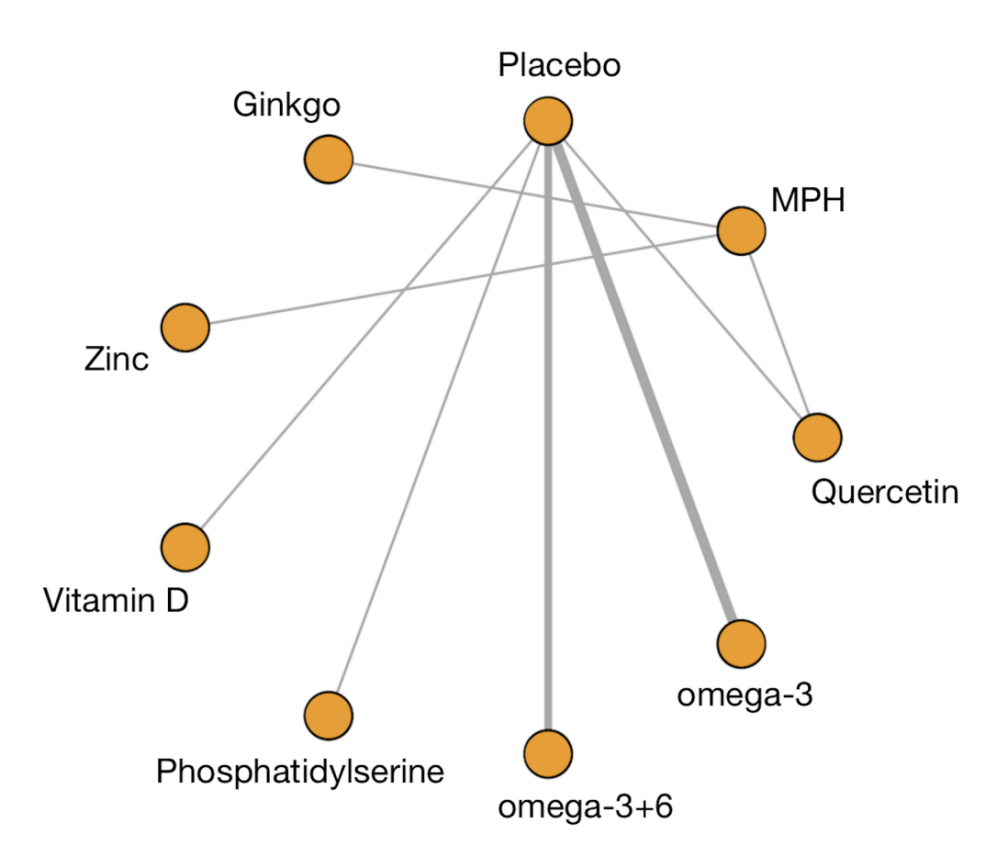
**

1. **Evidence network for total score of ADHD Rating Scale-Parent (network B)**

**(10 intervention nodes, 9 antioxidants, 11 studies, and 580 participants)**

**
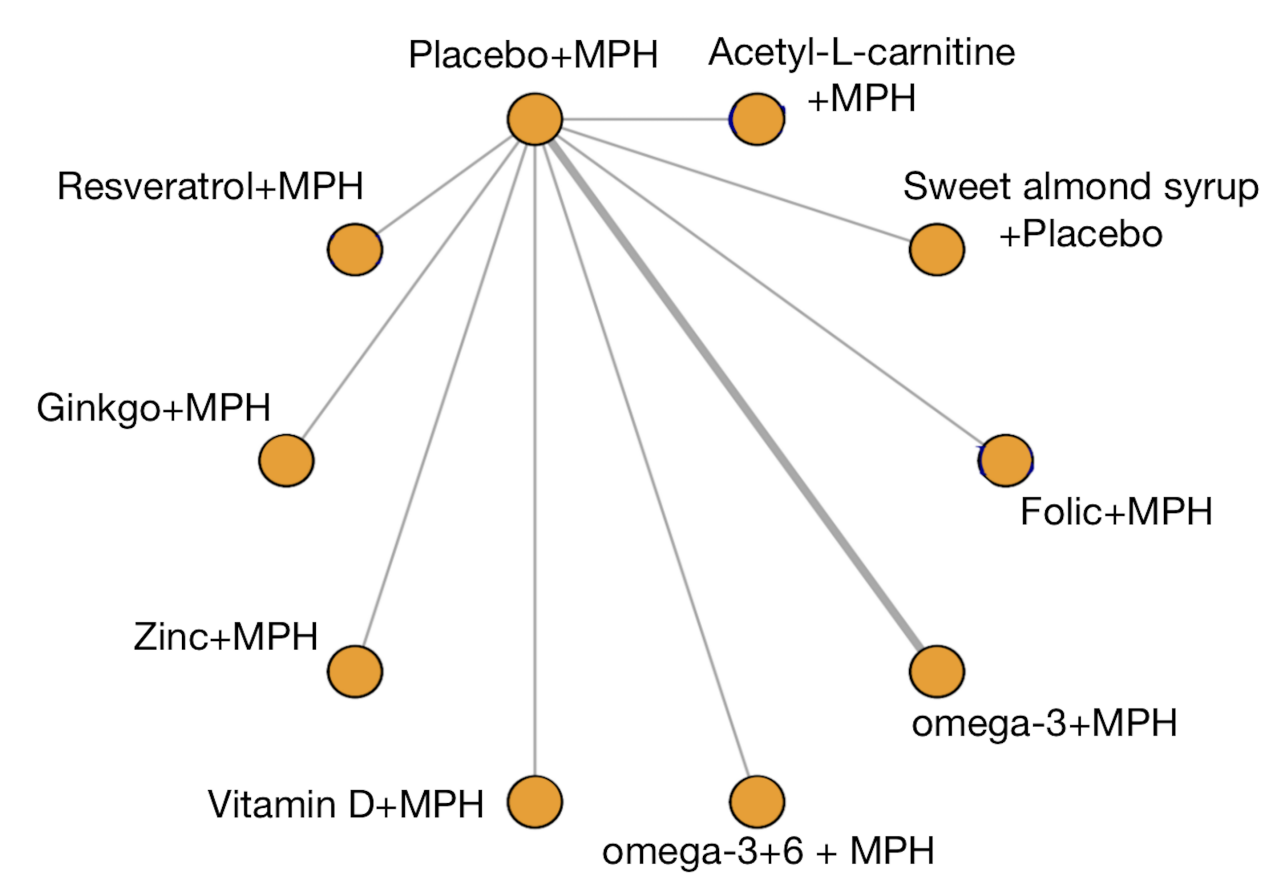
**

1. **Evidence network for attention score of ADHD Rating Scale-Teacher**

**(4 intervention nodes, 3 antioxidants, 3 studies, and 167 participants)**

**
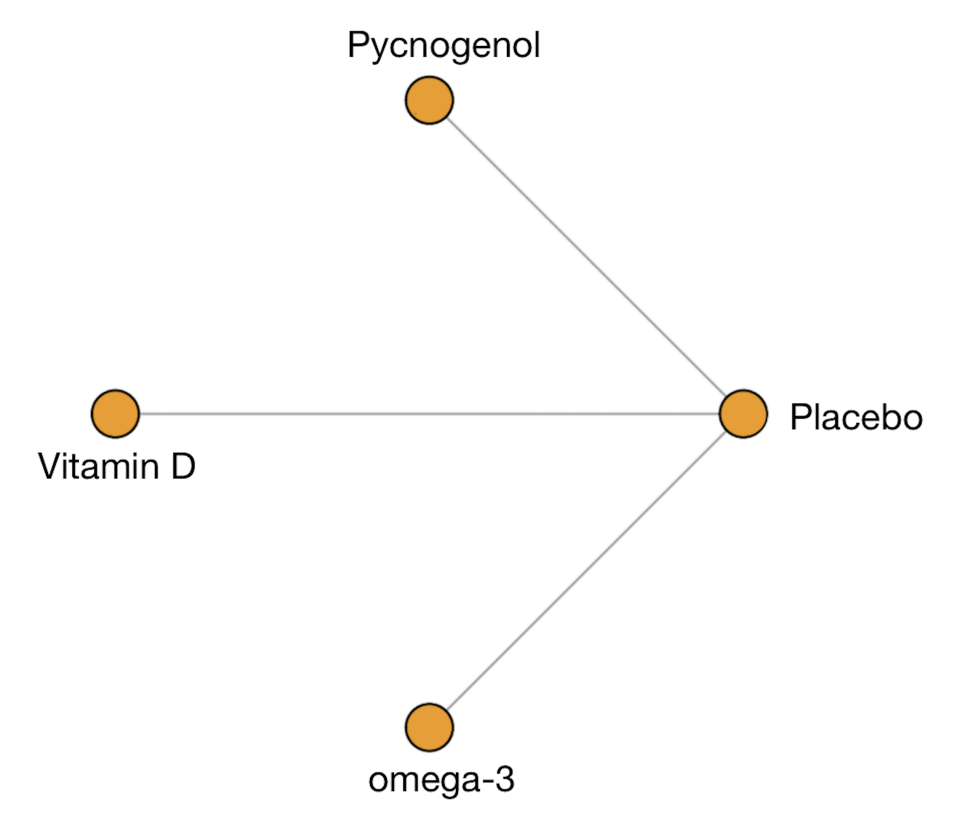
**

1. **Evidence network for hyperactivity score of ADHD Rating Scale-Teacher**

**(4 intervention nodes, 3 antioxidants, 3 studies, and 167 participants)**

**
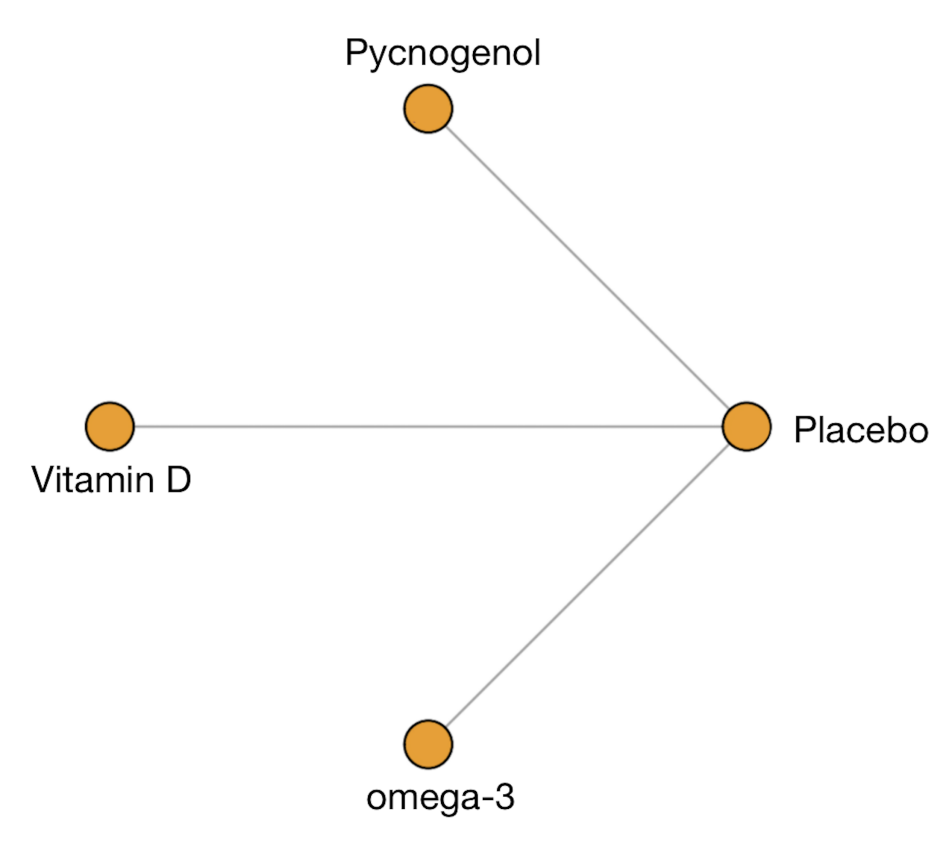
**

1. **Evidence network for total score of ADHD Rating Scale-Teacher (network A)**

**(4 intervention nodes, 3 antioxidants, 3 studies, and 187 participants)**

**
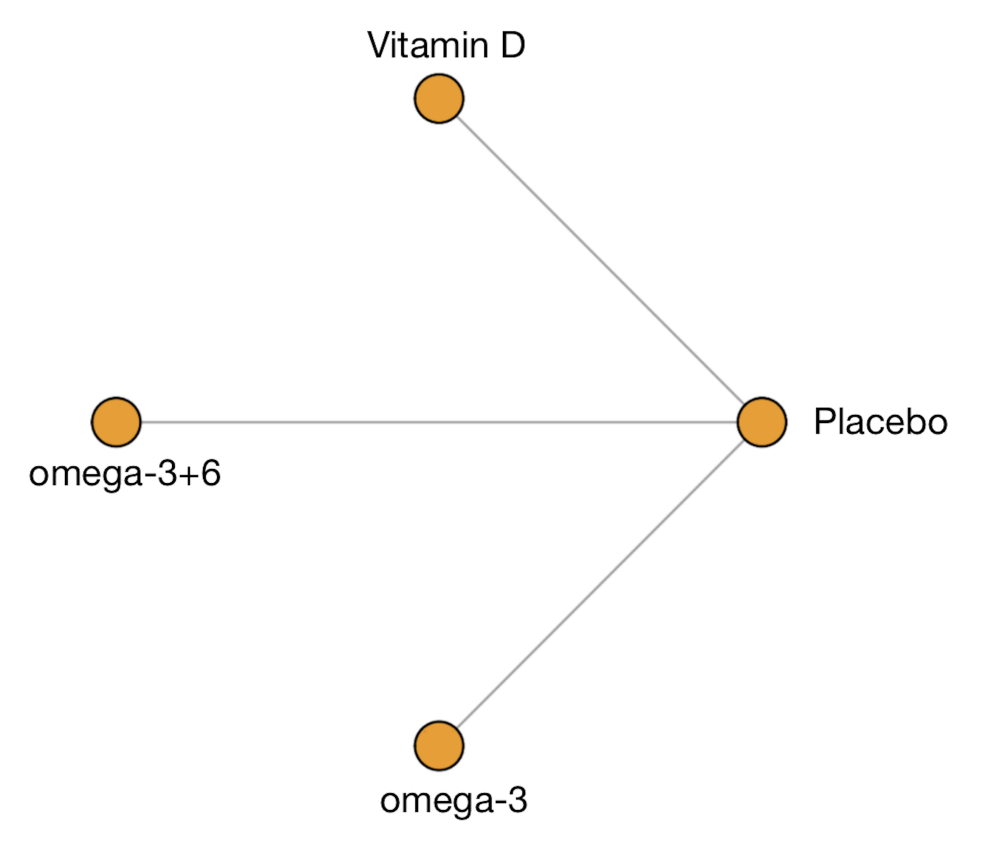
**

1. **Evidence network for total score of ADHD Rating Scale-Teacher (network B)**

**(6 intervention nodes, 5 antioxidants, 5 studies, and 263 participants)**

**
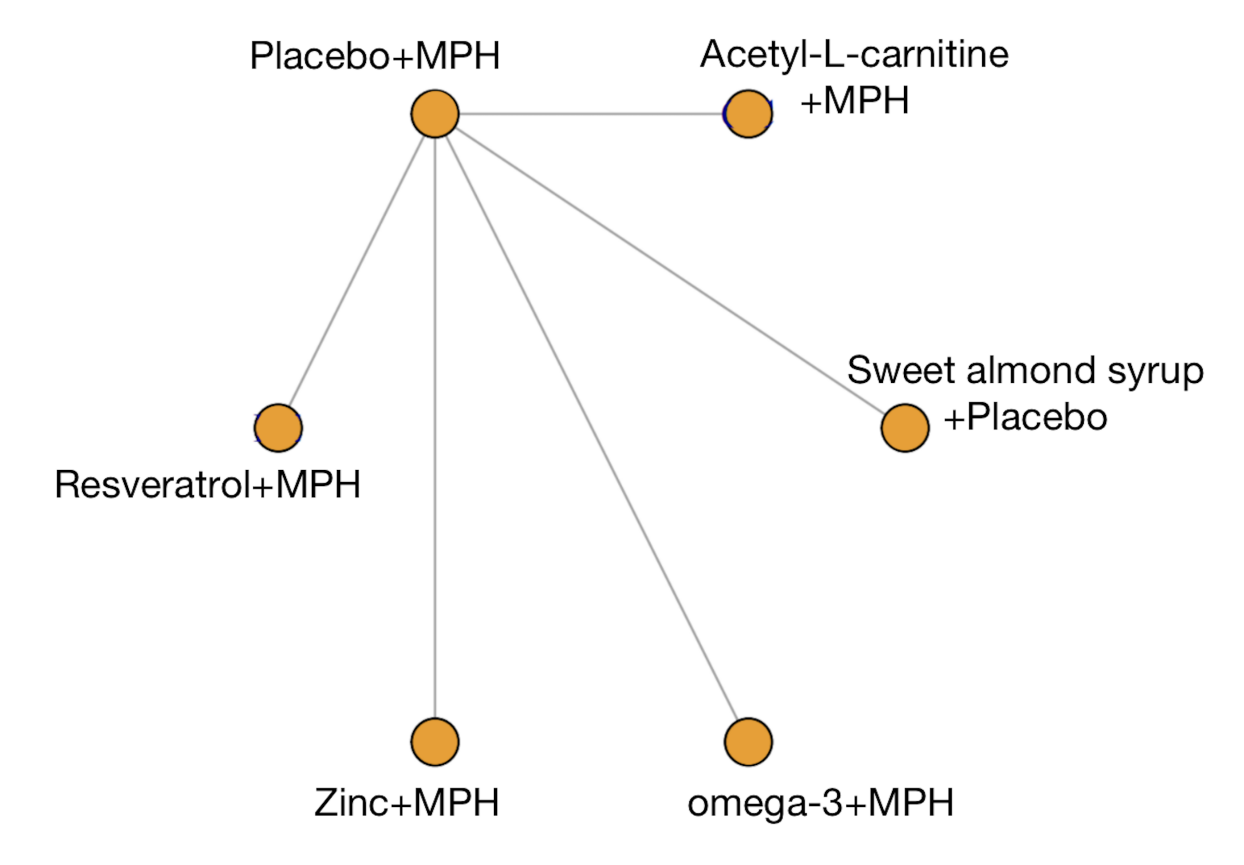
**

1. **Evidence network for total score of ADHD Rating Scale-Teacher (network C)**

**(4 intervention nodes, 3 antioxidants, 3 studies, and 136 participants)**

**
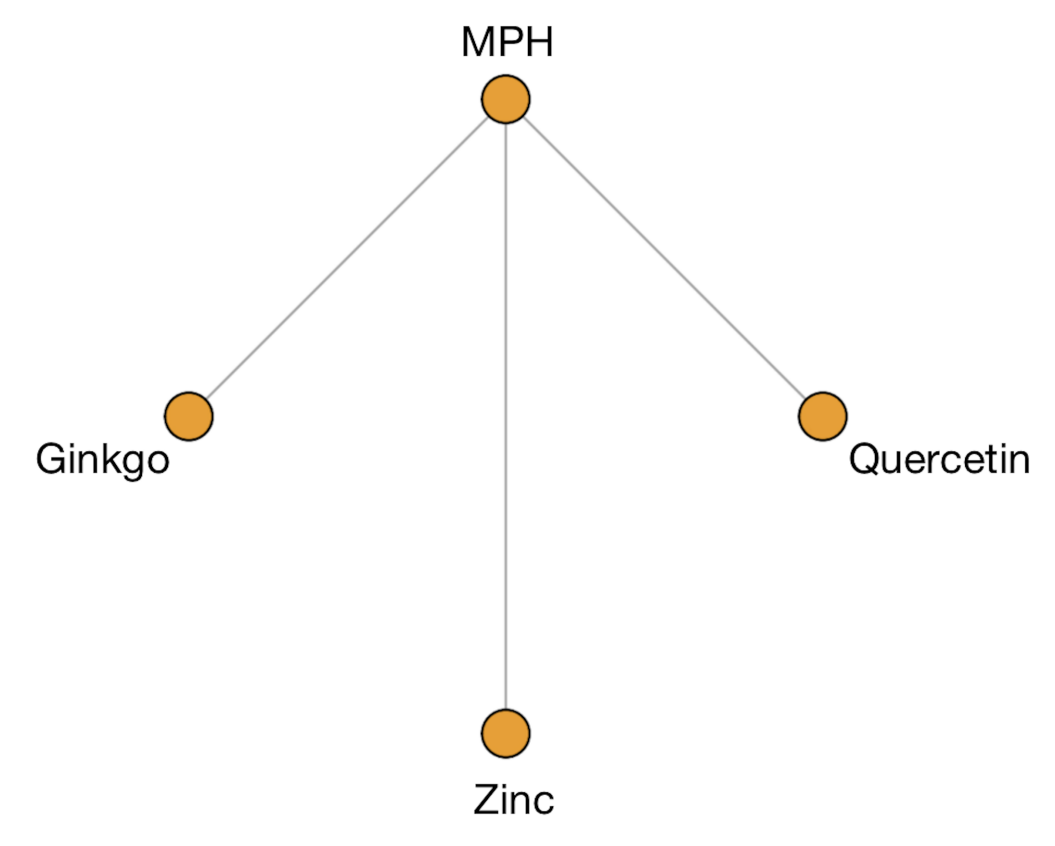
**

1. **Evidence network for Clinical Global Impressions scale**

**(6 intervention nodes, 4 antioxidants, 4 studies, and 331 participants)**

**
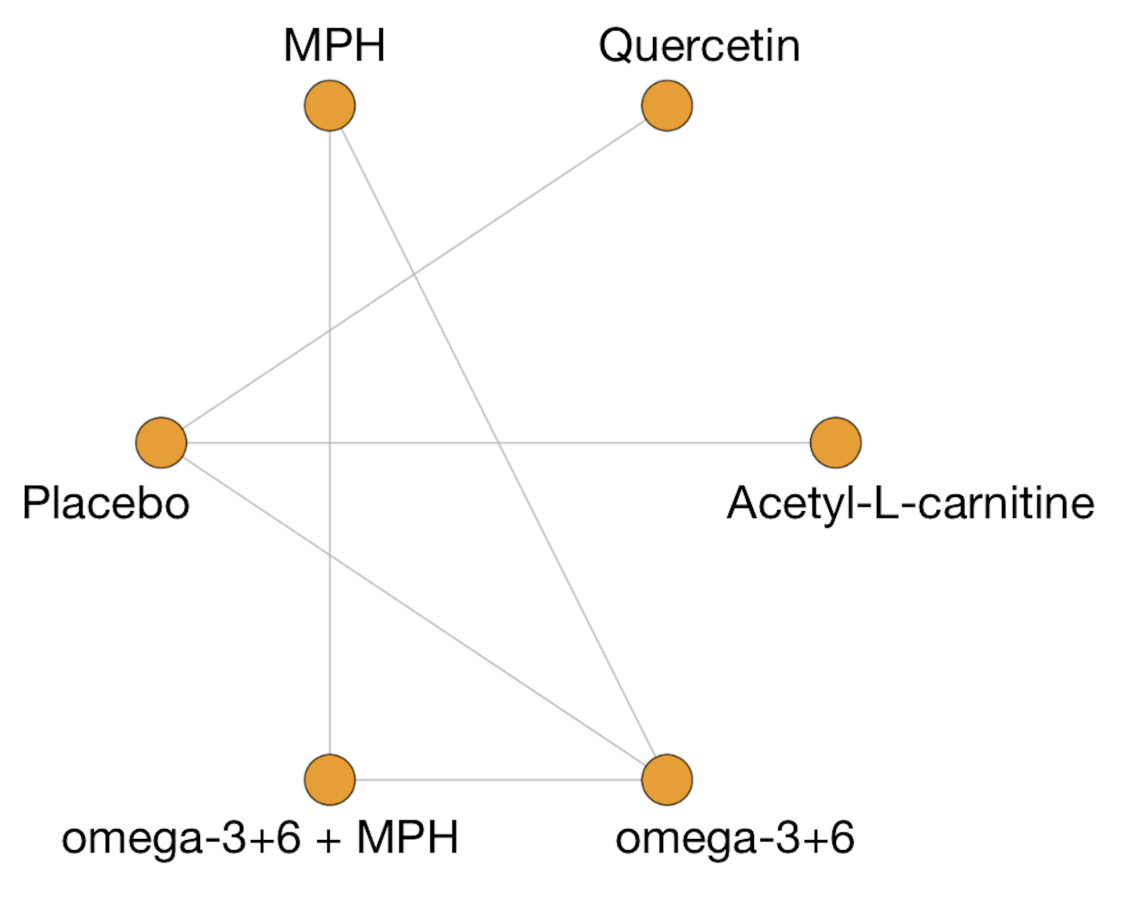
**

1. **Evidence network for Continuous Performance Test**

**(5 intervention nodes, 4 antioxidants, 4 studies, and 230 participants)**


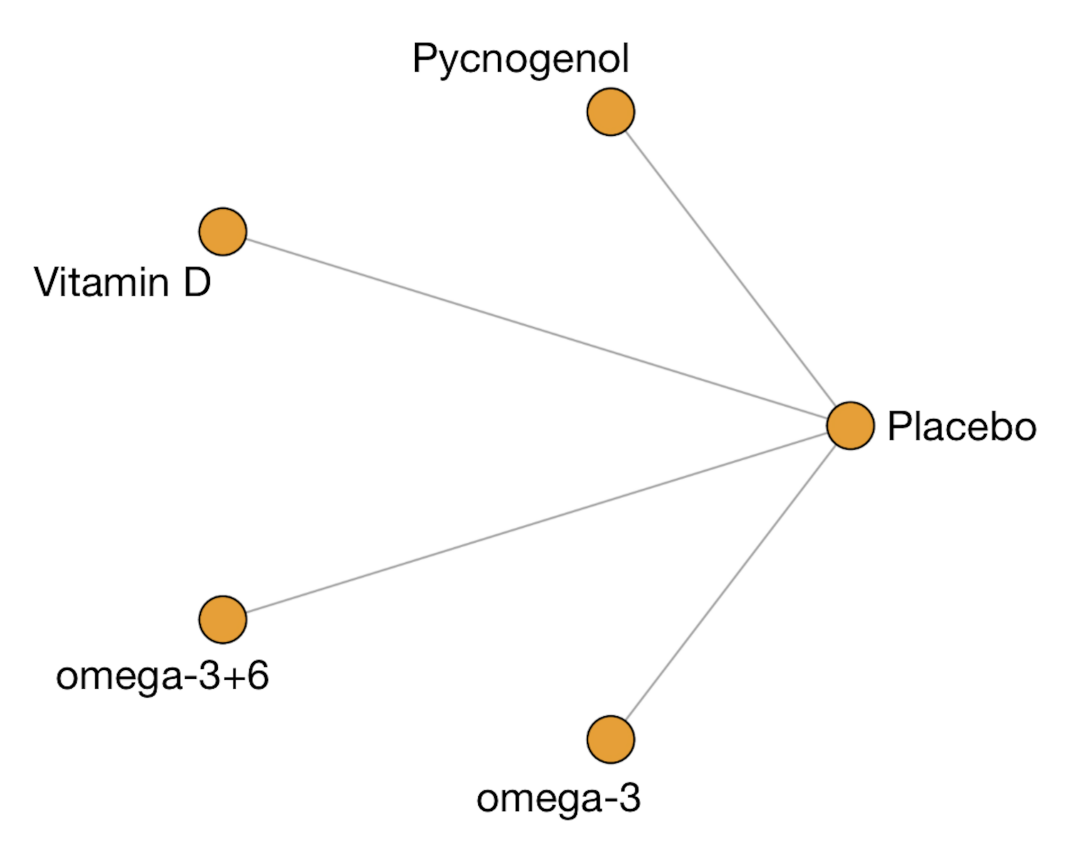


Note: Solid lines represent direct comparisons within randomised trials. omega-3=omega-3 fatty acids, omega-6=omega-6 fatty acids, omega-3+6=omega-3 fatty acids plus omega-6 fatty acids, MPH=Methylphenidate.
