# Supplementary material for: Safety and efficacy of antioxidant therapy in children and adolescents with attention deficit hyperactivity disorder: A systematic review and network meta-analysis
Source: PLoS One. 2024 Mar 28;19(3):e0296926. doi: 10.1371/journal.pone.0296926 (PMC10977718; doi:10.1371/journal.pone.0296926)
Supplement: S1 File — (DOCX) [file pone.0296926.s013.docx]

**Comparative safety and efficacy of antioxidant therapy in children and adolescents with attention deficit hyperactivity disorder: A Systematic Review and Network Meta-Analysis.**

**Citation**

PeiKe Zhou, XiaoHui Yu, Tao Song, XiaoLi Hou. Comparative safety and efficacy of antioxidant therapy in children and adolescents with attention deficit hyperactivity disorder: A Systematic Review and Network Meta-Analysis. PROSPERO 2023 CRD 42023382824 Available from: [https://www.crd.york.ac.uk/prospero/display_record.ph p ID=CRD42023382824](https://www.crd.york.ac.uk/prospero/display_record.ph%20p%20ID=CRD42023382824)

**Review question**

What is the comparative safety and efficacy of antioxidant therapy in the treatment of Attention Deficit Hyperactivity Disorder in children and adolescents?

**Searches**

We will search the following online databases:

(1) PubMed

(2) Embase

(3) Cochrane Library

Date: Articles should be published before November, 2022. Language: There is no limitation on language.

We will evaluate the references section of included studies and systematic reviews/meta-analyses in the field to look for other published/unpublished studies.

**Types of study to be included**

We will include randomized-controlled trials (RCT) and Prospective research, with interventions matching the defined research question.

Both parallel and crossover RCTs will be eligible. For the latter, only the pre-crossover phase will be considered eligible for inclusion.

**Condition or domain being studied**

Attention deficit hyperactivity disorder (ADHD) is a prevalent and persistent psychiatric disorder that emerges early in childhood, with a current prevalence rate of 5 % in children 4–17-years old. ADHD is characterized by symptoms such as inattention, hyperactivity, and impulsivity.

The pathophysiology of ADHD is not completely understood, but as a multifactorial disorder, has been associated with an increase in oxidative stress and neuroinflammation. The imbalance between oxidants and antioxidants and the treatment with medications are two factors that can increase oxidative damage, whereas the comorbidity between ADHD and inflammatory disorders, altered immune response, genetic and environmental associations, and polymorphisms in inflammatory-related genes can increase neuroinflammation.

Central nervous system (CNS) stimulants like methylphenidate and amphetamine are prescribed for patients with ADHD. However, CNS stimulants have various side effects such as arrhythmia, insomnia, irritability, and decreased appetite. Increasing studies are looking for alternative therapies for ADHD, mainly focused on the neuroprotective effects of antioxidants because they may be alternative treatments with fewer side effects, and evidence are emerging on relevancy of antioxidant therapy.

**Participants/population**

Inclusion criteria:

(1) Children and adolescents (< 18 years old).

(2) The primary diagnosis of attention deficit hyperactivity disorder (as diagnosed by a clinician or using any recognized diagnostic criteria according to DSM-III/III-R/IV/IV-TR/5 or ICD-9/10) will be included.

(3) We will not exclude studies based on study setting, participant gender, ethnicity, socioeconomic status.

Exclusion criteria:

Children and adolescents with other serious diseases, such as epilepsy or systemic lupus erythematosus and so on will be excluded.

**Intervention(s), exposure(s)**

We will include the medications with antioxidant mechanisms, such as: Quercetin, Pycnogenol, Ginkgo, zinc, Vitamin, Unsaturated Fatty Acids (Omega-3/6 Fatty Acids) and so on. Other medications with antioxidant mechanisms we are not aware of a priori will also be considered and included. The minimum duration of a treatment arm will be 2 weeks.

We will not restrict based on route of administration, dosing design (fixed-doses or flexible-doses), or doses administered. However, if the antioxidant therapy is Obtained from daily food not the drugs then will be excluded.

Other types of interventions such as Neuropsychological therapy or Exercise therapy will be excluded to avoid added effects from different interventions.

**Comparator(s)/control**

Placebo, antioxidants plus Placebo, or other medicine (such as Methylphenidate) plus Placebo/ antioxidants

**Main outcome(s)**

Primary outcomes

1. Safety: the numbers of adverse events happened during the treatment.
2. Efficacy:

(2.1) ADHD core symptoms, parent rated.

change in total score, Inattention score, and Hyperactivity/Impulsivity score.

Conners parent rating scale (CPRS) and Parent ADHD Rating Scale (ADHD-RS) will be the preferred rating scale.

(2.2) ADHD core symptoms, teacher rated.

change in total score, Inattention score, and Hyperactivity/Impulsivity score.

Conners teacher rating scale (CTRS) and Teacher ADHD Rating Scale (ADHD-RS) will be the preferred rating scale. We will prefer mean change from baseline over endpoint scores. Any other self-made scales will be excluded.

**Measures of effect**

For safety, odds ratio (OR) or relative risk (RR) will be adopted. For efficacy, if studies provide data based on the same rating scale, we will use mean difference (MD) as the effect size index of choice.

**Additional outcome(s)**

Secondary outcomes：

Efficacy：

(1) Clinical Global Impressions scale (CGI): was used to measure illness severity or improvement at the end of the trial. The numbers of patients that CGI score ≤ 2 show that the treatment is effective.

(2) Continuous Performance Test (CPT): measuring focused attention by Hit reaction time (HRT), assessed at baseline and the end of the trial.

**Measures of effect**

We will use odds ratio (OR) or relative risk (RR) for dichotomous variables and mean difference (MD) for continuous variables.

**Data extraction (selection and coding)**

Study selection and data extraction will be conducted by two reviewers and disagreements will be solved through discussion with a third reviewer for conflicts. Titles and abstracts will be screened, and full texts of selected records will be retrieved to determine eligibility.

Data will be coded in an Excel spreadsheet. We will extract title, authors, year of the study, journal, study design/type of RCT, follow up time, industry or academic funding/support, number of participants (total and by group), participants characteristics (age, sex and race/ethnicity) and treatment characteristics (medication administered, daily dose administered, dosing regimen adopted, duration of treatment) characteristic, time of outcome measurement and outcome measures.

We will extract data considering the baseline and endpoint reported by the trial. If multiple timepoints are reported, we will extract outcomes for the average.

We will use published SD when available, but if not reported we will: (1) retrieve from p-values, t-values, confidence intervals or standard errors as described in the Cochrane Handbook; (2) attempt to retrieve SD from study authors; (3) impute missing data with the average SD.

**Risk of bias (quality) assessment**

We will assess study quality of RCTs with the Cochrane risk-of-bias tool (RoB) which evaluates bias arising from: the randomization process, deviations from intended interventions, missing outcome data, measurement of the outcome and selection of the reported result.

**Strategy for data synthesis**

A network meta-analysis performed by R (4.2.1) was carried out to compare different treatment modalities, a network map was obtained for each study result, which graphical representation of treatment (nodes) and comparisons (lines). We intend to use R (4.2.1) for the meta-analysis. Using a random effects model and a fixed effects model. In the event of inconsistent results from the two models we prefer to report both results; otherwise, we expect to adopt the results of the random effects model Binary variables were analyzed by estimating the odd ratio (OR) with 95% confidence interval (95% CI). Continuous variables were analyzed employing the mean difference (MD) with 95% CI.

**Analysis of subgroups or subsets**

For efficacy, different interventions may adopt different evaluation rating scale. We will conduct subgroup analysis according to different interventions.

**Contact details for further information**

PeiKe Zhou [zhoupeike@s.dlu.edu.cn](mailto:zhoupeike@s.dlu.edu.cn)

**Organisational affiliation of the review**

Affiliated ZhongShan Hospital of Dalian University

[https://www.dlhospital.com](https://www.dlhospital.com/)

**Review team members and their organisational affiliations**

Dr PeiKe Zhou. Affiliated ZhongShan Hospital of Dalian University

Dr XiaoHui Yu. Affiliated ZhongShan Hospital of Dalian University

Dr Tao Song. Affiliated ZhongShan Hospital of Dalian University

Dr XiaoLi Hou. Affiliated ZhongShan Hospital of Dalian University

**Type and method of review**

Intervention, Meta-analysis, Network meta-analysis, Systematic review

**Anticipated or actual start date**

26 October 2022

**Anticipated completion date**

30 September 2023

**Funding sources/sponsors**

None.

**Grant number(s)**

State the funder, grant or award number and the date of award

None.

**Conflicts of interest**

None.

**Language**

English

**Country**

China

**Stage of review**

Review Ongoing

**Subject index terms status**

Subject indexing assigned by CRD

**Subject index terms**

Adolescent; Antioxidants; Attention Deficit Disorder with Hyperactivity; Child; Humans; Network Meta-Analysis

**Date of registration in PROSPERO**

16 January 2023

**Date of first submission**

05 January 2023

**Stage of review at time of this submission**

| Date of registration in PROSPERO  16 January 2023 |  | |
| --- | --- | --- |
| Date of first submission  05 January 2023 |  |  |
| Stage of review at time of this submission |  |  |
| Stage | Started | Completed |
| Preliminary searches | Yes | No |
| Piloting of the study selection process | Yes | No |
| Formal screening of search results against eligibility criteria | No | No |
| Data extraction | No | No |
| Risk of bias (quality) assessment | No | No |
| Data analysis | No | No |
